# Health care's response to climate change: a carbon footprint assessment of the NHS in England

**DOI:** 10.1016/S2542-5196(20)30271-0

**Published:** 2021-02-10

**Authors:** Imogen Tennison, Sonia Roschnik, Ben Ashby, Richard Boyd, Ian Hamilton, Tadj Oreszczyn, Anne Owen, Marina Romanello, Paul Ruyssevelt, Jodi D Sherman, Andrew Z P Smith, Kristian Steele, Nicholas Watts, Matthew J Eckelman

**Affiliations:** a NHS England & NHS Improvement, London, UK; b Arup Group, London, UK; c UCL Energy Institute, University College London, London, UK; d Institute for Global Health, University College London, London, UK; e School of Earth and Environment, University of Leeds, Leeds, UK; f Department of Anesthesiology, Yale University, New Haven, CT, USA; g Department of Civil and Environmental Engineering, Northeastern University, Boston, MA, USA

## Abstract

**Background:**

Climate change threatens to undermine the past 50 years of gains in public health. In response, the National Health Service (NHS) in England has been working since 2008 to quantify and reduce its carbon footprint. This Article presents the latest update to its greenhouse gas accounting, identifying interventions for mitigation efforts and describing an approach applicable to other health systems across the world.

**Methods:**

A hybrid model was used to quantify emissions within Scopes 1, 2, and 3 of the Greenhouse Gas Protocol, as well as patient and visitor travel emissions, from 1990 to 2019. This approach complements the broad coverage of top-down economic modelling with the high accuracy of bottom-up data wherever available. Available data were backcasted or forecasted to cover all years. To enable the identification of measures to reduce carbon emissions, results were disaggregated by organisation type.

**Findings:**

In 2019, the health service's emissions totalled 25 megatonnes of carbon dioxide equivalent, a reduction of 26% since 1990, and a decrease of 64% in the emissions per inpatient finished admission episode. Of the 2019 footprint, 62% came from the supply chain, 24% from the direct delivery of care, 10% from staff commute and patient and visitor travel, and 4% from private health and care services commissioned by the NHS.

**Interpretation:**

This work represents the longest and most comprehensive accounting of national health-care emissions globally, and underscores the importance of incorporating bottom-up data to improve the accuracy of top-down modelling and enabling detailed monitoring of progress as health systems act to reduce emissions.

**Funding:**

Wellcome Trust.

## Introduction

Climate change threatens to disrupt health systems' ability to deliver high-quality care and undermine the past 50 years of gains in public health, with more intense heatwaves, higher risks of flooding and damaging storms, and a changing pattern of emerging infectious diseases.^1^ Responsible for some 4–5% of global greenhouse gas emissions, the health-care sector has a vital role to play in climate change mitigation efforts, which will not only result in substantial reductions in emissions, but can often lead to enhanced patient care, staff satisfaction, and cost savings.^1^, ^2^ These benefits occur in part by preventing the initial health impacts of climate change, while also improving wellbeing through health co-benefits, such as cleaner air, increased physical activity, and more nutritious diets.^3^ Importantly, these co-benefits can help to offset part of the costs of mitigation interventions.^3^, ^4^

Numerous laudable examples of mitigation in the health-care sector exist around the world, with interventions ranging from on-site renewable energy projects or low-carbon procurement strategies through to digital redesign and changes in clinical practice. WHO and several non-governmental organisations have worked to elevate these efforts, looking to spread best practices and engage stakeholder groups. At a national level, successful mitigation strategies require national-level data on emissions status and trends. To this end, carbon footprints have been published for health-care systems in Australia, Austria, Canada, China, Japan, and the USA, alongside international estimates.^1^, ^2^, ^5^, ^6^, ^7^, ^8^, ^9^, ^10^, ^11^, ^12^ These studies have identified general patterns of contributions among health-care activities in each country, which can form a baseline for long-term mitigation planning.

The UK's National Health Service (NHS) is the largest publicly funded health system in the world. Every year, the NHS delivers 17 million inpatient admissions from more than 200 hospital trusts, more than 270 million primary care appointments from nearly 7000 general practices, and prescribes more than 1·1 billion items every year. As the largest single-payer health-care system in the world and the biggest employer in Europe, England's NHS has the opportunity to leverage its size and influence to drive its own emissions reductions and serve as a model for others.^13^, ^14^, ^15^ The Sustainable Development Unit was created by the NHS in 2008 to meet the government's commitments under the UK Climate Change Act, conducting its first assessment of the NHS's carbon footprint that year. Regularly updated and improved upon, these assessments now constitute the longest-running effort to quantify health-care-related greenhouse gas emissions in the world, and are notably the only national-level analyses carried out by a public agency with institutional support, rather than by independent researchers.

**Research in context**

**Evidence before this study**

While the world responds to the COVID-19 public health emergency, climate change, driven by anthropogenic greenhouse gas emissions, remains a long-term threat to the health and wellbeing of world populations, and the increasing pressure it poses on health-care systems threatens to undermine the past 50 years of gains in public health.

The health sector has been shown to contribute to 4–5% of global greenhouse gas emissions, and its response is key not only to mitigate climate change, but especially to ensure that the health benefits of this transition are maximised. In line with this goal, researchers in Australia, Austria, Canada, China, Japan, and the USA have estimated the emissions profile of national health systems, and multicountry studies have been done by research institutions and non-governmental organisations. In England, the Sustainable Development Unit has published regular updates of the National Health System (NHS)'s carbon footprint since 2008, making it the longest-running and only such effort carried out by a governmental body rather than independent researchers, for what is the largest publicly funded health system in the world.

**Added value of this study**

The NHS carbon footprint presented here is aligned with the Greenhouse Gas Protocol, covering Scopes 1, 2, and 3 as well as other personal travel (patient and visitor travel) that would not normally be included in an organisation's footprint. It uses a hybrid modelling approach that takes advantage of the accuracy associated with bottom-up data and the broad coverage of top-down economic input–output modelling. The disaggregation of greenhouse gas emissions by type of clinical activity, as well as per unit of health-care provision, further enables the identification of long-term mitigation interventions. This work therefore represents the most comprehensive and sophisticated health-care footprinting effort to date.

In 2019, the carbon footprint of the NHS in England totalled 25·0 megatonnes of carbon dioxide equivalent, of which 62% came from its supply chain (including emissions imported in foreign goods and services) and only 24% from the delivery of care scope, stressing the importance of a coordinated international effort to achieve a robust decarbonisation of health care. Importantly, 10% of emissions came from travel to and from NHS sites by patients, visitors, and staff commute, over which the health systems can have a considerable degree of influence through innovations in models of care.

**Implications of all the available evidence**

Due to its sizeable contribution to global greenhouse gas emissions and the health benefits of transitioning towards a low-carbon economy, health systems have a central role to play in climate change mitigation efforts. This work lays out a robust methodology for health-care system footprinting that can help to guide efforts in other countries as they monitor their own health-care emissions. It also underscores the importance of a comprehensive and broad scope approach for tracking the full impact of health-care provision and the need for investing in bottom-up data collection through robust and validated information systems to increase the accuracy and resolution of emissions accounting and inform focused interventions.

This Article represents a collaboration between the NHS and the *Lancet* Countdown and presents results for the most recent carbon footprint estimates for the NHS, covering emissions from 1990 to 2019, alongside a detailed description of the hybrid accounting method taken.

## Methods

### Study design

NHS England's emissions were calculated using a hybrid accounting method that combines two approaches: (1) location-generic (top-down) results for categories that can only be measured in economic terms or that are too complex to model physically, and (2) product-specific and location-specific (bottom-up) results for emissions categories that can be measured and described physically (appendix 1 pp 2–3). Hybrid modelling is commonly used in life-cycle assessment and resource footprinting, representing a compromise that takes maximum advantage of the accuracy associated with bottom-up physical modelling and of the broad coverage of a system-wide supply-chain top-down modelling.^16^ The analysis covers the period 1990–2019, allowing for benchmarking with the Climate Change Act. Emissions estimates for specific years, depending on the emissions category and data source, were backcasted (ie, modelled for previous years) or forecasted to the remaining years as noted (appendix 1 pp 2–3, 43–45).

**Figure 1:**
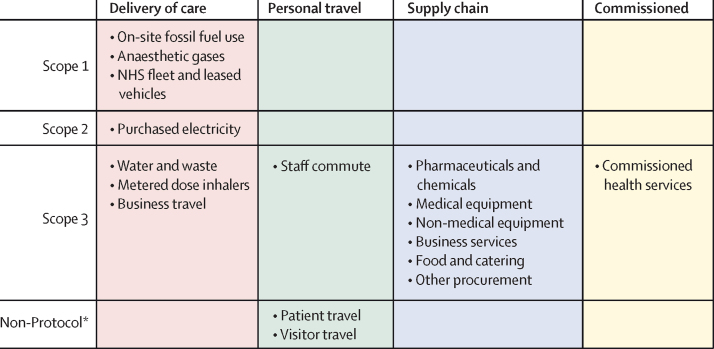
Sources of emissions organised by Greenhouse Gas Protocol Scopes and by NHS emissions categories Columns represent NHS emission categories and rows the Greenhouse Gas Protocol Scopes. NHS=National Health Service. *Not normally considered within the Greenhouse Gas Protocol.

The footprint analysis covers greenhouse gas emissions (carbon dioxide [CO_2_], methane [CH_4_], nitrous oxide [N_2_O], and some categories of fluorinated gases) under Scopes 1, 2, and 3 of the Greenhouse Gas Protocol, as well as personal travel emissions, which are not normally considered within the Greenhouse Gas Protocol but over which the NHS has considerable influence (figure 1).^17^ Scope 1 covers direct emissions from health-care facilities, Scope 2 covers emissions from purchased energy such as electricity and steam, and Scope 3 covers all other emissions. Emissions were further organised into four categories: delivery of care, personal travel, supply chain, and commissioned services—ie, clinical services procured from private health-care providers (figure 1). Delivery of care Scope 3 encompasses emissions that occur during use and therefore can be strongly influenced by how care is provided. For each category, emissions were calculated by multiplying the quantity of consumption (eg, electricity use) by the corresponding emissions factor (CO_2_ equivalent [CO_2_e] mass of emissions per consumption unit). Results are presented for the NHS in England as a whole and disaggregated by organisation. To account for changes in the volume of health-care provision, results were also normalised against the number of inpatient finished admission episodes (FAEs; defined as a single inpatient admission in the year), health-care expenditure, and total population. Additional methodological details are provided in appendix 1 (p 42).

### Building emissions

Building emissions include Scope 1 fuel use, Scope 2 electricity use, and Scope 3 upstream energy emissions (eg, from emissions from oil and gas extraction, and distribution losses). Data from the Estates Return Information Collection (ERIC) system—a comprehensive national-level database—were used to estimate emissions from NHS buildings. ERIC collates data from NHS trusts and ambulance trusts within England, including the consumption of energy, water, and limited other goods for all buildings and NHS-leased sites, covering 24 million m^2^ of hospitals and other clinical facilities across the country (appendix 1 pp 4–6), and is subject to robust validation.^18^ Importantly, it does not include other health-care buildings such as those for primary care, sites smaller than 150 m^2^ with fewer than ten inpatient beds, or office buildings of non-clinical organisations. Energy use for these excluded building types was estimated for 2015 and comprised approximately 15% of total NHS energy use (appendix 1 p 7). ERIC reporting requirements and NHS structures have changed over time and an annual adjustment was included to account for this. Corresponding emissions factors for fuels and electricity were taken from the UK Government energy (Department for Business, Energy & Industrial Strategy [BEIS]) and environment (Department for Environment, Food & Rural Affairs) ministry publications for 2002–19, and from company reporting guidance for older calculations (appendix 1 p 8).^19^, ^20^ Electricity generated by on-site combined heat and power plants was accounted for using fossil fuel consumption emission factors rather than grid electricity factors.

### Anaesthetic gases and metered dose inhalers

In addition to emissions associated with manufacturing (counted in the supply chain), some pharmaceuticals have emissions released on site (Scope 1), such as anaesthetic gases, inhaled medicine propellants, and other medical and surgical gases. Volatile anaesthetics (sevoflurane, isoflurane, and desflurane) and N_2_O are potent greenhouse gases that are routinely released unmetabolised into the atmosphere. This analysis used four categories of data sources to estimate these: supplier data from distribution or manufacturing companies, voluntary health-facility reporting, NHS pharmacy electronic data repository (volatiles only), and dental clinic N_2_O data from work commissioned by Public Health England.^21^ UK data were scaled to England by population, and all bottom-up data were extrapolated to England by occupied bed-days. In each case, Global Warming Potential factors for the volatiles were taken from Sulbaek Anderson et al^22^ and for N_2_O from the Intergovernmental Panel on Climate Change's Fifth Assessment Report (IPCC AR5).^23^

Metered dose inhalers (MDIs) use chlorofluorocarbon or hydrofluorocarbon propellants that do not undergo metabolic transformation and are released unchanged into the atmosphere. The emissions occur wherever patients use them, and are treated as Scope 3. Emissions data based on number of prescriptions have been reported to the National Atmospheric Emission Inventory since 2006, and were backcasted to 1990 using population data and assuming no change in inhaler use per capita before 2006 (appendix 1 pp 43–45).^24^ Corresponding emission factors are from the IPCC AR5.^23^

### Supply chain emissions and commissioned health services

Multiregion input–output (MRIO) modelling was used to determine the contribution of Scope 3 supply chain emissions to the carbon footprint of the NHS in England. The methodology uses the UK MRIO model alongside the Public Expenditure Statistical Analysis Supply and Use tables from HM Treasury to derive emissions associated with first-tier procurement categories.^25^, ^26^, ^27^ In input–output modelling, consumption is represented by monetary expenditures rather than physical quantities. The NHS's internal spending information does not capture breakdown by economic sector and so was not used; instead, total spending on health care tracked by HM Treasury was proportioned using the transaction matrix in the UK MRIO model. This matrix maps the total NHS expenditure to 106 sectors for each of four world regions (UK, China, EU, and Rest-of-World regions; appendix 1 pp 9–20). Emissions factors for each of the resulting 424 sectors, covering all Kyoto Protocol greenhouse gases including all embodied, cradle-to-gate emissions, were calculated from the UK MRIO satellite accounts (appendix 1 pp 21–31).^28^ Emissions were then calculated by multiplying sectoral expenditure data and the corresponding emissions factors.

The raw results were then aggregated using a concordance-based approach. This process maps emissions into 19 expenditure categories. Nine of these relate to Scope 1 and 2 emissions and were removed as they are accounted for elsewhere via bottom-up calculations (appendix 1 pp 32–34). Discontinuities resulting from sector reclassification or MRIO model updates were replaced with interpolated values to conform with long-term trends. Beyond its supply chain, the NHS commissions health and care services from other providers including private hospitals and third parties—eg, for community services. Emissions (Scope 3) associated with these expenditures were estimated using the UK MRIO model, matched to the economic sector UK Human Health Activities.

### Travel emissions

Travel emissions include fleet travel (Scope 1), business travel and staff commuting (Scope 3), and personal patient and visitor travel (additional induced emissions not covered by the Greenhouse Gas Protocol). Emissions from fleet travel using vehicles owned or leased by the NHS or business travel otherwise funded by the health services were accounted for using the top-down UK MRIO model based on expenditures on travel (see section on supply chain emissions), while emissions from personal travel by staff (commuting), as well as patients and visitors to and from NHS sites were accounted using bottom-up data (appendix 1 p 35). For staff, bottom-up data were gathered from the Department for Transport in 2015 and 2019 for average distance travelled per trip (appendix 1 p 36). Patient and visitor travel data were recorded in the UK National Travel Survey (appendix 1 p 37). Emissions factors from BEIS were mapped to each mode of travel, including both Scope 1 (direct emissions) and Scope 3 (well-to-tank emissions; appendix 1 p 38).^19^

### Allocation to organisations

Carbon emissions for each NHS emissions category were further disaggregated to six organisation types—ambulance, community, mental health, acute, primary care, and non-clinical support activities—using a combination of top-down and bottom-up data as appropriate to the emissions category. For bottom-up estimates, energy use, travel estimates based on activity and numbers of journeys, anaesthetic gas use, and MDI numbers were allocated on the basis of internal data; for top-down estimates, the share of expenditures were used.^29^ All data used for allocating emissions were specific to 2018–19 operations and scaled to 2019.

### Uncertainty

Due to the hybrid approach combining multiple bottom-up datasets with top-down MRIO-based results, it was not feasible to conduct a comprehensive uncertainty analysis, such as the Monte Carlo simulation-based analyses done for MRIO-only studies of health-care emissions.^5^, ^6^ Bottom-up datasets used for this analysis, including building energy use and anaesthetic gas and MDI use, did not have reported error values. For the MRIO-based results, previous work can provide context: an uncertainty analysis done for an earlier version of the UK MRIO model found that total UK consumer greenhouse gas emissions carried a relative SE of approximately 4–6%; previous health-care footprinting analyses found an SD of less than 5% globally and relative SEs of approximately 15% for China and 28% for Australia.^5^, ^6^, ^30^ Qualitative sources of uncertainty are elaborated on in the Discussion.

### Role of the funding source

The funder of the study had no role in study design, data collection, data analysis, data interpretation, writing of the report, or the decision to submit the paper for publication. All authors had full access to all the data in the study and had final responsibility for the decision to submit for publication.

## Results

**Figure 2:**
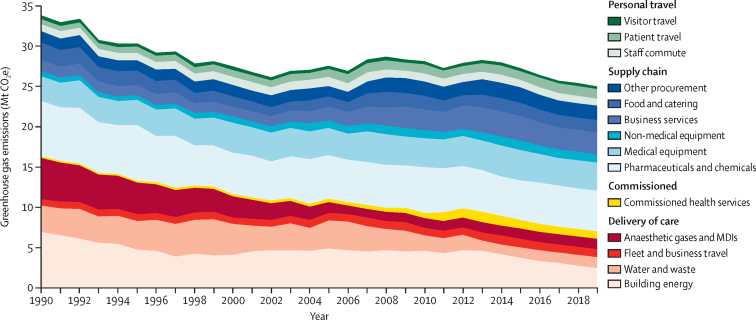
Time series results for the greenhouse gas emissions of the NHS in England, broken down by source of emission, 1990–2019 Data available in appendix 1 (p 39). MDI=metered dose inhaler. Mt CO_2_e=megatonnes of carbon dioxide equivalent. NHS=National Health Service.

**Figure 3:**
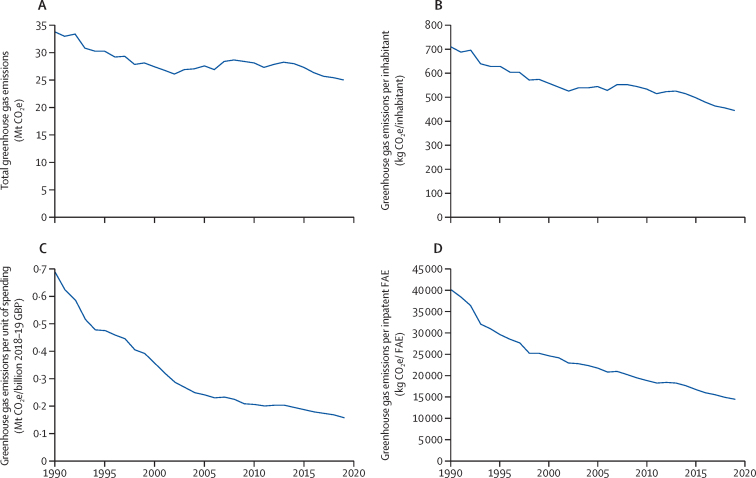
Greenhouse gas emissions for the NHS in England, presented as total emissions (A), emissions per inhabitant (B), emissions per real unit of spending (C), and emissions per inpatient FAE (D) Data available in appendix 1 (pp 40–41). Real spending is calculated in terms of GBP for the 2018–19 financial year. FAE=finished admission episode. GBP=British pound. Mt CO_2_e=megatonnes of carbon dioxide equivalent. NHS=National Health Service.

For 2019, the most recent year of our analysis, the carbon footprint for the NHS in England was estimated at 25 megatonnes (Mt) of CO_2_e, representing a decrease of approximately 26% from 1990 (figure 2; appendix 1 p 39). A substantial contributor to this reduction has been the decarbonisation of the energy system, which has contributed to the 64% reduction in building energy from 1990 to 2019 observed in figure 2 and was the main driver of a 43–45% decrease in total national greenhouse gas emissions over the same time period.^31^, ^32^ At the same time, the population of England increased by 17%; the provision of care doubled, measured as the number of inpatient FAEs; and health spending more than tripled in real terms. This translated to a reduction in the carbon intensity of the NHS of 37% for CO_2_e per capita and 64% per inpatient FAE between 1990 and 2019 (figure 3; appendix 1 pp 40–41).

**Figure 4:**
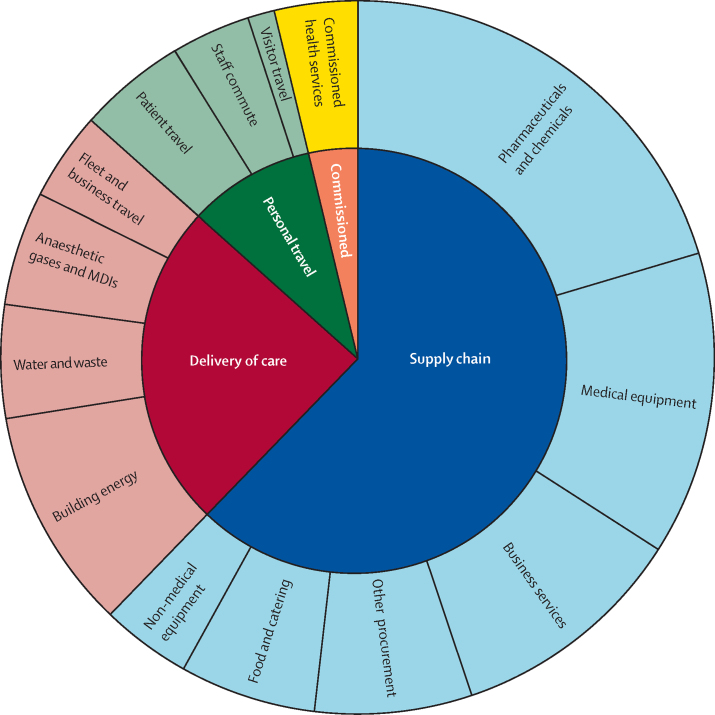
Contribution of different sectors to the greenhouse gas emissions of the NHS England, 2019 Data available in appendix 1 (p 39). MDI=metered dose inhaler. NHS=National Health Service.

In 2019, the largest share of emissions by far were from the supply chain (62% [15·6 Mt CO_2_e]), followed by delivery of care (24% [6·1 Mt CO_2_e]), and travel to and from NHS sites by patients and visitors and staff commuting (10% [2·4 Mt CO_2_e]; figure 4; appendix 1 p 39). Private health and care services commissioned by the NHS contributed the final 4% (1·0 Mt CO_2_e). Supply chain emissions were dominated by the manufacturing of goods such as pharmaceuticals and chemicals (32% [5·1 Mt CO_2_e]), and medical equipment (19% [3·0 Mt CO_2_e]); however, emissions associated with the provision of business services (17% [2·7 Mt CO_2_e]) such as indemnity insurance also comprised an important component (figure 4; appendix 1 p 39). The construction of health-care facilities and freight transport of goods, activities often highlighted in sustainability efforts, contributed to only 5% (0·8 Mt CO_2_e) and 6% (1·0 Mt CO_2_e) of supply chain emissions, respectively.

Overall, NHS England's greenhouse gas emissions have gone through three distinct phases since 1990 (figure 2; appendix 1 p 39). In the first phase, from 1990 to 2000, emissions fell steeply from 33·8 Mt CO_2_e to 27·5 Mt CO_2_e, driven largely by the phase-out of chlorofluorocarbon propellants in inhalers in compliance with the Montreal Protocol,^33^ reduced reliance on coal and oil for onsite heating, and by reductions in supply chain emissions from pharmaceuticals, chemicals, and gases as technologies improved. In the second phase, from 2001 to 2012, overall emissions grew again, reaching 27·8 Mt CO_2_e, driven by investment in health care from 4·7% to 7·6% of gross domestic product in 2009, and subsequent increased supply chain emissions, from 13·3 Mt CO_2_e in 2001 to 15·7 Mt CO_2_e in 2012. Emissions from commissioned health-care activities approximately tripled also over this period, from 0·3 Mt CO_2_e to 1·1 Mt CO_2_e. In the third phase, from 2013 to 2019, overall emissions fell again, led by reductions in emissions from electricity generation as the UK grid has rapidly decarbonised.

When considering emissions based on clinical activity for 2019, it is clear that, unsurprisingly, health care provided by acute services takes place in the most resource-intensive clinical settings and is the largest contributor overall, comprising more than half (56%) of total emissions (table). Primary care contributions derive primarily from the supply chain and prescriptions of MDIs. When considered by unit of activity, this results in an estimated carbon footprint of 125 kg CO_2_e per bed-day and 76 kg CO_2_e per outpatient appointment for acute care, 66 kg CO_2_e per general practice visit, and 75 kg CO_2_e per ambulance emergency response, among others.^29^

**Table:** Clinical activity-based greenhouse gas emissions by emissions category, 2019

		**Ambulance**	**Community**	**Mental health**	**Acute**	**Primary care**	**Non-clinical support activities**	**Total**
Delivery of care								
	Building energy	21	150	164	1900	250	31	2520
	Anaesthetic gases and metered dose inhalers	84	0	0	435	767	0	1290
	Water and waste	16	85	95	883	137	88	1300
	Business travel and fleet	200	100	120	410	60	110	1000
Supply chain								
	Pharmaceuticals and chemicals	5	120	66	2095	2750	26	5060
	Medical equipment	16	147	55	1930	248	128	2520
	Non-medical equipment	38	156	170	1040	420	137	1960
	Other procurement	100	384	465	2850	610	1620	6030
Commissioned services		3	15	26	90	0	826	960
Personal travel		27	120	350	1326	536	43	2400
Total		510	1280	1510	12 960	5770	3010	25 040

Data are kilotonnes of carbon dioxide equivalent. Totals might vary due to rounding.

## Discussion

Carbon footprint results for the NHS in England reveal the most influential sources of emissions throughout the health-care system, and how each has changed over the past three decades, allowing the foundation of strategic planning going forwards. Enhanced efforts for national and global trends will also be key to achieve emissions reductions goals. The NHS's 2019 Long-Term Plan describes a wide variety of commitments to the digital redesign of health care, improvements in technology, and renewed climate change mitigation ambitions.^34^ Although the NHS can influence all emissions sources, its ability to effect changes varies considerably.

The NHS has the most direct operational control over its delivery of care emissions (figure 1); this source category made up fully a quarter of overall emissions in 2019. Numerous interventions are already being implemented across NHS estates and facilities, with others targeted in its strategic planning. Engineering and sociotechnical interventions are staged first and include widespread insulation and upgrading of building envelopes, efficient appliances, and more sophisticated control systems, which could reduce energy use by an estimated 40%. The next step, which will need to overlap in time with the energy reduction interventions, is to convert gas boilers to electric heat pumps, thereby improving efficiency and switching to a lower-carbon energy source. Once buildings are fully electrified (with appropriate technology to provide backup systems), grid decarbonisation will deliver emissions reductions. The carbon intensity of UK grid electricity is projected to fall from current levels of 288 kg CO_2_e/MWh to 27 kg CO_2_e/MWh in 2050, and potentially to zero under the UK Government's commitment to reach Net Zero by 2050.^35^ NHS facilities could decarbonise more rapidly by installing on-site solar photovoltaics or wind generation. The earlier this occurs, the greater the emissions benefit will be relative to grid electricity, which will decline over time. Considering other sources within delivery of care, emissions from water, wastewater, and solid waste can be reduced through water efficiency projects and waste reduction measures, including reducing the use of single-use consumables.

While many of the interventions above focus on health-care facility operations, optimising how clinical care is delivered is at the heart of the NHS mission and is a major focus of sustainability efforts, led by clinicians and staff. Focusing on in-use emissions from pharmaceuticals, emissions of waste anaesthetic gases can be addressed through the reduction of fresh gas flows and low-carbon drug selection by clinicians; destruction technologies are also available for nitrous oxide, while the development of capture technologies for volatile anaesthetics shows promise for the near future. Interventions to mitigate MDI emissions include conversion to dry powder inhalers (with no gas propellant) when feasible, expanding patient education efforts to maximise correct usage, and incentivising pharmacy take-back programmes to safely dispose of partially spent MDIs.^36^ Although only a small component of health-care activity, emissions from anaesthetic gases and MDIs can make up a substantial proportion of health-care emissions and should be considered with a dedicated stream of analysis.

Importantly, health promotion and disease prevention programmes such as public health campaigns and social prescribing can reduce the overall demand for health care, while the selection of a less carbon-intensive and resource-intensive care practices where clinically appropriate can reduce both emissions and costs. The activity-based emissions results could be used to set priorities in disease prevention, as well as development and adoption of lower carbon best practices.

Supply chain emissions are the largest source category but are the most difficult to influence directly. Two important general strategies are reducing the overall demand for goods and services (eg, through reducing drug wastage or refurbishing and reusing devices) and shifting to low-carbon goods and services (eg, through specifying so-called green concrete, low-carbon data services, or plant-based diets). The NHS has produced several tools to guide green procurement, including footprinting guidance and reports identifying the most carbon-intensive purchased items and pharmaceuticals.^37^ The last option is to reduce the overall emissions intensity of manufactured goods and services, which will include emissions taking place in the UK and foreign countries. In many countries, reductions are already occurring by virtue of energy system decarbonisation and the upgrading of industrial equipment. The NHS can incentivise further and faster progress through partnerships with tier 1 suppliers that pledge aggressive emissions reduction targets.

Finally, for transport, the most impactful interventions will be to promote active travel and public transit to and from NHS sites, as well as the phasing in of electric ambulances and other NHS vehicles. These interventions will also benefit health through reduced local air pollution, reducing vehicular accidents, and increasing physical activity.^38^, ^39^ Shifting modes of care can also avoid travel for in-person visits, including telehealth monitoring of long-term conditions, remote diagnostics, and virtual appointments, with particular health benefits to those with reduced mobility, and reducing exposure of patients to hospital environments and possible health-care-acquired infections.

Per-capita results for the NHS in England (plus social care and public health) of 540 kg CO_2_e per capita compared with similar national studies of health-care sectors place it in proximity to results for Japan (566 kg CO_2_e per capita in 2015) but less than those for Austria (799 kg CO_2_ per capita in 2014, CO_2_ only), Canada (899 kg CO_2_e per capita in 2015), Australia (1,495 kg CO_2_e per capita in 2015), and the USA (1889 kg CO_2_e per capita in 2013).^5^, ^7^, ^9^, ^10^, ^12^ Among previous national studies, only Nansai and colleagues^7^ for Japan have provided results by activity (and also by disease type and age cohort), and only Weisz and colleagues^9^ for Austria have made use of bottom-up data. Other international studies have evaluated the UK health-care carbon footprint; these use aggregated health expenditure data and environmentally extended MRIO models without bottom-up data and are not directly comparable to the results presented here. On a per-capita basis, Pichler and colleagues^11^ estimated 640 kg CO_2_ per capita (CO_2_ only) in 2014. International input–output-based estimates can differ in magnitude from bottom-up estimates because they more completely capture upstream emissions.

Our analysis represents the most detailed national health-care carbon footprinting effort to date, enabled by a wealth of bottom-up data and the use of the sophisticated UK MRIO model, which combines the high sectoral resolution of UK input–output tables with the comprehensive representation of international economic structures and emission intensities of an MRIO model. Moving forwards, as the NHS continues to track its progress, further data improvements will become essential to reflect progress on emissions reductions. For this, the vintage of individual datasets and models is an important consideration. For example, input–output tables for the UK are published with a 4-year lag (current latest information is for 2016) and so carbon intensities and expenditure apportionments must be carefully projected to reflect current conditions. For some emissions categories, data are only available for a subset of years and are currently extrapolated (appendix 1 pp 2–3, 43–45). These projections contribute to the uncertainty of the results, as do other factors including the blending of bottom-up and top-down analyses in a hybrid model, the matching of supply chain sectors to categories in the MRIO concordance, and the estimation of missing bottom-up data where completeness of the MRIO model cannot be used. Improved data monitoring and collection will provide more robust and high-resolution results that can be used at the individual facility level—eg, using financial and vehicle audit data to create bottom-up business transport datasets rather than using top-down modelling. Finally, including emissions of hydrofluorocarbons used as refrigerants as well as of sulphur hexafluoride used in some eye surgeries will ensure that the complete set of Scope 1 greenhouse gases is being included.

In conclusion, as health systems respond to the increasing health impacts of climate change on global populations, they both contribute to the problem, generating 4–5% of global greenhouse gas emissions, and have a central role to play in the solution.^1^ Acknowledging this, the NHS has put more than a decade's worth of work in measuring and monitoring its greenhouse gas emissions, with the results recorded in this Article. This represents the first and imperative step to enable the identification and subsequent mitigation of greenhouse gas emissions, with sustained investment required for the analysis to be useful for long-term planning and strategic decision making. In assessing the applicability of these methods to health systems in other countries, a number of key features are apparent. First, adopting a hybrid model provides flexibility and balances the need for a comprehensive assessment with the availability of bottom-up data. Countries have varying levels of coverage and quality in bottom-up data, which should be used and improved where possible, as these can be used to monitor and support local actions. Second, any bottom-up data should be regularly updated and validated to ensure that results are timely and reflect changes in health-care investments, demographics, and emissions intensities. Finally, it is important that any mitigation efforts actively seek to generate health co-benefits, reduce environmental impacts, and maintain or improve quality of care. Only then will health systems comply with their duty to first, do no harm.

## Data sharing

Data for this work are available in appendix 1, and in machine-readable format in appendix 2.
