# Supplementary material for: Health care's response to climate change: a carbon footprint assessment of the NHS in England
Source: Lancet Planet Health. 2021 Feb 10;5(2):e84–92. doi: 10.1016/S2542-5196(20)30271-0 (PMC7887664; doi:10.1016/S2542-5196(20)30271-0)
Supplement: Supplementary appendix 1 [file mmc1.pdf]

THE LANCET
Planetary Health

Supplementary appendix

This appendix formed part of the original submission and has been peer reviewed. We post it as supplied by the authors.

Supplement to: Tennison I, Roschnik S, Ashby B, et al. Health care's response to climate change: a carbon footprint assessment of the NHS in England. *Lancet Planet Health* 2021; **5:** e84–92.

Healthcare's response to climate change: The carbon footprint of the NHS in England

Supplementary Appendix

Table S1. Data sources and modeling approach by emissions source category and basis for time series projections and backasts 2

Table S2. ERIC data definitions 4

Table S3. Estimates for excluded building categories from ERIC (2015) 7

Table S4. Emission factors for fuels and electricity 8

Table S5. NHS in England expenditures, allocated by UK-MRIO model sectors, £ million 9

Table S6. UK-MRIO model GHG emission factors, CO_2e tonnes / £ thousand**s** 21

Table S7. UK-MRIO concordance to NHS in England supply chain emissions categories 32

Table S8. Summary of accounting for transport-related emissions 35

Table S9. Average commuter trip distance of health professionals by employment status and region of workplace, 3-year averages, 2011-2017, calculated by the Department for Transport from Table NTS0411 36

Table S10. Average distance travelled by main mode for selected (health) trip purposes, England, 2002-2016, calculated by Department for Transport from Table NTS0305 37

Table S11. Travel emission intensities, kg CO_2e/km 38

Table S12. NHS in England Carbon Footprint by Source Contribution, 1990-2019, in Mt CO_2e 39

Table S13. NHS in England carbon footprint per inhabitant, real unit of spend, and inpatient Finished Admission Episode (FAE) 40

Section S1. GHG emissions accounting methods description 42

Section S2. Forecasting / Backcasting methods description 43

References 46

Table S1. Data sources and modelling approach by emissions source category and basis for time series projections and backcasts

Emissions Source Category	Modeling approach	Data Source	Actual data	Backcast years	Projection years	Basis for backcast and projections
Building energy use – hospitals	Bottom up	ERIC	1990 - 2018	n/a	2019	Continuing trends
Building energy use – other sites (GP, offices)	Bottom up	Sample data source	2014 – 2015	1990 – 2013	2016 – 2019	Backcast based on hospital energy use;<br><br>Forecast based on expenditures adjusted for inflation
Inhaled Anaesthetics - volatiles	Bottom up	Supplier information	2016 – 2019	1990 – 2015	n/a	Backcast based on bed days
Inhaled Anaesthetics – nitrous oxide	Bottom up	Supplier information	2011	1990 – 2010	2012 – 2019	Surgical nitrous oxide: backcast and forecast using bed days<br><br>Maternity nitrous oxide: backcast an forecast based on the number of maternities<br><br>Emergency nitrous oxide: backcast and projection based on A&E attendances<br><br>Ambulance nitrous oxide: backcast and projection based on ambulance calls that receive face-to-face response from the ambulance service
Meter Dose Inhalers HFCs	Bottom up	NAEI	2006 – 2017	1990 – 2005	2017 – 2019	Backcast using linear increase from introduction of HFC inhalers in 1997
Meter Dose Inhalers CFCs	Bottom up	NAEI	1990	n/a	1991 – 2019	Linear reduction 1990 to 2006; 0 afterwards
Water, Wastewater, and Solid Waste	Top down	UK MRIO	1997 – 2016	1990 – 1996	2017 – 2019	Expenditures adjusted for inflation
Electricity factors	Bottom up	BEIS<br><br>Green Book	2002 – 2017<br><br>2018 – 2019	1990 - 2002	n/a	n/a
Personal travel – staff commuting, visitor, patient	Top down	NTS	2002 – 2018	1990 – 2001	2019	Continuing trends
Fleet and business travel	Top down	UK MRIO	1997 – 2016	1990 – 1996	2017 – 2019	Expenditures adjusted for inflation
Supply Chain	Top down	UK MRIO	1997 – 2016	1990 – 1996	2017 – 2019	Expenditures adjusted for inflation

Emissions Source Category	Modeling approach	Data Source	Actual data	Backcast years	Projection years	Basis for backcast and projections
Commissioned	Top down	UK MRIO	1997 – 2016	1990 – 1996	2017 – 2019	Expenditures adjusted for inflation

Table S2. ERIC data definitions

**Total electricity consumed for a site** = Electricity consumed (S06_02) + Electricity consumed - green energy tariff (S06_10) + Electricity consumed - third party owned renewable (S06_12) + Electrical energy output of CHP system(s) (S05_06) - Exported electricity (S05_07)			
**Scope 2 energy for a site** = Electricity consumed (S06_02) + Electricity consumed - green energy tariff (S06_10) + Electricity consumed - third party owned renewable (S06_12) - Exported electricity (S05_07) + Steam consumed (S06_16) + Hot water consumed (S06_17) - Exported thermal energy (S05_08)			
**Ref**	**Field**	**Unit**	**Definition**
S06_02	Electricity consumed	kWh	The total amount of electricity consumed from the national/regional/local electricity supplier. Include energy used to feed CHP plant associated with the site and energy used by the organisation site for processing purposes (e.g. laundry, CSSD). In cases where the organisation site includes an on-site central processing unit(s) which serves more than one organisation site, the input energy to this unit should be apportioned in accordance with the percentage output used by the organisation for its own purposes (e.g. if the organisation uses 30% of the processing unit output then the figure included should be 30% of the total amount of energy supplied to the processing unit). Exclude energy derived from an eligible renewable energy source.
S06_04	Gas consumed	kWh	The total amount of gas consumed from the national /regional gas supplier, including LPG. Include energy used to feed CHP plant associated with the site, and energy used by the organisation site for processing purposes (e.g. laundry, CSSD). In cases where the organisation site includes an on-site central processing unit(s) which serves more than one organisation site, the input energy to this unit should be apportioned in accordance with the percentage output used by the organisation for its own purposes (e.g. if the organisation uses 30% of the processing unit output then the figure included should be 30% of the total amount of energy supplied to the processing unit).
S06_06	Oil consumed	kWh	The total amount of oil consumed from the national /regional oil supplier. Consumption should be adjusted for accrued liabilities. Include energy used to feed CHP plant associated with the site, and energy used by the organisation site for processing purposes (e.g. laundry, CSSD). In cases where the organisation site includes an on-site central processing unit(s) which serves more than one organisation site, the input energy to this unit should be apportioned in accordance with the percentage output used by the organisation for its own purposes. (For example, if the organisation uses 30% of the processing unit output, then the figure included should be 30% of the total amount of energy supplied to the processing unit).

S06_08	Coal consumed	kWh	The total amount of coal consumed from the national /regional coal supplier. Include energy used to feed any CHP plant associated with the site, and energy used by the organisation site for processing purposes (e.g. laundry, CSSD). In cases where the organisation site includes (an) on-site central processing unit(s) which serves more than one organisation site, the input energy to this unit should be apportioned in accordance with the percentage output used by the organisation for its own purposes (e.g. if the organisation uses 30% of the processing unit output then the figure included should be 30% of the total amount of energy supplied to the processing unit).
S06_10	Electricity consumed - green energy tariff	kWh	The total annual amount of electricity from renewable or green energy tariffs supplied by national/regional or local electricity supplier e.g. Crown Commercial Services “green energy”. If a proportion supplied electricity is from a green energy tariff then only the proportion which is a ‘green energy tariff’ should be reported here. The sum of the electricity consumed, green energy tariff and third party owned renewable will be the total 'intake' of electrical energy delivered to the site.
S06_12	Electricity consumed - third party owned renewable	kWh	The total annual amount of electricity consumed from renewable sources owned and supplied by national/regional or local electricity supplier but not supplied through the national power grid e.g. onsite renewable rent-a-roof schemes, community owned/funded renewable energy projects or supplies bought via a private wire from a renewable supply. Consumption should be adjusted for accrued liabilities. Exclude energy consumed via green energy tariffs and organisation owned renewable generation. Include energy used by the organisation site for processing purposes (e.g. laundry, CSSD). In cases where the organisation site includes (an) on-site central processing unit(s) which serves more than one organisation site, the input energy to this unit should be apportioned in accordance with the percentage output used by the organisation for its own purposes. (For example, if the organisation uses 30% of the processing unit output, then the figure included should be 30% of the total amount of energy supplied to the processing unit). Note: Please report all third party owned and operated renewable electricity consumed here.
S06_14	Non-fossil fuel consumed - renewable	kWh	The total annual amount of non-fossil fuel from renewable energy consumed for combustion (e.g. heating boilers) which has been supplied from an eligible renewable energy supply source as defined in Protocol A3 of DEFRA "Guidelines for the Measurement and Reporting of Emissions by Direct Participants in the UK Emissions Trading Scheme" (e.g. wind energy, hydro power (up to 20MW output), tidal power, wave energy, photovoltaics (solar panels), photoconversion, geothermal hot dry rock, geothermal aquifers, municipal and industrial wastes, landfill gas, agricultural and forestry wastes, sewerage gas, biomass, etc). Such energy to be exempt from the Climate Change Levy (CCL), which is evidenced by a Levy Exemption Certificate that confirms that electricity generated from qualifying renewable sources are exempt from the Levy. Please see the 'Guidelines for the Measurement and Reporting of Emissions by Direct Participants in the UK Emissions Trading Scheme' guidance document, which is available from the 'Downloads' section in the ERIC module of the Estates and Facilities Management (EFM) system.
S06_16	Steam consumed	kWh	Total annual amount of steam consumed which has been supplied by an organisation other than regional/national supplier sources (e.g. a neighbouring organisation supplying steam to the site from their incinerator or central boiler plant and distribution system). Exclude energy derived from an eligible renewable energy source.
S06_17	Hot water consumed	kWh	Total annual amount of hot water consumed which has been supplied by an organisation other than regional/national supplier sources (e.g. a neighbouring organisation supplying hot water to the site from their main distribution system.) Exclude energy derived from an eligible renewable energy source.
S06_18	Electrical energy output of owned onsite renewables	kWh	The total annual amount of electricity output from owned onsite low or zero carbon renewable generation, inclusive of exported electricity. Excludes electrical energy output of CHP recorded separately. The sum of the electrical output of CHP and onsite renewable electrical energy will be the total 'generated' onsite electrical energy.
S05_05	Thermal energy output of CHP system(s)	kWh	The total useful thermal energy output, inclusive of exported thermal energy.
S05_06	Electrical energy output of CHP system(s)	kWh	The total electrical energy output, inclusive of exported electricity.

S05_07	Exported electricity of CHP system(s)	kWh	The amount of surplus electricity in kWh produced by the trust site from CHP plant which is supplied to a utility supplier or another organisation site. Please note that the imported energy provided to other trusts completing an ERIC return should include this in their electricity consumed field.
S05_08	Exported thermal energy of CHP system(s)	kWh	The amount of surplus thermal energy in kWh produced by the organisation site from CHP plant which is supplied to a utility supplier or another organisation site. Please note that the imported energy provided to other trusts completing an ERIC return should include this in the relevant energy field.

Table S3. Estimates for excluded building categories from ERIC (2015)

Healthcare building type	Energy Use (GWh)			GHG Emissions (ktCO2e)				Source
	**Electricity**	**Gas**	**Oil**	**Electricity**	**Gas**	**Oil**	**Total**	
General Practice (GP)	305	455	0	189	94	0	283	Sheffield GP survey
Dentists	79	221	0	49	46	0	94	Independent research [1]
Non-clinical support services - Clinical Commissioning Groups	21	13	0	13	3	0	16	Sustainability reporting [2]
Non-clinical support services - Central and regional offices	45	40	6	28	8	2	38	Annual reporting [3]
Secondary care - under-reporting in ERIC	163	668	0	101	138	0	239	
**Total**	**614**	**1,397**	**6**	**380**	**289**	**2**	**671**	

[1]. Private correspondence following: Duane B, Lee MB, White S, Stancliffe R, Steinbach I. An estimated carbon footprint of NHS primary dental care within England. How can dentistry be more environmentally sustainable? *British Dental Journal* 2017; 223(8): 589-93.

[2]. Reporting covers 8.4% of CCG expenditures, electricity and natural gas consumption extrapolated to England total based on this ratio.

[3]. Extracted from: Department of Health. Annual Report and Accounts 2014-15 London: Government of the United Kingdom, 2015.

Table S4. Emission factors for fuels and electricity

Results year(s)	Electricity use year	Electricity emissions intensity reporting year	Electricity emissions intensity source
2020-2050	Forecast	2020-2050	Green Book
2019	2019/20	Average of:	Company reporting
		2019 and 2020	Green Book
2018	2018/19	2020	Company reporting
2017	2017/18	2019	Company reporting
2016	2016/17	2018	Company reporting
2002-2015	2002-2015	2004-2017	Company reporting
2001	2001/02	2003	Company reporting
2000	2000/01	2002	Company reporting
1990-1999	1990-1999	1990-1999	2011 Company reporting (multiple years)

Table S5. NHS in England expenditures, allocated by UK-MRIO model sectors, £ million

Sector – UK	1997	1998	1999	2000	2001	2002	2003	2004	2005	2006	2007	2008	2009	2010	2011	2012	2013	2014	2015	2016
Products of agriculture, hunting and related services	31.1	27.8	27.7	27.1	21.9	21.9	21.2	25.9	22.0	20.3	21.9	25.8	27.2	26.1	30.3	21.8	26.1	31.7	26.7	28.5
Products of forestry, logging and related services	-	-	-	-	-	-	-	-	-	-	-	-	-	-	-	-	-	-	-	-
Fish and other fishing products; aquaculture products; support services to fishing	-	-	-	-	-	-	-	-	-	-	-	-	-	-	-	-	-	-	-	-
Coal and lignite	-	-	-	-	-	-	-	-	-	-	-	-	-	-	-	-	-	-	-	-
Crude Petroleum And Natural Gas & Metal Ores	-	-	-	-	-	-	-	-	-	-	-	-	-	-	-	-	-	-	-	-
Other mining and quarrying products	-	-	-	-	-	-	-	-	-	-	-	-	-	-	-	-	-	-	-	-
Mining support services	-	-	-	-	-	-	-	-	-	-	-	-	-	-	-	-	-	-	-	-
Preserved meat and meat products	140.6	119.4	130.6	129.1	114.7	117.3	102.3	208.7	190.5	146.9	162.6	203.8	211.8	201.3	191.0	201.3	223.7	234.0	247.5	251.0
Processed and preserved fish, crustaceans, molluscs, fruit and vegetables	58.8	54.0	60.5	58.6	55.9	54.1	53.4	60.9	49.0	42.6	45.0	102.1	119.9	135.3	138.5	137.0	150.6	144.7	155.9	164.9
Vegetable and animal oils and fats	11.6	11.6	15.0	15.4	18.6	21.6	21.4	27.3	25.7	15.2	9.9	14.7	17.7	19.6	16.7	12.7	16.1	21.9	21.8	26.3
Dairy products	81.1	75.3	66.8	66.9	62.4	59.9	56.1	52.4	40.3	29.3	33.3	36.7	43.7	44.5	45.4	44.4	50.0	50.3	52.8	56.8
Grain mill products, starches and starch products	16.9	19.6	22.2	22.8	24.1	25.6	25.2	31.6	18.9	17.6	18.8	21.1	25.7	25.5	26.8	27.0	28.1	31.1	32.0	33.3
Bakery and farinaceous products	42.8	40.8	45.6	43.1	39.4	37.1	31.3	56.0	47.7	37.6	43.7	55.1	62.9	63.5	63.7	64.5	69.5	72.5	73.5	77.7
Other food products	90.7	88.9	100.6	109.1	115.4	116.3	103.7	143.3	115.4	97.7	112.4	139.3	160.1	159.6	165.6	171.6	211.0	225.0	237.9	249.8
Prepared animal feeds	-	-	-	-	-	-	-	-	-	-	-	-	-	-	-	-	-	-	-	-
Alcoholic beverages	1.9	2.1	2.0	3.4	2.0	2.3	2.5	4.0	2.2	-	-	-	-	-	-	-	-	-	-	1.9
Soft drinks	12.7	12.2	9.0	11.6	9.2	9.6	6.0	4.7	7.4	2.3	2.4	1.7	4.2	3.2	2.4	1.4	1.4	3.1	3.4	5.1
Tobacco products	0.2	0.1	-	0.2	0.2	0.3	-	-	-	-	-	-	-	-	-	-	-	-	-	-
Textiles	51.7	53.2	58.2	64.4	61.3	65.7	66.6	77.6	83.4	70.6	76.2	88.7	94.9	80.3	71.8	73.2	76.3	87.5	90.6	95.5
Wearing apparel	79.0	67.5	68.4	66.1	57.0	45.6	37.1	42.4	32.6	26.9	31.8	32.9	29.3	68.4	22.1	13.3	-	1.8	2.4	2.1
Leather and related products	7.0	5.4	3.7	4.0	2.9	2.3	1.4	1.0	0.4	0.4	0.6	0.6	2.3	1.6	4.4	2.2	-	6.8	8.0	14.6
Wood and of products of wood and cork, except furniture; articles of straw and plaiting materials	4.5	3.9	3.5	3.7	3.6	4.1	4.5	2.8	-	2.9	2.1	1.6	0.6	-	1.1	1.8	2.5	3.8	3.8	4.3
Paper and paper products	158.9	190.1	217.4	231.2	213.3	236.8	209.2	256.8	225.4	199.9	222.3	274.0	316.5	307.5	331.9	382.5	474.2	534.3	543.7	552.5
Printing and recording services	182.5	200.1	234.1	263.6	258.7	247.6	234.8	299.5	301.1	303.9	367.8	455.5	517.2	542.1	538.0	553.7	643.8	709.9	748.1	779.2
Coke and refined petroleum products	43.1	36.7	48.1	61.3	68.0	80.7	86.1	88.5	76.9	78.4	58.9	54.0	55.6	59.8	63.8	68.1	65.9	68.3	71.3	62.3
Paints, varnishes and similar coatings, printing ink and mastics	6.1	6.0	5.2	7.8	6.0	6.8	6.8	6.6	4.3	3.0	-	-	-	-	0.1	0.2	0.2	0.8	0.7	1.4
Soap and detergents, cleaning and polishing preparations, perfumes and toilet preparations	148.6	154.4	176.3	218.8	247.6	279.8	300.4	85.3	63.5	16.7	8.5	8.8	8.6	7.6	5.3	2.9	-	2.9	3.1	3.7
Other chemical products	88.6	97.5	149.0	179.6	212.1	257.9	284.8	565.8	581.6	338.1	262.3	179.2	169.4	114.4	143.6	173.7	211.0	260.0	286.3	301.2
Industrial gases, inorganics and fertilisers (all inorganic chemicals) - 20.11/13/15	33.8	36.8	49.2	52.1	66.0	83.3	83.8	134.5	149.1	129.5	137.3	143.2	120.9	145.9	73.4	44.7	-	144.0	158.1	167.4
Petrochemicals - 20.14/16/17/60	20.0	20.4	22.4	33.1	39.3	44.0	36.6	36.9	24.8	10.0	-	-	-	-	3.0	2.8	3.3	5.4	6.0	16.9
Dyestuffs, agro-chemicals - 20.12/20	41.0	45.1	56.6	68.0	74.8	79.6	113.1	108.1	160.4	134.8	88.8	51.3	31.3	0.6	0.4	0.2	-	5.3	5.8	6.4
Basic pharmaceutical products and pharmaceutical preparations	156.7	143.5	126.4	123.3	156.9	151.0	130.3	82.8	152.6	245.4	370.5	716.3	1,086.6	1,391.1	1,363.9	1,360.6	1,540.4	1,028.8	925.9	929.2
Rubber and plastic products	54.6	76.1	89.9	102.3	108.4	120.4	128.2	174.3	182.0	162.8	175.5	214.5	252.0	218.7	211.2	227.5	253.2	290.6	299.7	299.0
Manufacture of cement, lime, plaster and articles of concrete, cement and plaster	19.1	18.6	24.5	27.8	28.9	31.5	30.8	36.3	34.9	32.3	29.7	37.2	38.7	37.3	36.2	35.4	39.1	43.0	41.5	43.2
Glass, refractory, clay, other porcelain and ceramic, stone and abrasive products - 23.1-4/7-9	13.8	14.0	20.3	23.8	19.6	23.1	20.4	27.9	21.2	15.1	15.8	17.9	18.1	16.6	15.8	17.5	18.9	17.2	18.1	18.4
Basic iron and steel	1.9	2.0	2.3	0.8	0.7	0.8	-	-	-	-	-	-	-	-	-	-	-	-	-	-
Other basic metals and casting	1.1	1.2	0.5	0.5	0.5	0.6	-	-	-	-	-	-	-	-	-	-	-	-	-	-

Sector – UK	1997	1998	1999	2000	2001	2002	2003	2004	2005	2006	2007	2008	2009	2010	2011	2012	2013	2014	2015	2016
Weapons and ammunition	-	-	-	-	-	-	-	-	-	-	-	-	-	-	-	-	-	-	-	-
Fabricated metal products, excl. machinery and equipment and weapons & ammunition - 25.1-3/25.5-9	24.1	25.2	33.8	36.4	36.4	40.1	37.6	49.8	43.5	36.6	41.5	51.9	58.3	59.2	59.2	63.1	67.2	68.4	71.4	70.0
Computer, electronic and optical products	1,542.0	1,615.2	2,050.9	2,046.1	1,981.8	2,317.9	1,966.8	1,630.2	1,673.3	1,193.9	1,057.6	923.3	902.2	922.7	940.2	958.9	890.1	885.1	841.8	813.9
Electrical equipment	11.6	11.6	12.9	11.2	13.4	15.7	16.3	15.7	12.7	2.5	1.8	1.2	0.5	-	0.9	1.4	6.7	16.1	18.7	23.1
Machinery and equipment n.e.c.	1.3	1.5	2.3	2.6	2.5	2.8	1.9	-	3.6	-	2.3	2.1	1.7	2.3	2.2	0.8	-	1.2	1.2	1.6
Motor vehicles, trailers and semi-trailers	16.5	15.3	14.7	17.4	19.1	19.3	18.6	20.3	34.6	32.6	26.9	15.9	14.0	14.3	14.5	14.7	13.6	13.5	13.5	13.2
Ships and boats	-	-	-	-	-	-	-	-	-	-	-	-	-	-	-	-	-	-	-	-
Air and spacecraft and related machinery	-	-	-	-	-	-	-	-	-	-	-	-	-	-	-	-	-	-	-	-
Other transport equipment - 30.2/4/9	28.7	41.0	55.5	60.7	63.4	70.8	75.5	98.7	98.2	87.6	92.1	83.8	79.2	70.7	81.7	76.8	85.5	84.1	89.9	80.3
Furniture	47.0	52.3	62.6	60.8	63.5	58.4	44.8	69.6	60.9	59.4	71.5	73.0	84.6	86.2	74.7	71.6	59.2	67.6	81.1	82.0
Other manufactured goods	16.4	15.3	13.6	13.2	14.6	12.8	16.2	14.4	11.9	10.0	7.3	4.8	4.1	2.3	1.6	0.8	-	1.4	1.6	2.0
Repair and maintenance of ships and boats	-	-	-	-	-	-	-	-	-	-	-	-	-	-	-	-	-	-	-	-
Repair and maintenance of aircraft and spacecraft	-	-	-	-	-	-	-	-	-	-	-	-	-	-	-	-	-	-	-	-
Rest of repair; Installation - 33.11-14/17/19/20	6.9	7.4	7.0	10.2	5.7	9.5	10.5	12.4	7.8	4.8	4.1	3.7	2.4	2.8	4.6	3.5	5.3	6.9	7.8	9.4
Electricity, transmission and distribution	296.0	305.8	338.3	331.1	293.2	263.2	269.2	331.5	374.8	430.7	507.8	605.6	677.3	717.3	729.6	728.9	791.7	777.8	801.7	842.5
Gas; distribution of gaseous fuels through mains; steam and air conditioning supply	111.1	113.9	126.3	140.8	170.2	168.5	156.0	199.8	298.5	270.7	311.6	385.1	433.5	405.9	490.8	483.8	486.6	557.4	566.8	588.5
Natural water; water treatment and supply services	42.0	43.4	40.0	40.4	39.1	39.4	35.8	33.5	28.0	23.8	26.9	35.2	40.6	36.9	33.5	44.3	74.8	81.1	102.9	114.5
Sewerage services; sewage sludge	153.5	163.6	206.0	187.0	178.2	175.8	233.0	231.4	280.8	324.0	363.8	394.5	440.3	399.5	442.0	492.9	495.3	501.4	518.8	524.0
Waste collection, treatment and disposal services; materials recovery services	340.2	385.1	442.0	413.2	380.7	383.8	580.7	565.0	794.9	931.3	833.7	715.4	732.5	577.9	645.5	632.6	216.7	226.2	229.9	241.0
Remediation services and other waste management services	35.0	49.7	43.0	42.9	41.8	43.0	65.3	82.1	90.2	95.3	97.5	141.5	165.0	209.3	208.2	202.9	244.8	243.3	259.9	293.7
Construction	379.7	429.8	376.1	418.4	439.4	506.9	487.4	554.2	480.1	517.5	562.0	665.8	754.7	780.4	787.3	783.5	852.6	898.1	980.5	1,019.0
Wholesale and retail trade and repair services of motor vehicles and motorcycles	160.8	169.8	175.2	180.6	172.1	178.9	190.0	174.3	154.3	126.7	134.1	157.6	180.1	176.8	195.4	184.8	253.9	287.1	319.9	368.0
Wholesale trade services, except of motor vehicles and motorcycles	-	-	-	-	-	-	-	-	-	-	-	-	-	-	-	-	-	-	-	-
Retail trade services, except of motor vehicles and motorcycles	-	-	-	-	-	-	-	-	-	-	-	-	-	-	-	-	-	-	-	-
Rail transport services	99.5	95.2	120.7	124.5	126.9	127.6	109.4	207.7	177.0	195.8	203.2	243.3	273.2	267.3	222.6	211.6	201.4	193.6	197.6	198.5
Land transport services and transport services via pipelines, excluding rail transport	499.0	531.2	615.9	651.7	664.7	689.8	644.9	736.8	628.3	627.9	709.6	913.3	1,031.8	1,040.6	1,041.9	1,062.8	1,349.6	1,569.8	1,487.1	1,562.5
Water transport services	3.9	3.3	3.5	4.4	4.8	5.6	4.3	4.9	4.9	5.1	4.7	6.2	5.5	4.8	5.6	6.4	6.6	8.4	8.6	8.9
Air transport services	19.5	21.0	22.8	23.9	24.3	19.8	19.6	25.6	17.6	11.3	9.5	8.3	4.9	0.7	0.4	0.2	-	-	-	-
Warehousing and support services for transportation	41.8	42.7	33.7	40.8	36.5	36.4	40.2	41.8	42.3	43.4	45.2	44.1	45.4	47.5	59.3	45.9	46.0	38.0	42.3	62.2
Postal and courier services	120.0	125.5	139.9	145.8	156.0	163.6	164.3	243.6	197.9	163.9	191.3	231.4	293.3	300.8	284.3	276.3	311.2	294.5	303.6	314.7
Accommodation services	26.5	33.4	41.1	44.0	45.6	45.5	48.7	62.4	45.4	33.3	28.3	22.7	15.0	0.9	3.7	6.4	17.9	13.2	14.0	14.0
Food and beverage serving services	71.4	96.9	131.4	150.1	153.6	175.2	185.5	242.0	228.4	255.7	284.7	369.7	373.7	313.2	250.6	231.1	165.1	126.3	151.6	154.2
Publishing services	15.9	14.8	13.3	14.6	10.3	13.9	14.9	21.0	17.0	15.2	5.9	4.6	6.2	6.4	7.5	4.8	3.4	5.9	6.7	8.6
Motion Picture, Video & TV Programme Production, Sound Recording & Music Publishing Activities & Programming And Broadcasting Activities	18.1	20.7	29.7	25.7	26.9	32.0	21.3	45.2	29.0	23.2	27.6	31.5	38.5	41.8	32.3	17.5	6.5	7.6	8.6	8.8
Telecommunications services	339.0	385.0	421.7	444.4	431.0	444.7	435.6	519.5	451.6	457.3	444.6	635.0	691.7	696.9	691.2	739.0	825.2	833.6	866.2	875.3
Computer programming, consultancy and related services	415.4	516.4	576.8	633.8	629.8	715.1	906.6	1,061.5	1,132.4	1,116.4	1,000.9	1,034.4	1,299.2	1,190.1	1,148.8	1,212.9	1,226.1	1,182.9	1,228.0	1,303.6
Information services	44.0	47.5	39.7	47.8	44.1	49.4	51.0	53.4	61.1	63.5	51.0	52.6	70.6	72.2	74.3	78.8	69.2	70.9	70.0	66.8

Sector – UK	1997	1998	1999	2000	2001	2002	2003	2004	2005	2006	2007	2008	2009	2010	2011	2012	2013	2014	2015	2016
Financial services, except insurance and pension funding	332.7	332.6	411.2	402.7	422.2	423.9	660.8	513.7	717.0	911.0	739.7	482.3	707.2	678.2	731.6	798.0	720.0	722.1	720.7	739.6
Insurance and reinsurance, except compulsory social security & Pension funding	274.0	248.6	303.2	306.3	388.7	397.0	445.5	515.6	581.2	595.6	659.8	538.1	474.4	478.7	558.5	625.5	737.3	837.7	858.1	951.2
Services auxiliary to financial services and insurance services	4.4	4.1	4.4	4.4	2.4	2.6	4.8	10.4	8.0	8.4	9.9	7.1	6.7	10.5	16.2	16.3	21.8	26.4	21.9	21.4
Real estate services, excluding on a fee or contract basis and imputed rent	243.8	263.8	310.8	329.7	335.5	368.8	355.0	510.6	534.1	669.0	762.0	952.7	1,046.6	1,008.0	1,018.4	1,059.7	1,191.9	1,233.9	1,285.0	1,315.8
Owner-Occupiers' Housing Services	-	-	-	-	-	-	-	-	-	-	-	-	-	-	-	-	-	-	-	-
Real estate activities on a fee or contract basis	5.8	10.7	9.0	5.3	5.3	5.5	8.5	7.4	6.6	5.2	-	-	-	-	-	-	-	-	-	-
Legal services	515.5	617.3	667.0	664.3	689.8	858.8	846.8	939.8	989.0	991.0	1,110.3	1,466.8	1,699.5	1,663.4	1,714.0	1,559.3	1,695.4	1,961.6	1,729.0	1,800.1
Accounting, bookkeeping and auditing services; tax consulting services	83.8	89.3	100.8	108.2	115.8	118.8	111.7	170.5	145.3	142.5	153.7	190.6	224.6	246.0	245.6	211.1	247.6	288.9	272.3	293.5
Services of head offices; management consulting services	41.8	45.4	43.0	48.0	48.3	60.1	70.0	43.2	42.3	28.7	17.0	15.6	17.5	15.3	23.6	16.9	55.2	70.0	74.9	92.1
Architectural and engineering services; technical testing and analysis services	426.4	507.8	516.6	468.4	445.5	456.9	413.5	426.7	397.6	536.2	719.9	1,036.0	1,329.8	1,522.3	1,530.3	1,588.6	1,701.3	1,874.6	1,776.8	1,856.1
Scientific research and development services	193.9	284.5	300.3	241.3	254.1	245.1	222.1	279.7	537.9	571.9	663.8	856.0	964.6	1,002.0	1,389.2	2,064.3	2,141.0	2,524.8	2,875.1	3,023.4
Advertising and market research services	129.5	147.3	176.6	242.1	256.1	274.9	283.4	309.1	180.4	160.9	104.7	126.3	142.7	149.1	167.8	186.6	215.1	173.5	187.4	203.0
Other professional, scientific and technical services	87.2	100.6	108.9	93.5	99.1	120.3	120.7	119.2	254.7	269.5	291.6	327.0	382.8	379.6	390.7	434.5	627.4	746.7	809.4	867.1
Veterinary services	5.3	7.2	5.9	6.0	4.8	5.0	5.5	10.8	14.5	12.3	7.9	5.7	7.3	5.3	6.1	6.4	6.7	7.0	7.0	6.8
Rental and leasing services	248.8	294.8	309.1	303.2	268.1	282.6	268.4	306.9	307.6	310.1	339.9	421.6	468.4	449.9	496.9	545.2	667.6	682.4	697.7	716.8
Employment services	441.8	520.0	574.7	478.6	508.3	646.7	641.6	681.1	1,397.7	1,524.3	1,614.1	1,936.1	2,071.4	1,929.8	1,962.2	2,070.3	2,234.9	2,441.0	2,404.1	2,394.2
Travel agency, tour operator and other reservation services and related services	37.7	39.9	43.2	44.8	40.5	43.5	40.6	58.0	59.0	50.4	68.3	67.6	74.2	68.3	71.2	55.1	54.3	59.1	58.1	64.1
Security and investigation services	33.6	44.3	49.6	38.3	43.3	57.8	55.2	61.0	143.6	157.6	174.5	215.6	237.3	238.4	237.5	240.8	343.6	395.9	427.4	454.0
Services to buildings and landscape	267.8	314.6	349.6	282.7	308.7	333.4	318.1	389.3	417.1	495.2	537.9	657.7	717.7	715.8	698.1	697.4	813.4	884.6	935.5	976.6
Office administrative, office support and other business support services	161.1	172.8	167.4	144.2	148.4	174.4	191.6	180.4	254.5	261.1	240.3	216.3	243.0	212.5	212.6	233.1	404.7	504.8	549.7	591.3
Public administration and defence services; compulsory social security services	16.3	15.6	16.6	15.9	14.9	21.7	18.8	47.3	59.8	59.9	53.8	63.2	123.2	93.4	115.0	88.6	113.1	129.7	137.6	148.6
Education services	292.1	328.3	266.3	268.8	306.5	328.0	414.4	210.4	196.8	367.9	207.4	194.8	260.0	238.2	402.3	423.1	355.7	368.8	406.5	540.7
Human health services	386.5	390.4	202.9	173.0	105.6	53.1	54.3	60.2	225.1	655.4	540.8	630.8	1,047.2	828.9	730.6	760.8	598.1	700.8	667.8	794.9
Residential Care & Social Work Activities	444.4	458.8	445.5	482.9	385.7	385.2	474.1	617.0	728.1	869.0	949.6	1,013.2	1,340.7	1,513.8	1,373.2	1,409.5	1,337.6	1,409.8	1,430.2	1,454.0
Creative, arts and entertainment services	-	-	6.2	5.5	4.2	4.5	-	-	-	-	7.1	6.6	6.4	2.6	9.7	8.6	3.2	-	-	-
Libraries, archives, museums and other cultural services	2.9	2.9	3.2	3.1	12.1	12.9	14.6	11.4	21.7	13.8	18.6	19.5	17.2	17.7	22.3	25.3	53.5	66.9	72.4	71.0
Gambling and betting services	-	-	-	-	-	-	-	-	-	-	-	-	-	-	-	-	-	-	-	-
Sports services and amusement and recreation services	-	-	-	-	0.9	1.0	1.8	1.8	3.7	-	-	-	-	-	3.2	1.7	-	5.6	5.4	5.3
Services furnished by membership organisations	12.5	12.6	11.2	13.9	11.2	11.6	12.8	10.4	10.3	7.7	7.9	6.0	7.0	8.7	17.5	16.4	17.1	17.5	18.2	23.1
Repair services of computers and personal and household goods	88.8	87.8	118.6	126.7	127.3	134.5	124.6	166.3	216.7	217.4	243.6	319.6	417.7	380.7	321.3	348.7	410.3	400.1	432.4	429.1
Other personal services	67.7	76.5	95.3	95.2	111.9	135.5	148.1	177.9	176.7	189.8	209.0	248.6	287.4	288.0	294.7	299.6	313.1	319.6	330.6	346.7
Services of households as employers of domestic personnel	-	-	-	-	-	-	-	-	-	-	-	-	-	-	-	-	-	-	-	-

Sector – EU	1997	1998	1999	2000	2001	2002	2003	2004	2005	2006	2007	2008	2009	2010	2011	2012	2013	2014	2015	2016
Products of agriculture, hunting and related services	1.2	2.5	2.3	2.0	1.8	2.2	2.0	1.5	2.1	2.4	4.4	6.7	7.2	7.7	6.0	7.0	14.9	10.8	10.6	10.6
Products of forestry, logging and related services	-	-	-	-	-	-	-	-	-	-	-	-	-	-	-	-	-	-	-	-
Fish and other fishing products; aquaculture products; support services to fishing	-	-	-	-	-	-	-	-	-	-	-	-	-	-	-	-	-	-	-	-
Coal and lignite	0.1	0.2	0.2	0.3	0.2	0.3	0.4	0.1	0.0	0.1	0.1	0.5	0.5	0.4	0.4	0.2	0.3	0.4	0.3	0.3
Crude Petroleum And Natural Gas & Metal Ores	-	-	-	0.3	0.9	-	-	-	0.0	-	-	-	-	-	-	-	-	-	-	-
Other mining and quarrying products	0.0	0.8	0.7	0.9	0.6	0.9	1.0	0.6	0.5	0.5	0.9	1.0	-	-	-	-	-	-	-	-
Mining support services	-	-	-	0.0	0.0	-	-	-	-	-	-	-	-	-	-	-	-	-	-	-
Preserved meat and meat products	0.0	-	-	-	0.2	1.8	4.2	3.8	4.7	8.0	9.8	20.0	17.7	23.5	19.6	17.9	30.2	33.6	35.0	33.2
Processed and preserved fish, crustaceans, molluscs, fruit and vegetables	3.4	4.0	4.9	5.1	4.1	5.6	6.2	4.6	5.0	8.2	15.2	18.4	-	-	-	-	-	-	-	-
Vegetable and animal oils and fats	1.4	2.1	0.1	1.8	1.5	1.4	2.5	1.5	1.3	1.7	4.1	8.0	7.5	6.9	6.5	5.8	8.0	8.9	8.1	7.6
Dairy products	5.1	10.7	15.6	15.8	11.1	14.2	19.1	16.0	16.1	23.7	43.3	67.7	50.6	57.8	46.1	38.8	63.8	68.0	63.9	59.8
Grain mill products, starches and starch products	-	-	-	-	-	-	-	-	-	-	-	-	-	-	-	-	-	-	-	-
Bakery and farinaceous products	4.1	5.7	7.9	8.0	6.9	10.4	12.7	10.9	11.7	17.4	29.4	48.1	38.3	39.5	34.4	29.6	42.4	45.3	42.9	41.5
Other food products	9.1	11.7	15.4	15.7	13.8	21.5	25.2	22.5	23.1	32.6	53.2	87.3	71.8	69.4	60.0	51.3	75.7	84.9	79.4	76.4
Prepared animal feeds	2.8	3.2	4.0	4.0	3.5	5.5	5.8	4.6	5.2	7.8	13.2	21.4	17.8	20.9	17.9	16.4	23.9	26.9	25.1	24.1
Alcoholic beverages	33.8	39.6	43.5	34.9	27.2	36.9	48.1	35.9	35.2	46.6	89.7	107.9	79.5	78.9	67.7	61.4	87.6	90.6	82.2	77.0
Soft drinks	13.5	17.9	19.5	16.2	13.7	18.5	21.4	17.5	17.7	23.5	44.9	52.9	33.5	37.2	32.6	29.0	41.3	44.1	40.3	37.3
Tobacco Products	0.0	0.4	0.4	0.4	0.3	0.5	0.5	0.3	0.3	0.4	0.8	0.9	0.9	1.0	0.8	0.6	0.7	0.8	0.7	0.7
Textiles	0.0	-	-	-	-	-	-	-	-	-	-	-	-	-	-	-	-	-	-	0.9
Wearing apparel	-	-	-	-	-	-	-	-	-	-	-	-	-	-	-	-	-	-	-	-
Leather and related products	-	-	-	-	-	-	-	-	-	-	-	-	-	-	-	-	-	-	-	-
Wood and of products of wood and cork, except furniture; articles of straw and plaiting materials	1.8	2.3	2.9	2.5	1.9	2.7	3.3	1.9	2.0	2.5	4.8	5.6	7.5	7.9	6.6	5.5	8.7	10.8	10.5	10.5
Paper and paper products	49.5	79.3	82.8	74.1	71.0	105.9	132.0	74.3	66.9	91.8	171.3	204.6	198.8	208.7	180.6	149.0	217.0	237.0	232.0	229.8
Printing and recording services	18.7	26.1	30.1	32.4	26.6	42.6	45.5	28.6	29.1	39.0	79.1	91.3	65.4	64.1	57.9	44.9	66.1	67.8	70.8	73.7
Coke and refined petroleum products	1.3	1.6	3.5	6.2	5.7	5.8	6.9	5.6	7.5	13.4	32.8	35.7	37.1	47.4	51.1	54.0	65.8	63.4	62.1	62.0
Paints, varnishes and similar coatings, printing ink and mastics	4.7	4.0	11.0	19.5	28.9	34.3	30.4	50.0	42.8	61.6	49.8	90.5	137.7	129.6	104.3	89.1	89.9	95.7	111.9	120.5
Soap and detergents, cleaning and polishing preparations, perfumes and toilet preparations	9.0	8.6	21.8	34.5	52.0	60.8	54.7	89.0	80.4	112.0	94.1	151.8	249.7	229.8	191.4	176.6	180.3	193.2	225.0	249.2
Other chemical products	7.3	7.6	18.7	29.9	43.7	50.0	48.3	67.7	61.6	84.5	71.8	129.0	221.3	213.7	167.6	149.0	155.4	154.5	176.9	201.5
Industrial gases, inorganics and fertilisers (all inorganic chemicals) - 20.11/13/15	4.8	4.1	12.4	20.5	32.7	39.3	31.2	59.1	58.1	87.1	69.7	122.8	140.2	142.7	104.5	93.5	99.3	109.9	132.9	155.6
Petrochemicals - 20.14/16/17/60	18.7	17.8	49.3	80.4	122.7	147.8	135.9	214.6	241.5	332.0	273.0	493.3	765.2	718.4	544.9	359.6	362.5	377.0	487.3	519.9
Dyestuffs, agro-chemicals - 20.12/20	3.4	3.4	8.8	14.0	20.8	25.4	24.4	38.3	33.6	45.9	38.0	66.3	88.4	88.3	74.8	68.1	71.9	72.5	86.9	103.6
Basic pharmaceutical products and pharmaceutical preparations	18.6	18.6	49.9	84.2	138.8	178.0	170.5	236.7	211.4	309.5	252.5	535.3	569.4	626.2	683.3	743.3	727.8	763.6	808.8	829.5
Rubber and plastic products	10.4	18.0	18.5	16.4	13.8	20.9	29.7	24.7	25.2	37.6	74.7	88.4	107.3	108.9	97.7	85.8	137.6	162.8	166.4	172.5
Manufacture of cement, lime, plaster and articles of concrete, cement and plaster	0.0	0.3	0.4	0.4	0.3	0.4	0.5	0.4	0.4	0.5	0.9	1.1	1.0	1.1	0.9	0.7	1.2	1.6	1.7	1.7
Glass, refractory, clay, other porcelain and ceramic, stone and abrasive products - 23.1-4/7-9	6.0	8.2	9.6	9.2	7.1	10.6	13.1	6.5	5.6	9.5	17.1	23.3	18.3	17.2	14.7	10.8	14.7	14.7	11.9	9.2

Sector – EU	1997	1998	1999	2000	2001	2002	2003	2004	2005	2006	2007	2008	2009	2010	2011	2012	2013	2014	2015	2016
**Basic iron and steel**	0.1	0.0	0.1	0.1	0.1	0.1	0.1	0.1	0.1	0.1	0.2	0.3	-	-	-	-	-	-	-	-
**Other basic metals and casting**	0.0	0.9	1.1	1.3	1.1	1.4	1.7	1.0	1.0	1.5	2.6	4.0	-	-	-	-	-	-	-	-
**Weapons and ammunition**	0.3	0.4	0.3	0.4	0.3	0.4	0.4	0.4	0.3	0.4	0.9	1.2	2.7	2.9	2.6	2.4	3.9	4.1	4.1	4.3
**Fabricated metal products, excl. machinery and equipment and weapons & ammunition - 25.1-3/25.5-9**	3.2	4.9	5.8	5.9	4.9	7.3	9.3	4.4	4.1	5.1	9.5	11.5	21.7	23.2	20.0	16.9	27.4	31.1	31.3	31.7
**Computer, electronic and optical products**	143.9	216.7	268.4	293.5	298.4	408.2	478.7	349.2	356.1	564.6	1,062.0	1,332.6	1,406.8	1,536.9	1,667.7	1,805.0	1,759.7	1,839.0	1,874.0	1,915.4
**Electrical equipment**	2.6	3.0	3.1	2.7	2.2	3.8	4.5	4.0	3.5	5.2	10.7	14.7	10.9	12.0	11.1	9.6	14.7	16.2	15.8	15.7
**Machinery and equipment n.e.c.**	0.4	0.6	1.0	1.0	0.9	1.2	1.4	1.1	1.2	1.8	3.9	4.9	13.0	12.3	11.4	10.5	17.2	21.4	22.5	23.8
**Motor vehicles, trailers and semi-trailers**	24.6	22.4	37.3	37.4	36.2	67.9	75.0	58.2	52.4	83.9	145.5	182.3	208.8	244.8	280.9	318.4	325.0	352.1	350.4	352.2
**Ships and boats**	0.4	0.6	0.7	0.6	0.5	0.7	0.9	0.8	0.7	1.0	1.6	2.1	3.0	2.2	2.9	2.2	4.6	5.5	5.5	5.0
**Air and spacecraft and related machinery**	2.2	3.1	4.2	4.3	3.8	4.8	6.7	6.1	5.4	7.1	10.2	12.2	18.6	14.1	18.8	13.5	26.7	31.1	32.0	34.8
**Other transport equipment - 30.2/4/9**	0.2	0.4	0.6	0.6	0.6	0.8	1.2	0.9	0.8	1.1	1.6	1.8	2.8	2.1	2.8	1.9	4.0	4.7	4.8	4.5
**Furniture**	-	-	-	-	-	-	-	-	-	-	-	3.6	-	-	0.3	0.6	-	-	2.7	8.6
**Other manufactured goods**	-	-	-	-	-	-	-	-	-	-	-	3.6	-	-	0.3	0.7	-	-	2.8	9.6
**Repair and maintenance of ships and boats**	0.1	0.2	0.2	0.2	0.2	0.2	0.2	0.2	0.3	0.3	0.4	0.6	0.8	0.6	0.8	0.7	1.4	1.8	2.3	1.8
**Repair and maintenance of aircraft and spacecraft**	0.4	0.6	0.9	1.0	0.9	1.0	1.5	1.2	1.2	1.5	2.2	2.9	3.7	2.9	3.1	2.4	5.3	6.5	6.7	7.5
**Rest of repair; Installation - 33.11-14/17/19/20**	0.3	0.2	0.2	0.3	0.2	0.1	0.2	0.1	0.1	0.2	0.4	0.4	0.3	0.3	0.3	0.3	0.4	0.5	0.4	0.5
**Electricity, transmission and distribution**	-	-	-	6.6	2.4	4.3	4.3	-	-	-	-	-	-	-	-	-	-	-	-	-
**Gas; distribution of gaseous fuels through mains; steam and air conditioning supply**	-	-	-	-	-	-	-	-	-	-	-	-	-	-	-	-	-	-	-	-
**Natural water; water treatment and supply services**	-	-	-	-	-	-	-	-	-	-	-	-	-	-	-	-	-	-	-	-
**Sewerage services; sewage sludge**	1.0	0.9	0.9	0.8	1.0	1.8	0.9	1.1	1.2	1.6	4.5	4.3	5.7	4.9	4.0	3.9	5.8	6.5	6.5	6.7
**Waste collection, treatment and disposal services; materials recovery services**	2.0	1.9	2.0	2.0	2.5	4.8	2.4	3.3	3.8	5.3	14.2	12.9	16.5	14.9	12.5	11.2	15.9	17.6	17.5	17.5
**Remediation services and other waste management services**	0.0	0.0	0.0	0.0	0.0	0.0	0.0	0.0	0.0	0.0	0.1	0.1	0.2	0.2	0.1	0.1	0.2	0.3	0.3	0.4
**Construction**	1.8	2.3	2.0	2.2	2.6	2.3	4.6	1.9	1.8	2.7	4.4	4.8	5.3	5.0	4.2	3.9	5.2	5.4	5.3	5.2
**Wholesale and retail trade and repair services of motor vehicles and motorcycles**	0.9	0.7	0.8	0.8	0.8	0.8	0.9	0.8	0.8	1.2	2.6	2.8	2.7	2.6	2.5	2.3	3.2	3.3	3.3	3.2
**Wholesale trade services, except of motor vehicles and motorcycles**	0.1	0.2	0.2	0.1	0.1	0.2	0.2	0.2	0.2	0.3	0.6	0.6	0.8	0.8	0.7	0.6	0.9	1.0	1.0	1.0
**Retail trade services, except of motor vehicles and motorcycles**	1.7	1.6	1.7	1.7	1.7	1.6	1.8	1.6	1.8	2.6	5.6	5.3	4.9	4.9	4.8	4.6	6.4	6.8	7.0	7.0
**Rail transport services**	0.9	1.0	1.1	1.1	1.1	2.5	2.3	2.1	2.0	3.4	5.9	6.5	6.0	6.3	4.7	4.5	6.4	7.1	7.1	7.3
**Land transport services and transport services via pipelines, excluding rail transport**	13.1	17.2	19.1	18.3	24.4	47.4	46.6	43.2	37.8	68.8	124.2	141.9	167.3	175.3	129.7	123.6	178.4	199.3	200.4	204.9
**Water transport services**	1.8	2.3	2.4	2.3	1.8	2.9	3.5	2.7	2.5	3.2	4.9	5.9	11.1	8.8	7.4	7.1	9.9	11.0	11.0	11.2
**Air transport services**	3.5	4.6	5.6	5.1	3.7	5.1	7.5	5.6	5.5	8.1	11.5	13.4	12.7	13.2	12.7	11.9	16.5	16.9	17.3	17.8
**Warehousing and support services for transportation**	1.2	1.6	1.9	1.8	2.1	5.3	5.9	2.3	2.4	4.0	8.2	9.0	6.7	6.8	5.6	5.2	7.0	7.7	7.5	7.4
**Postal and courier services**	2.5	3.4	3.7	3.4	4.4	7.0	9.1	6.9	7.2	7.8	12.0	14.5	15.2	16.7	13.7	12.4	16.4	17.0	15.7	14.9
**Accommodation services**	4.6	7.1	8.5	8.7	7.1	11.5	19.9	12.9	11.7	19.2	37.3	43.8	147.6	136.3	127.2	128.7	187.5	209.7	219.4	236.2
**Food and beverage serving services**	12.9	20.0	23.8	24.4	20.3	32.9	57.5	36.7	33.1	52.8	103.6	122.0	398.5	375.6	361.6	357.1	525.8	571.1	602.9	642.5
**Publishing services**	5.2	7.3	7.7	6.7	6.0	9.0	15.6	8.2	8.2	9.4	19.4	25.7	31.6	28.5	24.7	22.4	30.0	32.8	31.3	31.8
**Motion Picture, Video & TV Programme Production, Sound Recording & Music Publishing Activities & Programming And Broadcasting Activities**	2.2	2.6	3.3	3.1	3.2	5.5	8.7	5.1	5.9	8.4	17.1	20.3	15.1	14.1	12.0	10.6	13.8	14.1	13.6	13.1
**Telecommunications services**	6.2	9.0	10.5	10.3	12.8	19.6	25.9	20.3	21.7	22.7	35.3	42.0	39.5	42.2	33.3	29.7	39.6	42.6	40.2	38.0
**Computer programming, consultancy and related services**	2.9	4.4	6.4	6.4	6.5	11.1	18.7	11.1	12.5	19.5	39.6	46.0	32.0	28.3	26.4	22.5	30.1	32.5	30.4	29.0

Sector – EU	1997	1998	1999	2000	2001	2002	2003	2004	2005	2006	2007	2008	2009	2010	2011	2012	2013	2014	2015	2016
Information services	0.5	0.6	0.8	0.8	0.8	1.5	2.4	1.4	1.6	2.5	5.2	6.3	4.3	4.1	3.5	3.1	4.2	4.4	4.1	3.8
Financial services, except insurance and pension funding	1.5	2.5	2.0	1.7	1.5	2.8	3.6	3.7	4.4	6.7	13.4	17.8	18.9	18.5	17.0	16.0	23.8	29.6	30.0	30.3
Insurance and reinsurance, except compulsory social security & Pension funding	2.6	3.4	3.7	4.1	3.9	5.7	5.9	8.1	6.7	8.9	16.8	18.2	16.3	19.1	14.0	12.4	16.1	16.6	14.6	12.7
Services auxiliary to financial services and insurance services	0.0	0.1	0.1	0.1	0.1	0.2	0.2	0.1	0.1	0.2	0.4	0.6	0.3	0.3	0.3	0.3	0.4	0.4	0.5	0.5
Real estate services, excluding on a fee or contract basis and imputed rent	0.5	0.5	0.5	0.5	1.0	1.6	1.8	1.0	1.4	3.4	5.2	4.7	5.9	5.6	4.7	4.8	6.9	7.4	7.5	7.6
Owner-Occupiers' Housing Services	1.7	1.5	1.7	1.7	3.3	5.2	5.7	3.3	4.5	10.3	14.9	13.0	15.2	13.5	10.6	10.1	14.0	15.0	15.1	15.2
Real estate activities on a fee or contract basis	0.0	0.0	0.1	0.1	0.1	0.2	0.2	0.1	0.2	0.5	0.7	0.6	0.7	0.7	0.5	0.5	0.6	0.7	0.7	0.8
Legal services	3.2	4.7	5.1	4.6	4.7	7.8	14.8	8.1	8.4	10.5	23.2	30.9	40.1	35.6	32.6	31.6	43.6	48.3	47.6	47.1
Accounting, bookkeeping and auditing services; tax consulting services	2.4	3.7	3.8	3.6	3.6	5.6	9.9	5.4	5.6	7.1	15.2	20.6	27.4	25.1	22.9	21.7	30.2	33.6	33.1	34.4
Services of head offices; management consulting services	2.7	4.2	4.7	4.3	4.5	7.2	13.7	7.5	8.3	11.0	24.9	36.5	46.6	42.3	38.2	35.1	50.1	56.4	57.9	58.9
Architectural and engineering services; technical testing and analysis services	5.6	8.1	8.3	7.2	6.9	11.4	21.1	11.7	13.0	16.2	35.6	48.8	62.0	55.7	49.8	48.4	66.9	73.1	71.4	74.4
Scientific research and development services	1.5	3.7	3.2	6.0	8.3	39.6	96.0	62.4	92.3	109.8	216.6	296.5	311.9	266.7	220.1	213.2	303.8	338.0	341.6	351.3
Advertising and market research services	1.7	2.6	2.9	2.7	2.9	4.8	8.4	4.4	4.5	5.4	12.1	17.0	21.8	20.7	18.8	17.6	24.7	29.0	29.1	28.9
Other professional, scientific and technical services	1.6	2.5	2.8	2.7	2.5	4.0	7.4	4.0	4.2	5.3	11.5	15.4	20.8	19.2	17.1	16.4	23.3	27.1	27.1	26.7
Veterinary services	0.4	0.6	0.7	0.6	0.6	0.9	1.7	0.9	1.0	1.1	2.6	3.4	4.7	4.3	3.9	3.9	5.6	6.7	6.7	7.3
Rental and leasing services	2.2	3.5	3.8	3.6	4.6	4.8	6.0	7.7	7.3	13.0	22.6	25.2	25.6	23.0	18.7	17.5	24.8	27.8	28.0	28.0
Employment services	3.4	5.5	6.1	5.7	5.4	8.5	15.8	8.5	8.7	10.8	23.5	34.3	37.3	34.0	30.0	28.1	41.1	49.3	52.3	46.8
Travel agency, tour operator and other reservation services and related services	2.6	3.8	4.0	3.5	3.3	5.3	9.8	5.3	5.6	6.7	14.4	19.8	23.3	19.5	18.0	17.6	24.3	26.8	25.6	28.6
Security and investigation services	0.6	0.9	1.1	1.0	1.0	1.5	2.7	1.5	1.5	1.9	4.0	5.5	7.3	6.6	5.9	5.5	7.3	8.2	8.1	8.1
Services to buildings and landscape	2.0	2.9	3.0	2.6	2.5	4.0	7.3	4.0	4.0	5.1	11.3	16.0	22.4	21.4	19.7	19.0	26.2	28.6	28.6	28.6
Office administrative, office support and other business support services	3.4	5.3	6.0	5.6	5.2	8.1	14.8	8.0	8.4	10.4	22.7	32.6	41.7	39.8	37.7	36.0	50.1	55.3	53.5	53.7
Public administration and defence services; compulsory social security services	0.2	1.1	1.2	1.2	1.2	1.5	1.9	0.7	0.6	0.8	1.7	2.1	1.4	1.8	1.4	1.3	1.7	1.8	1.7	1.7
Education services	2.3	2.6	3.0	3.2	7.0	4.0	7.1	1.6	2.8	5.3	9.4	12.5	7.1	7.8	3.4	3.1	4.2	4.3	4.1	3.9
Human health services	32.5	44.4	54.1	58.8	79.8	45.7	69.5	9.6	16.3	50.4	62.4	102.2	118.1	119.5	72.6	70.4	100.6	106.9	105.6	108.5
Residential Care & Social Work Activities	14.9	20.4	25.8	27.8	37.2	21.8	33.7	4.5	7.6	22.9	28.5	43.3	50.5	47.9	29.1	29.8	42.1	44.5	44.4	43.8
Creative, arts and entertainment services	0.2	0.3	0.3	0.3	0.3	0.4	0.4	0.4	0.3	0.3	0.6	0.9	0.3	0.3	-	-	-	-	-	-
Libraries, archives, museums and other cultural services	0.1	0.2	0.2	0.2	0.2	0.3	0.3	0.2	0.2	0.2	0.4	0.6	0.2	0.2	-	-	-	-	-	-
Gambling and betting services	0.3	0.5	0.6	0.6	0.6	0.8	0.8	0.7	0.5	0.7	1.3	1.7	0.5	0.5	-	-	-	-	-	-
Sports services and amusement and recreation services	0.3	0.4	0.5	0.6	0.6	0.6	0.7	0.6	0.5	0.5	1.0	1.3	0.4	0.4	-	-	-	-	-	-
Services furnished by membership organisations	-	-	-	-	-	-	-	-	-	-	1.9	2.5	-	-	-	-	-	-	-	-
Repair services of computers and personal and household goods	0.1	0.1	0.1	0.1	0.4	0.4	0.6	0.1	0.1	0.1	0.3	0.3	0.3	0.2	0.2	0.2	0.3	0.3	0.4	0.4
Other personal services	0.4	0.4	0.5	0.5	2.4	2.4	3.9	0.6	0.7	0.8	1.8	2.1	1.6	1.7	1.3	1.2	1.7	1.9	1.9	1.9
Services of households as employers of domestic personnel	-	-	-	-	-	-	-	-	-	-	-	-	-	-	-	-	-	-	-	-

**Sector – China**	**1997**	**1998**	**1999**	**2000**	**2001**	**2002**	**2003**	**2004**	**2005**	**2006**	**2007**	**2008**	**2009**	**2010**	**2011**	**2012**	**2013**	**2014**	**2015**	**2016**
Products of agriculture, hunting and related services	0.0	0.0	0.0	0.0	0.0	0.0	0.0	0.0	0.0	0.0	0.1	0.2	0.2	0.2	0.2	0.1	0.2	0.4	0.1	0.1
Products of forestry, logging and related services	-	-	-	-	-	-	-	-	-	-	-	-	-	-	-	-	-	-	-	-
Fish and other fishing products; aquaculture products; support services to fishing	-	-	-	-	-	-	-	-	-	-	-	-	-	-	-	-	-	-	-	-
Coal and lignite	-	-	-	-	-	-	-	-	-	-	-	-	-	-	-	-	-	-	-	-
Crude Petroleum And Natural Gas & Metal Ores	-	-	-	-	-	-	-	-	0.0	-	-	-	-	-	-	-	-	-	-	-
Other mining and quarrying products	0.0	0.0	0.0	0.0	0.0	0.0	0.1	0.1	0.0	0.1	0.2	0.2	-	-	-	-	-	-	-	-
Mining support services	-	-	-	-	-	-	-	-	-	-	-	-	-	-	-	-	-	-	-	-
Preserved meat and meat products	-	-	-	-	-	-	-	-	-	-	-	0.0	-	-	-	-	-	-	-	-
Processed and preserved fish, crustaceans, molluscs, fruit and vegetables	0.0	0.1	0.1	0.2	0.3	0.2	0.6	0.5	0.6	1.3	2.8	3.7	-	-	-	-	-	-	-	-
Vegetable and animal oils and fats	-	0.0	0.0	0.0	0.0	0.0	0.0	0.0	0.0	0.0	0.0	0.2	0.1	0.1	0.1	0.0	0.1	0.1	0.1	0.1
Dairy products	-	-	-	0.0	-	-	-	-	-	-	0.0	-	-	-	0.0	0.0	-	0.0	0.0	0.0
Grain mill products, starches and starch products	-	-	-	-	-	-	-	-	-	-	-	-	-	-	-	-	-	-	-	-
Bakery and farinaceous products	0.0	0.0	0.1	0.1	0.1	0.1	0.1	0.1	0.1	0.3	0.5	0.8	0.6	0.9	0.7	0.6	0.9	0.9	0.8	0.7
Other food products	0.1	0.1	0.1	0.1	0.1	0.2	0.2	0.2	0.3	0.5	0.9	1.4	1.1	1.5	1.3	1.1	1.7	1.7	1.4	1.3
Prepared animal feeds	0.0	0.0	0.0	0.0	0.0	0.1	0.1	0.1	0.1	0.1	0.2	0.4	0.3	0.5	0.4	0.4	0.5	0.5	0.5	0.4
Alcoholic beverages	0.0	0.0	0.0	0.0	0.0	0.1	0.0	0.0	0.0	0.1	0.1	0.1	0.1	0.3	0.2	0.2	0.2	0.2	0.1	0.1
Soft drinks	0.0	0.0	0.0	0.0	0.0	0.1	0.0	0.0	0.0	0.0	0.1	0.1	0.1	0.2	0.1	0.1	0.1	0.1	0.1	0.1
Tobacco products	0.0	0.0	0.0	0.0	0.0	0.0	0.0	0.0	0.0	0.0	0.0	0.0	0.0	0.0	0.0	0.0	0.0	0.0	0.0	0.0
Textiles	0.0	-	-	-	-	-	-	-	-	-	-	-	-	-	-	-	-	-	-	0.5
Wearing apparel	-	-	-	-	-	-	-	-	-	-	-	-	-	-	-	-	-	-	-	-
Leather and related products	-	-	-	-	-	-	-	-	-	-	-	-	-	-	-	-	-	-	-	-
Wood and of products of wood and cork, except furniture; articles of straw and plaiting materials	0.0	0.0	0.0	0.1	0.1	0.1	0.1	0.1	0.1	0.2	0.4	0.7	1.2	1.4	1.3	1.2	1.8	2.0	1.8	1.8
Paper and paper products	0.1	0.2	0.2	0.5	0.6	1.1	1.8	1.3	1.5	2.4	6.7	8.0	8.4	9.1	9.2	8.8	14.3	16.6	15.2	14.7
Printing and recording services	0.2	0.3	0.3	1.0	1.1	1.9	2.9	2.1	2.6	4.3	9.4	12.0	10.1	10.5	10.5	9.4	13.7	15.1	14.4	14.4
Coke and refined petroleum products	0.0	0.0	0.0	0.0	0.0	0.0	0.0	0.0	0.0	0.0	0.0	0.1	0.0	0.2	0.3	0.4	0.1	0.1	0.1	0.1
Paints, varnishes and similar coatings, printing ink and mastics	0.1	0.1	0.3	0.9	1.4	1.4	1.6	3.1	3.8	5.7	7.0	11.6	16.2	20.8	21.7	21.9	22.8	20.4	21.3	21.8
Soap and detergents, cleaning and polishing preparations, perfumes and toilet preparations	0.2	0.2	0.5	1.7	2.5	2.5	2.8	5.4	7.1	10.4	13.2	19.5	29.4	37.0	39.8	43.4	45.8	41.3	42.8	45.0
Other chemical products	0.2	0.2	0.5	1.4	2.1	2.1	2.5	4.1	5.4	7.9	10.1	16.6	26.1	34.4	34.9	36.6	39.5	33.0	33.7	36.4
Industrial gases, inorganics and fertilisers (all inorganic chemicals) - 20.11/13/15	0.1	0.1	0.3	1.0	1.5	1.6	1.6	3.6	5.1	8.1	9.8	15.8	16.5	22.9	21.7	23.0	25.2	23.5	25.3	28.1
Petrochemicals - 20.14/16/17/60	0.4	0.4	1.2	3.9	5.8	6.2	7.0	13.1	21.3	30.9	38.4	63.5	90.2	115.6	113.4	88.3	92.0	80.5	92.7	94.0
Dyestuffs, agro-chemicals - 20.12/20	0.1	0.1	0.2	0.7	1.0	1.1	1.2	2.3	3.0	4.3	5.3	8.5	10.4	14.2	15.6	16.7	18.3	15.5	16.5	18.7
Basic pharmaceutical products and pharmaceutical preparations	0.4	0.4	1.2	4.1	6.5	7.4	8.7	14.5	18.6	28.8	35.5	68.9	112.4	147.8	148.7	144.8	146.1	137.4	130.5	149.9
Rubber and plastic products	0.2	0.5	0.5	1.3	1.2	1.7	3.0	2.4	2.8	4.6	10.8	13.7	18.5	21.6	20.9	20.5	33.9	38.9	37.4	38.0
Manufacture of cement, lime, plaster and articles of concrete, cement and plaster	0.0	0.0	0.0	0.0	0.0	0.0	0.0	0.0	0.0	0.0	0.1	0.0	0.0	0.1	0.1	0.0	0.1	0.1	0.1	0.1
Glass, refractory, clay, other porcelain and ceramic, stone and abrasive products - 23.1-4/7-9	0.1	0.1	0.2	0.3	0.4	0.7	1.1	0.7	0.9	2.0	3.9	6.2	5.1	6.0	4.9	4.0	5.6	4.7	3.5	2.6
Basic iron and steel	0.0	0.0	0.0	0.0	0.0	0.0	0.0	0.0	0.0	0.0	0.0	0.0	-	-	-	-	-	-	-	-
Other basic metals and casting	0.0	0.0	0.0	0.1	0.0	0.0	0.0	0.0	0.0	0.1	0.2	0.3	-	-	-	-	-	-	-	-
Weapons and ammunition	0.0	0.0	0.0	0.0	0.0	0.0	0.0	0.1	0.1	0.1	0.2	0.3	0.6	0.7	0.7	0.7	1.0	1.1	1.1	1.1
Fabricated metal products, excl. machinery and equipment and weapons & ammunition - 25.1-3/25.5-9	0.1	0.2	0.2	0.4	0.4	0.7	1.1	0.6	0.6	1.0	2.2	2.9	4.9	5.7	5.1	4.5	6.9	8.6	8.1	8.0
Computer, electronic and optical products	1.8	3.3	4.3	19.1	18.5	29.0	40.9	28.9	34.3	53.2	108.7	151.0	136.8	166.2	156.9	162.2	262.6	295.5	291.9	298.8
Electrical equipment	0.1	0.1	0.1	0.3	0.3	0.5	0.7	0.6	0.6	1.0	2.4	3.3	2.5	3.0	3.1	2.7	4.1	4.8	4.4	4.3

Sector – China	1997	1998	1999	2000	2001	2002	2003	2004	2005	2006	2007	2008	2009	2010	2011	2012	2013	2014	2015	2016
Machinery and equipment n.e.c.	0.0	0.0	0.0	0.0	0.0	0.1	0.1	0.1	0.1	0.2	0.5	0.7	2.0	2.4	2.1	2.1	3.4	4.1	4.0	4.2
Motor vehicles, trailers and semi-trailers	0.0	0.0	0.0	0.0	0.1	0.1	0.1	0.1	0.2	0.3	0.8	1.9	1.1	2.0	2.5	2.3	3.8	5.1	4.7	4.7
Ships and boats	0.0	0.0	0.0	0.0	0.0	0.0	0.0	0.0	0.0	0.1	0.1	0.1	0.1	0.1	0.2	0.1	0.2	0.4	0.4	0.3
Air and spacecraft and related machinery	0.0	0.1	0.1	0.1	0.2	0.2	0.3	0.3	0.2	0.5	0.8	0.7	0.8	0.8	1.5	0.8	1.3	2.1	2.1	2.2
Other transport equipment - 30.2/4/9	0.0	0.0	0.0	0.0	0.0	0.0	0.1	0.0	0.0	0.1	0.1	0.1	0.1	0.1	0.2	0.1	0.2	0.3	0.3	0.3
Furniture	-	-	-	-	-	-	-	-	-	-	-	2.6	-	-	0.2	0.4	-	-	1.6	4.7
Other manufactured goods	-	-	-	-	-	-	-	-	-	-	-	2.7	-	-	0.2	0.4	-	-	1.6	5.3
Repair and maintenance of ships and boats	0.0	0.0	0.0	0.0	0.0	0.0	0.0	0.0	0.0	0.0	0.0	0.0	0.0	0.0	0.1	0.0	0.1	0.1	0.1	0.1
Repair and maintenance of aircraft and spacecraft	0.0	0.0	0.0	0.0	0.0	0.1	0.1	0.1	0.1	0.1	0.2	0.2	0.2	0.2	0.3	0.1	0.2	0.5	0.4	0.5
Rest of repair; Installation - 33.11-14/17/19/20	0.1	0.1	0.1	0.1	0.0	0.0	0.0	0.0	0.0	0.1	0.1	0.1	0.1	0.1	0.1	0.1	0.1	0.1	0.1	0.1
Electricity, transmission and distribution	-	-	-	-	-	-	-	-	-	-	-	-	-	-	-	-	-	-	-	-
Gas; distribution of gaseous fuels through mains; steam and air conditioning supply	-	-	-	-	-	-	-	-	-	-	-	-	-	-	-	-	-	-	-	-
Natural water; water treatment and supply services	-	-	-	-	-	-	-	-	-	-	-	-	-	-	-	-	-	-	-	-
Sewerage services; sewage sludge	0.0	0.0	0.0	0.0	0.0	0.0	0.0	0.0	0.0	0.0	0.0	0.0	0.0	0.0	0.0	0.0	0.1	0.1	0.1	0.1
Waste collection, treatment and disposal services; materials recovery services	0.0	0.0	0.0	0.0	0.0	0.0	0.0	0.0	0.0	0.0	0.0	0.0	0.1	0.1	0.1	0.1	0.2	0.2	0.2	0.2
Remediation services and other waste management services	0.0	0.0	0.0	0.0	0.0	0.0	0.0	0.0	0.0	0.0	0.0	0.0	0.0	0.0	0.0	0.0	0.0	0.0	0.0	0.0
Construction	0.0	0.0	0.0	0.0	0.0	0.1	0.1	0.1	0.1	0.1	0.4	0.8	0.8	1.2	1.0	0.9	1.2	1.2	1.1	1.0
Wholesale and retail trade and repair services of motor vehicles and motorcycles	0.2	0.1	0.2	0.2	0.2	0.2	0.2	0.2	0.2	0.3	0.6	0.7	0.6	0.7	0.7	0.6	0.8	0.8	0.7	0.7
Wholesale trade services, except of motor vehicles and motorcycles	0.0	0.0	0.0	0.0	0.0	0.0	0.0	0.0	0.0	0.0	0.0	0.0	0.0	0.0	0.0	0.0	0.0	0.0	0.0	0.0
Retail trade services, except of motor vehicles and motorcycles	0.4	0.5	0.5	0.5	0.4	0.3	0.4	0.5	0.4	0.7	1.7	2.0	1.4	1.5	1.7	1.5	2.0	2.0	1.8	1.7
Rail transport services	0.0	0.0	0.0	0.0	0.0	0.0	0.0	0.0	0.0	0.0	0.1	0.1	0.1	0.1	0.1	0.1	0.1	0.1	0.1	0.1
Land transport services and transport services via pipelines, excluding rail transport	0.2	0.4	0.4	0.5	0.2	0.3	0.4	0.4	0.4	0.4	0.9	1.3	1.7	2.4	2.3	2.0	3.0	3.1	2.9	2.8
Water transport services	0.1	0.2	0.2	0.2	0.2	0.3	0.4	0.4	0.5	0.7	1.4	1.8	2.8	2.3	1.8	1.6	2.3	2.4	2.3	2.2
Air transport services	0.1	0.1	0.1	0.1	0.1	0.1	0.2	0.2	0.3	0.4	0.8	1.1	0.9	1.6	1.4	1.3	1.8	1.7	1.6	1.6
Warehousing and support services for transportation	0.1	0.1	0.1	0.1	0.0	0.0	0.1	0.1	0.1	0.1	0.2	0.3	0.1	0.1	0.1	0.1	0.1	0.1	0.1	0.1
Postal and courier services	0.2	0.3	0.4	0.3	0.1	0.1	0.1	0.0	0.0	0.1	0.2	0.3	0.2	0.2	0.2	0.2	0.3	0.3	0.2	0.2
Accommodation services	0.3	0.6	0.7	0.8	0.7	1.2	1.3	1.2	1.2	2.1	3.7	4.8	19.1	20.4	19.8	18.9	28.2	29.6	29.0	30.4
Food and beverage serving services	0.9	1.7	2.1	2.2	2.1	3.4	3.6	3.5	3.4	5.8	10.4	13.3	51.6	56.3	56.3	52.5	79.1	80.5	79.6	82.6
Publishing services	0.0	0.0	0.0	0.0	0.0	0.1	0.2	0.1	0.2	0.4	1.0	1.6	1.8	2.1	2.2	1.8	2.5	2.6	2.3	2.2
Motion Picture, Video & TV Programme Production, Sound Recording & Music Publishing Activities & Programming And Broadcasting Activities	0.0	0.0	0.0	0.0	0.1	0.1	0.2	0.1	0.1	0.3	0.7	1.1	0.9	1.3	1.4	1.2	1.5	1.4	1.3	1.2
Telecommunications services	0.5	0.9	1.0	1.1	0.2	0.3	0.3	0.1	0.1	0.2	0.5	0.7	0.5	0.5	0.6	0.5	0.7	0.7	0.6	0.5
Computer programming, consultancy and related services	0.0	0.1	0.1	0.1	0.1	0.2	0.5	0.3	0.3	0.6	1.6	2.6	1.9	2.6	3.0	2.5	3.3	3.3	2.9	2.7
Information services	0.0	0.0	0.0	0.0	0.0	0.0	0.1	0.0	0.0	0.1	0.2	0.3	0.3	0.4	0.4	0.3	0.5	0.4	0.4	0.3
Financial services, except insurance and pension funding	0.0	0.0	0.0	0.0	0.0	0.0	0.0	0.0	0.0	0.0	0.0	0.0	0.0	0.2	0.1	0.1	0.1	0.1	0.1	0.1
Insurance and reinsurance, except compulsory social security & Pension funding	0.0	0.0	0.0	0.0	0.1	0.1	0.1	0.1	0.2	0.3	0.6	1.0	0.6	1.0	1.3	1.1	1.4	1.3	1.1	0.9
Services auxiliary to financial services and insurance services	0.0	0.0	0.0	0.0	0.0	0.0	0.0	0.0	0.0	0.0	0.0	0.0	0.0	0.0	0.0	0.0	0.0	0.0	0.0	0.0
Real estate services, excluding on a fee or contract basis and imputed rent	0.0	0.0	0.0	0.0	0.0	0.0	0.0	0.0	0.0	0.0	0.0	0.1	0.1	0.1	0.1	0.1	0.2	0.2	0.2	0.2
Owner-Occupiers' Housing Services	0.0	0.0	0.0	0.0	0.0	0.0	0.0	0.0	0.1	0.1	0.1	0.2	0.2	0.2	0.3	0.3	0.4	0.4	0.3	0.3
Real estate activities on a fee or contract basis	0.0	0.0	0.0	0.0	0.0	0.0	0.0	0.0	0.0	0.0	0.0	0.0	0.0	0.0	0.0	0.0	0.0	0.0	0.0	0.0
Legal services	0.0	0.0	0.0	0.0	0.0	0.1	0.1	0.1	0.2	0.4	1.1	1.9	2.3	2.6	2.9	2.6	3.7	3.8	3.4	3.3

Sector – China	1997	1998	1999	2000	2001	2002	2003	2004	2005	2006	2007	2008	2009	2010	2011	2012	2013	2014	2015	2016
Accounting, bookkeeping and auditing services; tax consulting services	0.0	0.0	0.0	0.0	0.0	0.1	0.1	0.1	0.1	0.3	0.8	1.3	1.6	1.8	2.0	1.8	2.5	2.6	2.4	2.4
Services of head offices; management consulting services	0.0	0.0	0.0	0.0	0.0	0.1	0.1	0.1	0.2	0.4	1.2	2.3	2.6	3.1	3.4	2.9	4.2	4.4	4.2	4.1
Architectural and engineering services; technical testing and analysis services	0.0	0.0	0.0	0.0	0.1	0.1	0.2	0.2	0.3	0.6	1.8	3.1	3.5	4.1	4.4	4.0	5.6	5.7	5.2	5.2
Scientific research and development services	0.0	0.0	0.0	0.1	0.1	0.3	0.5	0.3	0.5	0.8	3.9	6.8	11.0	13.4	13.9	12.6	18.5	19.0	17.9	17.9
Advertising and market research services	0.0	0.0	0.0	0.0	0.0	0.0	0.1	0.1	0.1	0.2	0.6	1.1	1.2	1.5	1.7	1.4	2.1	2.3	2.1	2.0
Other professional, scientific and technical services	0.0	0.0	0.0	0.0	0.0	0.0	0.1	0.1	0.1	0.2	0.6	1.0	1.2	1.4	1.5	1.3	2.0	2.1	2.0	1.9
Veterinary services	0.0	0.0	0.0	0.0	0.0	0.0	0.0	0.0	0.0	0.0	0.1	0.2	0.3	0.3	0.4	0.3	0.5	0.5	0.5	0.5
Rental and leasing services	0.0	0.0	0.0	0.0	0.0	0.1	0.1	0.1	0.1	0.2	0.3	0.4	0.6	0.8	0.5	0.4	0.6	0.7	0.6	0.6
Employment services	0.0	0.0	0.0	0.0	0.0	0.1	0.2	0.1	0.2	0.4	1.2	2.2	2.1	2.5	2.7	2.3	3.5	3.9	3.8	3.3
Travel agency, tour operator and other reservation services and related services	0.0	0.0	0.0	0.0	0.0	0.1	0.1	0.1	0.1	0.3	0.7	1.2	1.3	1.4	1.6	1.4	2.0	2.1	1.8	2.0
Security and investigation services	0.0	0.0	0.0	0.0	0.0	0.0	0.0	0.0	0.0	0.1	0.2	0.3	0.4	0.5	0.5	0.5	0.6	0.6	0.6	0.6
Services to buildings and landscape	0.0	0.0	0.0	0.0	0.0	0.0	0.1	0.1	0.1	0.2	0.6	1.0	1.3	1.6	1.8	1.6	2.2	2.2	2.1	2.0
Office administrative, office support and other business support services	0.0	0.0	0.0	0.0	0.0	0.1	0.2	0.1	0.2	0.4	1.1	2.1	2.4	2.9	3.4	2.9	4.2	4.3	3.9	3.7
Public administration and defence services; compulsory social security services	0.0	0.0	0.0	0.0	0.1	0.0	0.0	0.0	0.0	0.0	0.1	0.1	0.1	0.1	0.1	0.1	0.1	0.1	0.1	0.1
Education services	0.0	0.0	0.0	0.0	0.0	0.0	0.0	0.0	0.0	0.0	0.0	0.0	0.0	0.0	0.1	0.1	0.1	0.1	0.1	0.1
Human health services	0.0	0.0	0.1	0.1	0.1	0.0	0.0	0.0	0.0	0.1	0.1	0.2	0.2	0.2	2.2	2.0	2.8	2.7	2.4	2.4
Residential Care & Social Work Activities	0.0	0.0	0.0	0.0	0.0	0.0	0.0	0.0	0.0	0.0	0.0	0.1	0.1	0.1	0.9	0.8	1.2	1.1	1.0	1.0
Creative, arts and entertainment services	0.0	0.0	0.0	0.0	0.0	0.0	0.0	0.0	0.0	0.0	0.0	0.0	0.0	0.0	-	-	-	-	-	-
Libraries, archives, museums and other cultural services	0.0	0.0	0.0	0.0	0.0	0.0	0.0	0.0	0.0	0.0	0.0	0.0	0.0	0.0	-	-	-	-	-	-
Gambling and betting services	0.0	0.0	0.0	0.0	0.0	0.0	0.0	0.0	0.0	0.0	0.0	0.0	0.0	0.0	-	-	-	-	-	-
Sports services and amusement and recreation services	0.0	0.0	0.0	0.0	0.0	0.0	0.0	0.0	0.0	0.0	0.0	0.0	0.0	0.0	-	-	-	-	-	-
Services furnished by membership organisations	-	-	-	-	-	-	-	-	-	-	0.1	0.1	-	-	-	-	-	-	-	-
Repair services of computers and personal and household goods	0.0	0.0	0.0	0.0	0.0	0.0	0.0	0.0	0.0	0.0	0.0	0.0	0.0	0.0	0.0	0.0	0.0	0.0	0.0	0.0
Other personal services	0.0	0.0	0.0	0.0	0.0	0.0	0.0	0.0	0.0	0.0	0.0	0.0	0.0	0.0	0.1	0.1	0.2	0.2	0.2	0.2
Services of households as employers of domestic personnel	-	-	-	-	-	-	-	-	-	-	-	-	-	-	-	-	-	-	-	-

Sector – Rest-of-World	1997	1998	1999	2000	2001	2002	2003	2004	2005	2006	2007	2008	2009	2010	2011	2012	2013	2014	2015	2016
Products of agriculture, hunting and related services	1.3	1.8	2.1	1.3	1.5	2.3	1.5	1.5	2.3	2.9	4.0	8.2	9.0	8.9	9.5	7.9	10.5	9.0	8.1	8.0
Products of forestry, logging and related services	-	-	-	-	-	-	-	-	-	-	-	-	-	-	-	-	-	-	-	-
Fish and other fishing products; aquaculture products; support services to fishing	-	-	-	-	-	-	-	-	-	-	-	-	-	-	-	-	-	-	-	-
Coal and lignite	0.0	0.0	0.0	0.0	0.0	0.0	0.0	0.0	-	0.0	0.0	0.2	0.0	0.1	0.0	0.0	0.0	0.0	0.0	0.0
Crude Petroleum And Natural Gas & Metal Ores	-	-	-	3.8	3.6	-	-	-	0.7	-	-	-	-	-	-	-	-	-	-	-
Other mining and quarrying products	0.0	0.5	0.6	1.7	0.9	1.9	2.5	1.7	1.3	2.2	3.5	4.2	-	-	-	-	-	-	-	-
Mining support services	-	-	-	0.3	0.3	-	-	-	-	-	-	-	-	-	-	-	-	-	-	-
Preserved meat and meat products	0.0	-	-	-	0.0	0.6	0.2	0.6	0.4	1.1	0.2	4.7	3.3	4.0	4.3	3.0	4.8	5.0	4.5	4.3
Processed and preserved fish, crustaceans, molluscs, fruit and vegetables	9.0	10.0	9.7	9.2	7.6	9.7	10.8	9.0	10.5	14.2	19.8	28.5	-	-	-	-	-	-	-	-
Vegetable and animal oils and fats	1.0	0.8	0.1	0.8	0.6	1.0	1.2	1.0	0.7	1.2	1.5	4.3	3.8	5.4	4.4	3.2	4.1	3.8	3.2	3.0
Dairy products	0.6	1.0	1.4	1.3	0.8	1.0	0.9	0.7	0.7	1.1	1.7	2.5	1.5	1.4	0.9	12.9	0.8	0.7	0.7	0.7
Grain mill products, starches and starch products	-	-	-	-	-	-	-	-	-	-	-	-	-	-	-	-	-	-	-	-
Bakery and farinaceous products	1.4	1.8	2.1	2.0	1.7	2.3	2.5	2.4	2.5	4.5	6.3	10.1	7.4	8.7	8.3	7.3	9.7	9.3	8.4	8.2
Other food products	12.9	12.9	13.1	9.6	7.3	13.8	13.1	16.0	14.1	13.8	11.3	39.6	22.4	15.3	14.4	12.7	18.0	17.5	15.6	16.4
Prepared animal feeds	1.0	1.0	1.1	1.0	0.9	1.2	1.1	1.0	1.1	2.0	2.8	4.5	3.5	4.6	4.3	4.1	5.4	5.6	4.9	4.8
Alcoholic beverages	16.0	16.7	20.7	19.9	16.9	23.8	29.8	25.3	26.1	32.0	57.9	66.7	48.0	51.5	41.2	33.2	43.5	47.2	42.1	39.4
Soft drinks	6.4	7.5	9.3	9.3	8.5	12.0	13.3	12.3	13.2	16.1	29.0	32.7	20.2	24.3	19.8	15.7	20.5	23.0	20.6	19.1
Tobacco products	0.0	0.1	0.1	0.1	0.1	0.1	0.1	0.1	0.0	0.1	0.1	0.1	0.1	0.0	0.0	0.1	0.1	0.1	0.1	0.1
Textiles	0.0	-	-	-	-	-	-	-	-	-	-	-	-	-	-	-	-	-	-	0.9
Wearing apparel	-	-	-	-	-	-	-	-	-	-	-	-	-	-	-	-	-	-	-	-
Leather and related products	-	-	-	-	-	-	-	-	-	-	-	-	-	-	-	-	-	-	-	-
Wood and of products of wood and cork, except furniture; articles of straw and plaiting materials	0.9	1.0	1.3	1.2	0.9	1.1	1.3	0.8	0.8	1.2	2.1	2.4	3.0	3.2	2.9	2.6	4.2	5.1	4.8	4.7
Paper and paper products	13.7	20.0	21.4	19.1	18.5	25.5	27.6	16.2	15.7	20.3	43.1	51.6	55.6	61.3	60.1	47.3	64.3	65.8	63.1	64.1
Printing and recording services	14.6	17.3	20.1	18.9	15.8	22.4	22.6	16.0	16.4	27.4	49.0	74.9	51.9	46.4	49.8	44.8	74.1	74.4	71.8	73.2
Coke and refined petroleum products	3.0	2.9	5.4	8.9	8.6	13.7	18.8	15.4	18.0	27.0	46.7	56.8	64.5	75.2	85.9	96.9	98.6	106.6	98.9	94.8
Paints, varnishes and similar coatings, printing ink and mastics	10.8	6.8	17.5	38.3	69.1	55.0	44.9	66.4	81.8	97.5	114.2	136.9	144.1	157.1	170.1	183.8	178.9	186.7	198.1	202.6
Soap and detergents, cleaning and polishing preparations, perfumes and toilet preparations	20.8	14.7	34.7	67.7	124.5	97.6	80.9	122.3	152.7	183.4	215.9	229.7	253.0	286.3	320.0	355.3	354.1	377.2	398.5	419.2
Other chemical products	16.9	13.0	29.7	58.7	104.6	80.1	71.5	101.0	121.4	142.2	164.6	195.2	211.6	236.5	261.7	288.0	284.9	301.6	313.3	339.0
Industrial gases, inorganics and fertilisers (all inorganic chemicals) - 20.11/13/15	11.0	7.1	19.8	40.3	78.2	63.1	46.1	79.7	106.1	132.4	159.9	185.8	190.9	203.6	216.3	229.6	220.0	226.3	235.4	261.7
Petrochemicals - 20.14/16/17/60	43.2	30.4	78.3	157.8	293.4	237.0	201.1	328.3	426.1	523.6	626.2	746.4	754.5	792.8	830.5	870.4	824.1	838.3	863.0	874.5
Dyestuffs, agro-chemicals - 20.12/20	7.8	5.9	13.9	27.5	49.7	40.7	36.0	52.1	63.4	74.9	87.2	100.3	107.0	118.0	129.1	140.7	138.0	145.0	153.8	174.3
Basic pharmaceutical products and pharmaceutical preparations	43.0	31.8	79.4	165.3	332.0	285.5	252.3	356.0	427.4	500.4	579.1	809.8	860.1	944.7	1,029.8	1,119.1	1,094.9	1,147.9	1,215.0	1,395.3
Rubber and plastic products	7.3	11.2	12.4	10.4	8.5	11.2	14.5	11.9	12.3	19.3	37.3	45.1	65.9	64.5	62.6	63.2	91.7	102.1	106.5	113.6
Manufacture of cement, lime, plaster and articles of concrete, cement and plaster	0.0	0.1	0.1	0.1	0.1	0.0	0.1	0.0	0.0	0.1	0.2	0.2	0.1	0.2	0.2	0.2	0.3	0.3	0.3	0.3
Glass, refractory, clay, other porcelain and ceramic, stone and abrasive products - 23.1-4/7-9	3.2	3.9	4.7	4.8	3.9	6.1	5.7	3.3	2.7	4.7	7.3	12.6	10.8	11.4	10.6	8.6	10.9	10.6	8.3	6.2
Basic iron and steel	0.0	0.0	0.0	0.0	0.1	0.1	0.1	0.0	0.0	0.0	0.1	0.2	-	-	-	-	-	-	-	-
Other basic metals and casting	0.1	2.0	2.2	3.0	2.5	2.7	3.9	1.8	1.7	2.8	5.3	10.5	-	-	-	-	-	-	-	-
Weapons and ammunition	0.2	0.3	0.2	0.3	0.2	0.2	0.2	0.2	0.2	0.2	0.4	0.6	1.5	1.5	1.6	1.6	2.4	2.3	2.4	2.6
Fabricated metal products, excl. machinery and equipment and weapons & ammunition - 25.1-3/25.5-9	2.5	3.4	4.3	4.0	3.3	4.0	4.3	2.2	1.9	2.7	4.7	6.1	11.7	12.3	12.5	11.0	17.4	17.8	17.9	19.0

Sector – Rest-of-World	1997	1998	1999	2000	2001	2002	2003	2004	2005	2006	2007	2008	2009	2010	2011	2012	2013	2014	2015	2016
Computer, electronic and optical products	291.3	391.2	485.4	510.9	447.9	546.2	543.9	731.0	851.7	976.9	1,113.9	1,388.3	1,380.2	1,427.4	1,472.7	1,521.3	1,420.4	1,425.9	1,391.3	1,376.9
Electrical equipment	2.5	2.6	3.0	2.4	1.6	2.5	2.8	2.1	2.1	3.4	7.0	9.2	7.3	7.8	8.3	7.5	11.5	11.5	10.9	10.5
Machinery and equipment n.e.c.	0.4	0.5	0.8	0.8	0.7	0.8	0.8	0.6	0.7	1.1	2.0	2.7	7.9	7.9	7.4	7.4	10.6	13.7	14.2	15.1
Motor vehicles, trailers and semi-trailers	4.9	4.0	6.9	6.7	6.3	11.6	13.1	10.4	8.9	13.7	21.4	26.8	31.7	37.9	44.2	50.7	52.3	57.1	56.3	56.3
Ships and boats	1.5	1.8	2.1	1.4	1.1	1.0	1.1	0.8	1.0	1.6	3.9	3.9	4.3	2.9	4.3	3.6	5.1	10.5	10.2	8.9
Air and spacecraft and related machinery	7.0	9.3	13.3	9.4	7.7	6.8	8.4	6.4	7.6	11.1	25.1	22.8	27.1	18.4	28.0	21.5	29.2	59.0	58.7	62.2
Other transport equipment - 30.2/4/9	0.8	1.3	2.0	1.3	1.2	1.2	1.5	0.9	1.1	1.7	4.0	3.3	4.1	2.7	4.2	3.1	4.4	8.9	8.8	8.1
Furniture	-	-	-	-	-	-	-	-	-	-	-	3.8	-	-	0.3	0.8	-	-	2.3	7.1
Other manufactured goods	-	-	-	-	-	-	-	-	-	-	-	3.9	-	-	0.3	0.9	-	-	2.4	8.0
Repair and maintenance of ships and boats	0.4	0.5	0.6	0.4	0.4	0.3	0.3	0.2	0.4	0.4	1.1	1.1	1.1	0.8	1.1	1.2	1.5	3.4	4.1	3.2
Repair and maintenance of aircraft and spacecraft	1.3	1.9	2.9	2.2	1.8	1.4	1.9	1.3	1.6	2.3	5.3	5.5	5.4	3.8	4.7	3.9	5.7	12.4	12.2	13.4
Rest of repair; Installation - 33.11-14/17/19/20	1.1	0.8	0.8	1.0	0.6	0.3	0.3	0.3	0.3	0.3	0.4	0.6	0.5	0.7	0.4	0.4	0.5	0.5	0.4	0.4
Electricity, transmission and distribution	-	-	-	-	-	-	-	-	-	-	-	-	-	-	-	-	-	-	-	-
Gas; distribution of gaseous fuels through mains; steam and air conditioning supply	0.0	-	-	-	-	-	-	-	-	-	-	-	-	-	-	-	-	-	-	-
Natural water; water treatment and supply services	-	-	-	-	-	-	-	-	-	-	-	-	-	-	-	-	-	-	-	-
Sewerage services; sewage sludge	3.4	2.3	2.3	2.2	1.4	2.2	0.7	0.8	0.8	1.0	2.7	2.6	3.6	2.6	2.8	2.8	4.0	4.1	3.8	3.8
Waste collection, treatment and disposal services; materials recovery services	6.7	5.0	5.4	5.5	3.4	5.6	2.0	2.6	2.5	3.3	8.5	7.9	10.4	8.0	8.8	8.0	10.8	11.0	10.1	10.1
Remediation services and other waste management services	0.1	0.1	0.0	0.0	0.0	0.0	0.0	0.0	0.0	0.0	0.1	0.1	0.1	0.1	0.1	0.1	0.2	0.2	0.2	0.2
Construction	1.8	2.1	1.8	2.0	1.7	1.9	2.8	2.5	2.0	3.3	5.0	6.5	6.6	6.2	7.7	7.5	9.5	9.2	8.2	8.0
Wholesale and retail trade and repair services of motor vehicles and motorcycles	7.9	5.2	5.6	5.9	5.1	3.0	2.4	3.1	3.4	4.4	4.8	8.1	6.7	9.1	4.3	4.0	5.3	5.1	4.6	4.4
Wholesale trade services, except of motor vehicles and motorcycles	0.1	0.1	0.1	0.1	0.1	0.1	0.1	0.1	0.1	0.2	0.2	0.4	0.4	0.5	0.3	0.3	0.4	0.3	0.3	0.3
Retail trade services, except of motor vehicles and motorcycles	8.5	6.6	7.1	7.4	6.0	4.0	3.4	3.7	3.9	5.0	6.8	9.7	9.1	12.7	7.0	6.5	8.6	8.2	7.5	7.4
Rail transport services	2.6	2.2	2.5	2.6	1.7	4.1	5.5	3.8	3.9	5.7	8.9	10.3	6.9	9.0	7.6	7.6	10.0	10.2	9.2	9.1
Land transport services and transport services via pipelines, excluding rail transport	26.7	29.4	32.7	33.0	28.3	60.0	77.9	59.5	61.3	84.6	142.0	162.8	138.8	181.7	157.3	156.5	209.5	214.5	193.8	194.4
Water transport services	3.5	4.0	4.4	4.3	3.7	5.4	5.8	4.9	4.7	6.4	10.1	12.7	23.7	18.8	19.9	18.8	25.8	28.3	26.9	26.8
Air transport services	13.1	16.3	19.8	17.4	13.4	16.5	20.1	23.9	25.5	27.4	29.7	35.9	32.0	35.1	33.2	30.2	41.1	40.4	38.0	37.7
Warehousing and support services for transportation	3.2	3.6	4.2	4.3	2.8	5.5	7.7	4.4	5.1	7.1	11.6	14.9	8.3	9.4	8.9	8.6	10.7	10.8	9.6	9.3
Postal and courier services	9.0	10.6	11.7	10.5	7.6	10.2	12.4	8.0	9.6	16.3	31.8	34.5	20.2	20.1	20.1	18.0	22.3	21.5	18.4	17.1
Accommodation services	20.1	28.6	33.7	33.0	25.0	37.0	47.5	27.6	29.7	52.6	98.7	116.4	324.6	347.3	212.9	214.0	298.2	317.8	323.0	346.8
Food and beverage serving services	57.1	80.3	94.6	92.1	71.1	106.1	137.1	78.6	84.2	144.6	273.9	324.0	876.8	957.1	605.1	593.8	836.4	865.5	887.6	943.1
Publishing services	17.3	22.8	23.9	20.0	16.8	24.4	26.8	16.0	17.9	25.0	42.7	42.9	37.5	38.5	34.6	30.4	40.5	41.8	37.5	37.0
Motion Picture, Video & TV Programme Production, Sound Recording & Music Publishing Activities & Programming And Broadcasting Activities	2.2	2.4	2.5	2.4	2.5	3.3	4.3	3.4	3.8	5.7	10.9	13.8	9.2	9.8	10.0	9.5	12.3	11.1	10.4	10.3
Telecommunications services	22.1	28.5	32.9	31.9	22.4	28.5	35.1	23.8	29.1	47.2	93.5	100.2	52.4	50.9	48.8	43.1	53.9	53.8	47.1	43.4
Computer programming, consultancy and related services	2.9	4.1	4.9	4.9	5.1	6.7	9.3	7.4	8.1	13.3	25.3	31.4	19.6	19.8	21.9	20.3	26.9	25.6	23.2	22.8
Information services	0.5	0.6	0.6	0.6	0.7	0.9	1.2	0.9	1.0	1.7	3.3	4.3	2.6	2.9	2.9	2.8	3.7	3.5	3.1	3.0
Financial services, except insurance and pension funding	8.1	10.4	7.3	5.6	4.2	7.5	11.7	10.4	11.9	19.0	35.4	48.0	58.1	63.6	43.9	39.1	54.1	56.2	50.9	48.0
Insurance and reinsurance, except compulsory social security & Pension funding	9.3	9.8	10.5	11.2	6.4	10.4	10.5	13.4	14.4	15.7	29.3	30.4	26.8	29.6	27.5	25.4	32.3	29.5	23.8	20.4
Services auxiliary to financial services and insurance services	0.1	0.5	0.6	0.5	0.3	0.5	0.6	0.3	0.3	0.5	1.2	1.6	0.8	0.8	0.8	0.7	0.9	0.8	0.7	0.7

Sector – Rest-of-World	1997	1998	1999	2000	2001	2002	2003	2004	2005	2006	2007	2008	2009	2010	2011	2012	2013	2014	2015	2016
Real estate services, excluding on a fee or contract basis and imputed rent	2.3	2.1	2.3	2.3	1.5	2.5	2.8	1.4	2.0	4.5	13.6	13.9	10.3	12.2	10.6	11.1	15.2	15.3	13.8	13.6
Owner-Occupiers' Housing Services	8.2	6.7	7.4	7.3	4.8	7.8	8.9	4.7	6.7	13.7	39.2	38.3	26.6	29.7	24.2	23.2	31.1	30.9	28.0	27.5
Real estate activities on a fee or contract basis	0.2	0.2	0.2	0.2	0.2	0.3	0.3	0.1	0.2	0.6	1.8	1.7	1.3	1.5	1.2	1.1	1.4	1.5	1.4	1.4
Legal services	10.6	14.7	15.8	13.8	13.2	21.0	25.3	15.8	18.4	28.0	51.0	51.6	47.6	48.1	45.8	42.9	58.9	61.6	57.0	54.8
Accounting, bookkeeping and auditing services; tax consulting services	8.1	11.4	12.0	10.8	10.1	15.1	17.0	10.5	12.2	18.7	33.5	34.4	32.5	33.9	32.2	29.5	40.8	42.8	39.6	40.0
Services of head offices; management consulting services	9.0	13.0	14.7	13.0	12.5	19.6	23.5	14.6	18.1	29.3	54.8	61.0	55.5	57.1	53.7	47.7	67.7	71.9	69.3	68.4
Architectural and engineering services; technical testing and analysis services	18.5	25.1	25.9	21.6	19.4	31.0	36.2	22.8	28.3	43.0	78.3	81.6	73.8	75.2	69.9	65.8	90.4	93.2	85.4	86.5
Scientific research and development services	1.8	3.8	3.2	6.3	7.5	36.5	65.1	65.9	93.6	152.2	271.5	452.2	469.6	523.2	463.3	446.3	644.4	691.4	656.2	653.0
Advertising and market research services	5.5	8.0	9.1	8.2	8.1	13.0	14.4	8.7	9.7	14.3	26.5	28.5	25.9	27.9	26.4	23.9	33.4	37.0	34.8	33.6
Other professional, scientific and technical services	5.5	7.8	8.8	8.0	6.9	11.0	12.7	7.8	9.2	14.0	25.2	25.7	24.8	26.0	24.0	22.3	31.4	34.6	32.4	31.1
Veterinary services	1.5	1.9	2.1	1.7	1.6	2.4	2.8	1.7	2.2	3.0	5.7	5.7	5.6	5.8	5.5	5.3	7.6	8.5	8.0	8.5
Rental and leasing services	7.2	10.1	10.9	10.1	4.7	8.1	9.2	7.7	7.9	10.7	20.6	22.7	26.0	28.7	30.3	28.6	39.0	39.2	35.8	35.5
Employment services	11.4	17.1	18.9	17.0	15.2	22.9	27.1	16.6	19.0	28.7	51.7	57.3	44.4	45.9	42.1	38.2	55.5	62.9	62.6	54.4
Travel agency, tour operator and other reservation services and related services	8.5	11.9	12.4	10.5	9.3	14.3	16.9	10.4	12.2	17.8	31.6	33.1	27.7	26.4	25.3	23.9	32.8	34.2	30.6	33.2
Security and investigation services	2.0	3.0	3.3	3.0	2.7	4.0	4.7	2.9	3.3	5.0	8.9	9.2	8.6	8.9	8.3	7.5	9.9	10.4	9.7	9.4
Services to buildings and landscape	6.5	8.9	9.3	7.8	6.9	10.7	12.5	7.8	8.8	13.5	24.8	26.7	26.6	28.8	27.7	25.8	35.4	36.5	34.2	33.2
Office administrative, office support and other business support services	11.2	16.3	18.7	16.9	14.6	22.0	25.5	15.7	18.3	27.7	49.9	54.5	49.6	53.8	52.9	49.0	67.6	70.5	64.1	62.4
Public administration and defence services; compulsory social security services	1.7	9.7	10.5	9.8	6.8	7.5	11.5	7.8	7.2	10.9	21.1	28.2	24.1	22.8	18.6	16.0	20.8	20.2	18.6	17.6
Education services	7.7	8.3	8.7	9.0	4.2	2.4	2.7	5.2	7.2	13.3	13.9	24.5	11.1	15.3	56.6	56.2	72.8	65.5	51.0	46.5
Human health services	75.0	97.2	116.3	117.6	74.6	41.2	49.3	41.6	59.1	141.3	122.9	253.1	254.0	364.8	1,404.9	1,454.7	1,999.1	1,860.8	1,507.0	1,471.3
Residential Care & Social Work Activities	34.3	44.7	55.5	55.7	34.8	19.7	23.9	19.7	27.8	64.2	56.0	107.1	108.6	146.3	563.8	614.6	836.3	775.2	634.3	593.8
Creative, arts and entertainment services	0.3	0.3	0.4	0.4	0.3	0.4	0.3	0.3	0.3	0.4	0.7	1.1	0.3	0.3	-	-	-	-	-	-
Libraries, archives, museums and other cultural services	0.2	0.2	0.3	0.3	0.2	0.3	0.2	0.2	0.2	0.3	0.4	0.7	0.2	0.2	-	-	-	-	-	-
Gambling and betting services	0.5	0.6	0.7	0.7	0.5	0.8	0.7	0.7	0.6	0.8	1.4	2.0	0.5	0.5	-	-	-	-	-	-
Sports services and amusement and recreation services	0.4	0.5	0.6	0.7	0.4	0.6	0.6	0.6	0.5	0.6	1.1	1.5	0.4	0.4	-	-	-	-	-	-
Services furnished by membership organisations	-	-	-	-	-	-	-	-	-	-	7.1	9.0	-	-	-	-	-	-	-	-
Repair services of computers and personal and household goods	0.4	0.4	0.5	0.6	0.2	0.3	0.3	0.4	0.6	1.1	1.0	1.8	1.4	1.3	2.1	2.2	3.1	3.2	2.9	2.9
Other personal services	2.3	2.0	2.5	2.6	0.9	2.0	1.9	3.0	4.3	7.5	7.2	12.5	8.4	9.3	13.4	13.6	18.2	18.0	15.2	14.6
Services of households as employers of domestic personnel	-	-	-	-	-	-	-	-	-	-	-	-	-	-	-	-	-	-	-	-

Table S6. UK-MRIO model GHG emission factors, CO_2e tonnes / £ thousands

Sector – UK	1997	1998	1999	2000	2001	2002	2003	2004	2005	2006	2007	2008	2009	2010	2011	2012	2013	2014	2015	2016
Products of agriculture, hunting and related services	3.75	3.97	4.07	4.02	3.99	3.85	3.65	3.51	4.01	3.71	3.33	2.97	2.95	2.75	2.73	2.69	2.47	2.56	2.58	2.63
Products of forestry, logging and related services	0.65	0.59	0.61	0.56	0.49	0.51	0.54	0.51	0.51	0.48	0.50	0.48	0.51	0.54	0.66	0.61	0.42	0.42	0.37	0.39
Fish and other fishing products; aquaculture products; support services to fishing	1.95	2.02	1.90	1.78	1.92	1.83	1.87	1.91	1.69	1.46	1.45	1.32	1.18	1.06	1.10	1.07	1.06	1.00	1.00	0.85
Coal and lignite	8.48	8.39	9.94	8.26	7.04	8.78	9.19	10.90	11.71	9.14	8.07	4.94	5.68	2.28	3.33	3.44	3.55	3.12	3.12	2.09
Crude Petroleum And Natural Gas & Metal Ores	1.43	1.58	1.49	1.11	1.16	1.24	1.19	1.20	0.98	0.78	0.78	0.66	0.64	0.61	0.57	0.58	0.56	0.63	0.81	0.87
Other mining and quarrying products	1.10	1.37	1.53	1.30	1.06	0.79	0.78	0.82	0.95	0.77	0.70	0.68	2.46	2.74	2.71	2.37	1.97	1.83	1.81	1.70
Mining support services	1.15	1.34	1.25	0.94	0.94	1.03	0.95	0.97	0.78	0.62	0.63	0.52	0.52	0.52	0.43	0.50	0.43	0.50	0.61	0.68
Preserved meat and meat products	1.56	1.69	1.58	1.64	1.67	1.64	1.64	1.65	1.72	1.67	1.59	1.37	1.23	1.22	1.15	1.08	1.04	1.06	1.07	0.94
Processed and preserved fish, crustaceans, molluscs, fruit and vegetables	1.33	1.30	1.13	1.12	1.12	1.11	1.05	1.06	1.06	1.01	1.01	0.92	0.33	0.28	0.23	0.22	0.23	0.24	0.24	0.23
Vegetable and animal oils and fats	0.63	0.63	0.74	0.63	0.56	0.61	0.27	0.30	0.30	0.27	0.32	0.29	0.27	0.26	0.29	0.28	0.25	0.27	0.28	0.26
Dairy products	1.85	1.82	1.84	1.82	1.75	1.72	1.62	1.83	2.13	2.04	1.95	1.91	1.40	1.35	1.30	1.23	1.22	1.24	1.13	1.18
Grain mill products, starches and starch products	1.17	1.07	1.00	0.96	0.95	0.96	0.93	0.99	0.90	0.96	0.86	0.70	0.39	0.36	0.34	0.32	0.33	0.35	0.34	0.29
Bakery and farinaceous products	1.37	1.30	1.24	1.21	1.19	1.14	1.06	1.13	1.18	1.13	1.11	0.97	0.90	0.88	0.83	0.81	0.78	0.75	0.74	0.67
Other food products	1.69	1.59	1.53	1.46	1.41	1.36	1.37	1.46	1.56	1.47	1.47	1.28	1.18	1.12	1.09	1.06	1.02	1.01	0.99	0.91
Prepared animal feeds	1.42	1.38	1.31	1.24	1.28	1.24	1.09	1.22	1.33	1.28	1.25	1.09	1.02	1.03	0.97	0.94	0.92	0.91	0.90	0.83
Alcoholic beverages	0.74	0.69	0.62	0.60	0.63	0.62	0.56	0.58	0.60	0.60	0.62	0.57	0.19	0.20	0.16	0.14	0.14	0.16	0.17	0.15
Soft drinks	0.71	0.67	0.59	0.58	0.61	0.59	0.52	0.54	0.56	0.58	0.57	0.52	0.18	0.18	0.14	0.13	0.14	0.15	0.17	0.15
Tobacco products	0.84	0.63	0.50	0.61	0.66	0.63	0.71	1.07	0.81	1.11	0.95	0.91	0.32	0.67	0.60	0.53	0.45	0.43	0.51	0.46
Textiles	1.10	1.19	1.16	1.23	1.19	1.18	1.19	1.13	1.22	1.16	1.06	1.01	0.84	0.74	0.74	0.68	0.63	0.61	0.62	0.56
Wearing apparel	0.46	0.53	0.52	0.59	0.58	0.67	0.72	0.74	0.80	0.72	0.67	0.58	0.67	0.58	0.50	0.48	0.36	0.37	0.36	0.30
Leather and related products	0.73	0.73	0.76	0.91	0.81	0.92	0.83	0.87	0.75	0.78	0.69	0.55	0.35	0.73	0.32	0.31	0.27	0.28	0.27	0.24
Wood and of products of wood and cork, except furniture; articles of straw and plaiting materials	1.49	1.29	1.43	1.42	1.10	1.01	1.03	1.03	0.97	0.94	0.86	0.75	0.83	0.80	0.80	0.76	0.70	0.69	0.67	0.61
Paper and paper products	1.22	1.25	1.18	1.35	1.19	1.15	1.21	1.31	1.41	1.40	1.32	1.13	0.92	0.91	0.89	0.87	0.79	0.77	0.79	0.68
Printing and recording services	0.77	0.76	0.80	0.78	0.84	0.84	0.98	0.81	0.83	0.70	0.70	0.64	0.61	0.55	0.53	0.53	0.51	0.49	0.49	0.45
Coke and refined petroleum products	3.68	4.04	3.61	3.09	3.31	3.48	3.50	3.08	2.96	2.54	2.52	2.03	1.99	1.87	1.64	1.56	1.61	1.65	1.90	1.91
Paints, varnishes and similar coatings, printing ink and mastics	2.21	1.95	1.42	1.44	1.32	1.20	1.15	1.23	1.19	1.11	1.09	0.89	0.78	0.76	0.76	0.76	0.68	0.64	0.62	0.57
Soap and detergents, cleaning and polishing preparations, perfumes and toilet preparations	1.78	1.72	1.19	1.20	1.11	0.98	0.92	0.95	0.88	0.85	0.82	0.66	0.58	0.57	0.59	0.57	0.54	0.53	0.52	0.50
Other chemical products	1.94	1.99	1.23	1.30	1.29	1.18	1.08	1.03	1.02	0.96	1.00	0.89	0.79	0.83	0.79	0.75	0.72	0.66	0.65	0.58
Industrial gases, inorganics and fertilisers (all inorganic chemicals) - 20.11/13/15	1.75	1.77	1.39	1.49	1.43	1.32	1.13	1.32	1.20	1.20	1.07	0.87	0.76	0.74	0.77	0.74	0.68	0.70	0.71	0.64
Petrochemicals - 20.14/16/17/60	2.22	2.30	1.55	1.42	1.25	1.12	1.08	1.17	1.08	1.00	1.02	0.84	0.72	0.70	0.71	0.71	0.66	0.63	0.60	0.56
Dyestuffs, agro-chemicals - 20.12/20	1.80	1.69	1.13	1.18	1.11	1.08	1.15	1.29	1.17	1.13	1.17	0.95	0.81	0.79	0.79	0.79	0.73	0.74	0.73	0.68
Basic pharmaceutical products and pharmaceutical preparations	2.42	2.27	1.64	1.66	1.56	1.42	1.28	1.39	1.24	1.08	1.05	0.85	0.72	0.70	0.69	0.68	0.64	0.61	0.60	0.57
Rubber and plastic products	1.12	1.04	0.95	0.94	0.95	0.88	0.93	0.98	1.03	1.01	0.95	0.81	0.64	0.69	0.66	0.62	0.62	0.61	0.61	0.55
Manufacture of cement, lime, plaster and articles of concrete, cement and plaster	4.36	4.43	4.13	3.90	3.62	3.55	3.25	3.56	3.26	3.17	2.99	2.58	2.39	2.30	2.32	2.26	2.29	2.26	2.08	1.82
Glass, refractory, clay, other porcelain and ceramic, stone and abrasive products - 23.1-4/7-9	1.31	1.31	1.24	1.21	1.15	1.15	1.19	1.08	1.12	1.02	1.02	1.01	0.90	0.84	0.77	0.71	0.71	0.72	0.70	0.63
Basic iron and steel	4.32	4.14	4.89	4.95	4.77	4.73	4.36	4.63	4.01	3.81	3.72	3.43	2.96	2.55	2.36	2.46	2.66	2.69	2.56	2.01
Other basic metals and casting	3.31	3.34	3.39	3.01	3.10	3.15	3.27	3.08	2.80	2.56	2.13	2.03	1.84	1.87	1.59	1.44	1.24	1.19	1.06	1.04
Weapons and ammunition	1.20	1.13	1.02	0.94	0.89	0.89	0.76	0.92	0.80	0.78	0.75	0.69	0.43	0.43	0.39	0.39	0.36	0.36	0.36	0.34

Sector – UK	1997	1998	1999	2000	2001	2002	2003	2004	2005	2006	2007	2008	2009	2010	2011	2012	2013	2014	2015	2016
Fabricated metal products, excl. machinery and equipment and weapons & ammunition - 25.1-3/25.5-9	1.37	1.28	1.32	1.11	1.12	1.09	1.12	1.12	1.04	1.00	0.92	0.77	0.48	0.48	0.44	0.43	0.39	0.40	0.41	0.38
Computer, electronic and optical products	0.83	0.84	0.80	0.76	0.82	0.90	0.98	1.04	0.89	0.92	0.87	0.69	0.95	0.90	0.89	0.87	0.82	0.85	0.93	0.87
Electrical equipment	0.88	0.89	0.81	0.80	0.78	0.81	0.79	0.82	0.81	0.81	0.77	0.65	0.74	0.69	0.69	0.69	0.68	0.72	0.73	0.66
Machinery and equipment n.e.c.	1.35	1.35	1.28	1.25	1.27	1.20	1.24	1.20	1.21	1.15	1.07	0.92	0.67	0.62	0.57	0.55	0.48	0.48	0.49	0.44
Motor vehicles, trailers and semi-trailers	1.11	1.23	1.20	1.13	1.22	1.16	1.21	1.23	1.21	1.11	1.04	0.85	0.66	0.60	0.63	0.63	0.61	0.57	0.56	0.46
Ships and boats	0.65	0.64	0.57	0.52	0.52	0.54	0.56	0.54	0.52	0.52	0.53	0.49	0.38	0.38	0.29	0.31	0.32	0.30	0.28	0.27
Air and spacecraft and related machinery	0.84	0.88	0.75	0.70	0.71	0.73	0.68	0.76	0.80	0.73	0.75	0.68	0.51	0.48	0.37	0.41	0.41	0.40	0.40	0.35
Other transport equipment - 30.2/4/9	0.82	0.81	0.68	0.69	0.72	0.71	0.69	0.73	0.80	0.72	0.73	0.67	0.53	0.47	0.38	0.44	0.41	0.41	0.42	0.38
Furniture	1.03	0.95	0.96	0.92	0.92	0.84	0.92	0.87	0.82	0.80	0.72	0.66	0.79	0.78	0.72	0.73	0.66	0.66	0.64	0.59
Other manufactured goods	0.89	0.83	0.82	0.78	0.82	0.78	1.62	1.54	1.47	1.36	1.29	1.12	0.79	0.77	0.74	0.74	0.67	0.65	0.65	0.61
Repair and maintenance of ships and boats	0.81	1.00	0.81	0.93	1.04	0.67	0.48	0.80	0.83	0.55	0.60	0.60	0.48	0.39	0.36	0.44	0.42	0.50	0.64	0.44
Repair and maintenance of aircraft and spacecraft	0.61	0.77	0.75	0.71	0.72	0.61	0.62	0.64	0.71	0.64	0.65	0.61	0.47	0.44	0.35	0.41	0.42	0.45	0.45	0.45
Rest of repair; Installation - 33.11-14/17/19/20	0.01	0.01	0.01	0.02	0.01	0.01	0.01	0.01	0.00	0.00	0.00	0.00	0.00	0.00	0.00	0.00	0.00	0.00	0.00	0.00
Electricity, transmission and distribution	9.20	9.36	9.20	9.24	9.81	9.49	9.35	9.06	8.41	8.03	7.84	7.13	5.56	5.58	5.50	5.35	5.13	4.81	4.47	3.69
Gas; distribution of gaseous fuels through mains; steam and air conditioning supply	2.01	1.99	1.79	1.85	1.89	1.97	2.18	1.85	1.56	1.35	1.38	1.40	1.13	1.04	0.97	0.88	0.93	0.91	0.88	0.77
Natural water; water treatment and supply services	0.46	0.58	0.65	0.75	0.77	0.62	0.59	0.73	0.78	0.74	0.65	0.57	0.49	0.49	0.46	0.46	0.44	0.43	0.45	0.39
Sewerage services; sewage sludge	7.06	6.56	5.96	5.55	5.03	4.48	3.61	3.10	2.82	2.53	2.26	2.01	1.82	1.53	1.36	1.34	1.29	1.18	1.18	1.19
Waste collection, treatment and disposal services; materials recovery services	7.79	7.13	6.58	6.13	5.52	4.88	3.92	3.35	3.03	2.69	2.43	2.17	1.96	1.65	1.47	1.47	1.42	1.31	1.32	1.34
Remediation services and other waste management services	6.90	6.63	6.05	5.62	5.01	4.43	3.60	2.62	2.83	1.98	2.27	1.72	1.81	1.44	1.36	1.34	1.29	1.18	1.18	1.18
Construction	0.68	0.63	0.60	0.54	0.53	0.47	0.48	0.48	0.45	0.41	0.41	0.39	0.37	0.37	0.35	0.36	0.34	0.32	0.34	0.31
Wholesale and retail trade and repair services of motor vehicles and motorcycles	0.63	0.71	0.62	0.59	0.49	0.43	0.40	0.40	0.36	0.32	0.31	0.29	0.26	0.23	0.22	0.23	0.22	0.20	0.20	0.17
Wholesale trade services, except of motor vehicles and motorcycles	0.00	0.00	0.00	0.00	0.00	0.00	0.00	0.00	0.00	0.00	0.00	0.00	0.00	0.00	0.00	0.00	0.00	0.00	0.00	0.00
Retail trade services, except of motor vehicles and motorcycles	0.00	0.00	0.00	0.00	0.00	0.00	0.00	0.00	0.00	0.00	0.00	0.00	0.00	0.00	0.00	0.00	0.00	0.00	0.00	0.00
Rail transport services	1.08	1.06	0.94	0.89	0.88	0.83	0.85	0.79	0.78	0.71	0.71	0.66	0.62	0.57	0.55	0.54	0.52	0.50	0.48	0.44
Land transport services and transport services via pipelines, excluding rail transport	1.19	1.17	1.09	1.02	1.00	1.01	0.98	0.94	0.91	0.85	0.82	0.76	0.73	0.68	0.64	0.64	0.60	0.56	0.55	0.52
Water transport services	5.89	6.30	5.94	5.79	6.13	5.99	4.97	4.50	4.05	3.13	2.75	2.32	2.16	2.01	1.94	1.55	1.24	1.34	1.48	1.44
Air transport services	2.99	3.13	3.18	3.24	3.32	3.31	3.20	3.25	3.33	3.13	2.94	2.80	2.84	2.86	2.90	2.72	2.55	2.33	2.30	2.24
Warehousing and support services for transportation	0.43	0.42	0.39	0.37	0.36	0.34	0.33	0.35	0.35	0.34	0.32	0.27	0.29	0.27	0.25	0.24	0.22	0.22	0.22	0.20
Postal and courier services	0.42	0.43	0.38	0.35	0.36	0.35	0.33	0.33	0.32	0.30	0.27	0.24	0.21	0.22	0.22	0.22	0.21	0.20	0.20	0.18
Accommodation services	0.57	0.55	0.49	0.48	0.47	0.44	0.42	0.44	0.44	0.41	0.39	0.37	0.31	0.30	0.28	0.27	0.25	0.23	0.24	0.21
Food and beverage serving services	0.52	0.49	0.43	0.42	0.41	0.39	0.37	0.39	0.39	0.36	0.35	0.32	0.27	0.27	0.24	0.24	0.22	0.20	0.21	0.19
Publishing services	0.48	0.50	0.45	0.42	0.42	0.40	0.39	0.35	0.34	0.30	0.29	0.26	0.24	0.23	0.21	0.21	0.19	0.17	0.17	0.15
Motion Picture, Video & TV Programme Production, Sound Recording & Music Publishing Activities & Programming And Broadcasting Activities	0.31	0.30	0.26	0.26	0.25	0.23	0.23	0.25	0.24	0.23	0.21	0.19	0.16	0.16	0.15	0.15	0.15	0.14	0.14	0.13
Telecommunications services	0.44	0.45	0.40	0.37	0.38	0.36	0.34	0.35	0.34	0.32	0.29	0.26	0.23	0.24	0.23	0.23	0.22	0.21	0.21	0.18
Computer programming, consultancy and related services	0.20	0.22	0.20	0.20	0.19	0.18	0.17	0.19	0.18	0.17	0.16	0.15	0.12	0.13	0.11	0.12	0.11	0.10	0.11	0.10
Information services	0.24	0.24	0.22	0.23	0.21	0.20	0.20	0.21	0.20	0.19	0.18	0.17	0.13	0.14	0.13	0.13	0.13	0.12	0.12	0.11
Financial services, except insurance and pension funding	0.26	0.27	0.27	0.26	0.25	0.21	0.19	0.17	0.17	0.13	0.12	0.12	0.09	0.10	0.10	0.10	0.10	0.10	0.10	0.09
Insurance and reinsurance, except compulsory social security & Pension funding	0.23	0.24	0.26	0.26	0.26	0.26	0.24	0.21	0.18	0.18	0.15	0.15	0.13	0.14	0.14	0.12	0.11	0.10	0.12	0.10
Services auxiliary to financial services and insurance services	0.31	0.31	0.26	0.24	0.23	0.21	0.20	0.20	0.18	0.14	0.13	0.09	0.09	0.09	0.09	0.09	0.08	0.08	0.07	0.06

Sector – UK	1997	1998	1999	2000	2001	2002	2003	2004	2005	2006	2007	2008	2009	2010	2011	2012	2013	2014	2015	2016
Real estate services, excluding on a fee or contract basis and imputed rent	0.06	0.07	0.07	0.06	0.06	0.06	0.06	0.05	0.05	0.05	0.05	0.04	0.03	0.03	0.03	0.03	0.03	0.03	0.03	0.03
Owner-Occupiers' Housing Services	0.00	0.00	0.00	0.00	0.00	0.00	0.00	0.00	0.00	0.00	0.00	0.00	0.00	0.00	0.00	0.00	0.00	0.00	0.00	0.00
Real estate activities on a fee or contract basis	0.09	0.09	0.09	0.09	0.08	0.08	0.08	0.07	0.07	0.06	0.06	0.06	0.04	0.04	0.04	0.04	0.04	0.04	0.04	0.04
Legal services	0.34	0.33	0.30	0.28	0.28	0.27	0.26	0.23	0.23	0.20	0.19	0.18	0.16	0.16	0.15	0.15	0.13	0.12	0.12	0.10
Accounting, bookkeeping and auditing services; tax consulting services	0.48	0.49	0.44	0.41	0.40	0.38	0.36	0.32	0.32	0.28	0.26	0.24	0.22	0.21	0.19	0.19	0.18	0.16	0.16	0.13
Services of head offices; management consulting services	0.24	0.24	0.22	0.21	0.22	0.21	0.21	0.19	0.18	0.17	0.16	0.15	0.14	0.14	0.13	0.12	0.11	0.10	0.10	0.09
Architectural and engineering services; technical testing and analysis services	0.37	0.36	0.32	0.30	0.29	0.28	0.28	0.25	0.24	0.22	0.21	0.19	0.18	0.17	0.16	0.16	0.15	0.13	0.13	0.11
Scientific research and development services	0.25	0.25	0.19	0.19	0.19	0.24	0.21	0.20	0.19	0.15	0.14	0.14	0.47	0.45	0.41	0.46	0.42	0.33	0.29	0.23
Advertising and market research services	0.14	0.15	0.13	0.13	0.14	0.14	0.13	0.12	0.12	0.11	0.11	0.10	0.10	0.09	0.09	0.09	0.08	0.08	0.08	0.07
Other professional, scientific and technical services	0.27	0.28	0.25	0.24	0.23	0.23	0.22	0.20	0.20	0.18	0.17	0.16	0.15	0.14	0.13	0.13	0.12	0.11	0.11	0.09
Veterinary services	0.34	0.34	0.30	0.28	0.28	0.27	0.26	0.23	0.23	0.20	0.19	0.18	0.16	0.16	0.15	0.15	0.13	0.12	0.12	0.11
Rental and leasing services	0.60	0.57	0.50	0.47	0.46	0.44	0.42	0.40	0.38	0.35	0.33	0.30	0.27	0.26	0.24	0.25	0.23	0.21	0.21	0.18
Employment services	0.28	0.28	0.25	0.24	0.23	0.22	0.22	0.20	0.19	0.18	0.16	0.16	0.14	0.13	0.12	0.12	0.11	0.10	0.10	0.09
Travel agency, tour operator and other reservation services and related services	0.35	0.35	0.31	0.29	0.29	0.28	0.27	0.24	0.24	0.21	0.20	0.18	0.17	0.17	0.15	0.15	0.14	0.12	0.12	0.11
Security and investigation services	0.28	0.28	0.25	0.24	0.23	0.22	0.21	0.20	0.19	0.17	0.16	0.15	0.14	0.13	0.12	0.12	0.11	0.10	0.10	0.09
Services to buildings and landscape	0.34	0.34	0.31	0.29	0.29	0.28	0.27	0.25	0.24	0.22	0.21	0.19	0.18	0.18	0.16	0.16	0.15	0.13	0.13	0.11
Office administrative, office support and other business support services	0.28	0.29	0.26	0.25	0.23	0.22	0.22	0.20	0.19	0.17	0.16	0.15	0.14	0.14	0.13	0.13	0.12	0.11	0.10	0.09
Public administration and defence services; compulsory social security services	0.40	0.40	0.38	0.37	0.36	0.34	0.33	0.33	0.30	0.28	0.28	0.25	0.22	0.21	0.20	0.19	0.17	0.17	0.17	0.15
Education services	0.31	0.28	0.25	0.22	0.20	0.19	0.17	0.16	0.15	0.15	0.15	0.14	0.12	0.12	0.11	0.11	0.11	0.10	0.10	0.09
Human health services	0.33	0.31	0.28	0.26	0.25	0.22	0.21	0.22	0.22	0.20	0.20	0.20	0.19	0.19	0.18	0.18	0.17	0.16	0.16	0.14
Residential Care & Social Work Activities	0.33	0.31	0.28	0.26	0.24	0.22	0.21	0.22	0.22	0.20	0.20	0.20	0.19	0.19	0.18	0.18	0.17	0.16	0.16	0.14
Creative, arts and entertainment services	0.50	0.48	0.41	0.39	0.38	0.36	0.35	0.34	0.33	0.31	0.29	0.26	0.20	0.19	0.18	0.18	0.17	0.16	0.16	0.14
Libraries, archives, museums and other cultural services	0.32	0.30	0.25	0.26	0.24	0.21	0.21	0.21	0.22	0.21	0.18	0.17	0.15	0.12	0.11	0.11	0.10	0.10	0.09	0.10
Gambling and betting services	0.65	0.65	0.54	0.53	0.51	0.47	0.45	0.44	0.44	0.41	0.38	0.33	0.27	0.26	0.24	0.23	0.22	0.20	0.19	0.17
Sports services and amusement and recreation services	0.55	0.53	0.45	0.42	0.42	0.39	0.38	0.37	0.36	0.34	0.32	0.28	0.22	0.21	0.20	0.20	0.19	0.18	0.17	0.15
Services furnished by membership organisations	0.23	0.21	0.20	0.20	0.19	0.17	0.17	0.17	0.18	0.16	0.15	0.14	0.12	0.10	0.10	0.10	0.10	0.09	0.10	0.09
Repair services of computers and personal and household goods	0.35	0.36	0.34	0.35	0.27	0.23	0.19	0.18	0.16	0.14	0.12	0.11	0.09	0.07	0.07	0.08	0.07	0.07	0.08	0.07
Other personal services	0.40	0.39	0.36	0.34	0.32	0.28	0.27	0.26	0.25	0.22	0.19	0.17	0.13	0.11	0.11	0.11	0.11	0.10	0.10	0.09
Services of households as employers of domestic personnel	0.00	0.00	0.00	0.00	0.00	0.00	0.00	0.00	0.00	0.00	0.00	0.00	0.00	0.00	0.00	0.00	0.00	0.00	0.00	0.00

Sector – EU	1997	1998	1999	2000	2001	2002	2003	2004	2005	2006	2007	2008	2009	2010	2011	2012	2013	2014	2015	2016
Products of agriculture, hunting and related services	3.28	3.31	3.35	3.49	3.19	3.18	2.85	2.73	2.79	2.74	2.51	2.04	1.96	1.90	1.72	1.56	1.44	1.52	1.48	1.44
Products of forestry, logging and related services	0.67	0.65	0.50	0.48	0.44	0.47	0.55	0.56	0.58	0.53	0.46	0.43	0.40	0.39	0.34	0.36	0.35	0.38	0.38	0.38
Fish and other fishing products; aquaculture products; support services to fishing	1.09	1.02	1.00	1.01	0.90	0.91	0.96	1.02	1.04	1.02	1.02	0.95	0.81	0.77	0.78	0.80	0.77	0.81	0.81	0.82
Coal and lignite	16.16	17.06	14.62	14.56	13.93	12.82	13.18	12.00	9.76	9.74	8.64	5.15	4.88	4.84	4.33	4.35	4.15	4.31	4.22	4.14
Crude Petroleum And Natural Gas & Metal Ores	2.50	2.67	2.70	1.94	1.58	1.90	1.82	1.90	1.80	1.61	1.58	1.19	1.09	1.02	0.97	0.99	0.98	1.05	1.07	1.08
Other mining and quarrying products	0.73	0.72	0.73	0.65	0.60	0.56	0.54	0.56	0.53	0.55	0.48	0.41	0.36	0.35	0.35	0.35	0.32	0.33	0.33	0.33
Mining support services	3.08	3.18	3.08	2.06	1.63	2.07	1.94	2.01	1.92	1.77	1.74	1.25	1.16	1.14	1.09	1.09	1.08	1.11	1.11	1.12
Preserved meat and meat products	1.58	1.54	1.49	1.56	1.49	1.40	1.22	1.20	1.17	1.14	1.07	0.91	0.77	0.78	0.74	0.71	0.65	0.67	0.66	0.65

Sector – EU	1997	1998	1999	2000	2001	2002	2003	2004	2005	2006	2007	2008	2009	2010	2011	2012	2013	2014	2015	2016
Processed and preserved fish, crustaceans, molluscs, fruit and vegetables	2.06	2.01	1.94	2.02	1.87	1.82	1.64	1.74	1.54	1.45	1.40	1.21	1.07	1.15	0.96	0.90	0.83	0.85	0.84	0.82
Vegetable and animal oils and fats	1.59	1.61	1.50	1.51	1.44	1.41	1.28	1.24	1.17	1.07	1.06	0.99	0.79	0.81	0.82	0.80	0.74	0.73	0.72	0.71
Dairy products	1.84	1.80	1.72	1.80	1.69	1.63	1.43	1.40	1.37	1.31	1.28	1.06	0.89	0.90	0.87	0.82	0.76	0.78	0.77	0.76
Grain mill products, starches and starch products	0.80	0.79	0.82	0.81	0.75	0.73	0.63	0.60	0.54	0.54	0.51	0.45	0.39	0.40	0.40	0.40	0.37	0.38	0.38	0.38
Bakery and farinaceous products	1.45	1.42	1.36	1.41	1.33	1.27	1.12	1.11	1.08	1.05	1.03	0.89	0.72	0.74	0.73	0.70	0.65	0.67	0.66	0.66
Other food products	1.44	1.41	1.36	1.40	1.33	1.27	1.12	1.11	1.07	1.05	1.03	0.89	0.72	0.74	0.73	0.70	0.65	0.67	0.66	0.65
Prepared animal feeds	1.45	1.42	1.36	1.41	1.33	1.27	1.12	1.11	1.08	1.05	1.03	0.89	0.72	0.74	0.73	0.70	0.65	0.67	0.66	0.66
Alcoholic beverages	1.57	1.52	1.49	1.54	1.43	1.38	1.23	1.23	1.18	1.17	1.16	0.97	0.80	0.84	0.80	0.77	0.71	0.72	0.72	0.71
Soft drinks	1.57	1.52	1.49	1.54	1.43	1.38	1.23	1.23	1.18	1.17	1.16	0.97	0.80	0.84	0.80	0.77	0.71	0.72	0.72	0.71
Tobacco products	0.85	0.84	0.75	0.67	0.61	0.61	0.59	0.56	0.56	0.49	0.65	0.58	0.51	0.54	0.51	0.50	0.49	0.51	0.50	0.50
Textiles	0.85	0.85	0.79	0.84	0.80	0.78	0.72	0.74	0.73	0.70	0.66	0.55	0.43	0.46	0.45	0.45	0.42	0.43	0.44	0.44
Wearing apparel	0.62	0.63	0.61	0.63	0.60	0.58	0.56	0.56	0.55	0.53	0.51	0.42	0.37	0.38	0.37	0.37	0.35	0.36	0.36	0.36
Leather and related products	0.60	0.62	0.60	0.69	0.63	0.61	0.57	0.56	0.54	0.54	0.51	0.42	0.34	0.35	0.34	0.35	0.32	0.34	0.34	0.34
Wood and of products of wood and cork, except furniture; articles of straw and plaiting materials	0.56	0.59	0.54	0.57	0.55	0.56	0.54	0.56	0.57	0.55	0.49	0.45	0.38	0.38	0.38	0.40	0.38	0.39	0.40	0.40
Paper and paper products	0.92	0.90	0.85	0.81	0.74	0.75	0.72	0.71	0.71	0.69	0.64	0.55	0.47	0.49	0.44	0.46	0.43	0.45	0.45	0.45
Printing and recording services	0.46	0.46	0.44	0.45	0.43	0.42	0.40	0.38	0.37	0.37	0.35	0.29	0.24	0.22	0.21	0.22	0.20	0.21	0.21	0.21
Coke and refined petroleum products	3.42	3.50	3.60	2.97	2.78	2.89	2.69	2.54	2.36	2.16	2.10	1.70	1.77	1.61	1.43	1.42	1.33	1.39	1.37	1.35
Paints, varnishes and similar coatings, printing ink and mastics	0.58	0.55	0.56	0.59	0.55	0.51	0.49	0.50	0.53	0.53	0.53	0.44	0.34	0.37	0.35	0.37	0.33	0.34	0.34	0.33
Soap and detergents, cleaning and polishing preparations, perfumes and toilet preparations	0.47	0.48	0.47	0.49	0.46	0.42	0.39	0.39	0.39	0.41	0.40	0.33	0.25	0.28	0.27	0.28	0.27	0.28	0.29	0.29
Other chemical products	0.51	0.56	0.49	0.53	0.53	0.50	0.46	0.42	0.45	0.46	0.49	0.44	0.34	0.41	0.37	0.36	0.35	0.35	0.35	0.34
Industrial gases, inorganics and fertilisers (all inorganic chemicals) - 20.11/13/15	0.46	0.50	0.55	0.61	0.59	0.56	0.48	0.54	0.53	0.58	0.52	0.43	0.33	0.36	0.36	0.36	0.33	0.37	0.37	0.38
Petrochemicals - 20.14/16/17/60	0.59	0.64	0.61	0.58	0.52	0.47	0.46	0.48	0.48	0.48	0.49	0.42	0.31	0.35	0.33	0.35	0.32	0.34	0.33	0.33
Dyestuffs, agro-chemicals - 20.12/20	0.47	0.47	0.45	0.48	0.46	0.46	0.49	0.53	0.52	0.54	0.57	0.47	0.35	0.39	0.37	0.38	0.36	0.40	0.40	0.40
Basic pharmaceutical products and pharmaceutical preparations	0.64	0.64	0.65	0.68	0.64	0.60	0.54	0.57	0.55	0.52	0.51	0.42	0.31	0.35	0.32	0.33	0.32	0.33	0.33	0.33
Rubber and plastic products	0.62	0.61	0.59	0.60	0.57	0.54	0.50	0.51	0.51	0.51	0.51	0.43	0.33	0.36	0.35	0.36	0.34	0.35	0.36	0.36
Manufacture of cement, lime, plaster and articles of concrete, cement and plaster	5.76	5.81	5.42	3.63	3.40	3.31	3.07	3.05	2.86	2.60	2.40	2.00	1.95	1.94	1.91	1.96	1.84	1.94	1.91	1.89
Glass, refractory, clay, other porcelain and ceramic, stone and abrasive products - 23.1-4/7-9	2.10	2.15	2.08	1.83	1.73	1.67	1.61	1.63	1.55	1.43	1.33	1.16	1.09	1.14	1.07	1.10	1.03	1.07	1.06	1.05
Basic iron and steel	3.10	3.03	2.82	2.66	2.51	2.48	2.36	2.25	2.01	1.80	1.67	1.42	1.24	1.25	1.21	1.20	1.15	1.21	1.22	1.23
Other basic metals and casting	1.74	1.79	1.72	1.61	1.52	1.54	1.43	1.47	1.35	1.28	1.24	1.02	0.90	0.90	0.86	0.84	0.80	0.84	0.84	0.84
Weapons and ammunition	0.91	0.91	0.85	0.84	0.80	0.78	0.73	0.79	0.74	0.72	0.73	0.59	0.45	0.47	0.46	0.45	0.42	0.45	0.44	0.44
Fabricated metal products, excl. machinery and equipment and weapons & ammunition - 25.1-3/25.5-9	0.91	0.91	0.85	0.84	0.80	0.78	0.73	0.79	0.74	0.72	0.73	0.59	0.45	0.47	0.46	0.45	0.42	0.45	0.44	0.44
Computer, electronic and optical products	0.60	0.59	0.57	0.57	0.56	0.54	0.51	0.53	0.53	0.54	0.53	0.40	0.33	0.35	0.33	0.33	0.30	0.31	0.31	0.31
Electrical equipment	0.72	0.73	0.68	0.70	0.69	0.67	0.64	0.65	0.63	0.61	0.61	0.47	0.38	0.40	0.39	0.39	0.36	0.38	0.38	0.38
Machinery and equipment n.e.c.	0.67	0.68	0.64	0.64	0.60	0.59	0.55	0.58	0.55	0.54	0.54	0.47	0.37	0.39	0.38	0.38	0.35	0.37	0.37	0.37
Motor vehicles, trailers and semi-trailers	0.72	0.73	0.69	0.71	0.67	0.65	0.61	0.65	0.62	0.59	0.61	0.51	0.39	0.40	0.39	0.39	0.37	0.38	0.38	0.38
Ships and boats	0.72	0.72	0.68	0.72	0.71	0.67	0.65	0.70	0.65	0.63	0.61	0.54	0.44	0.42	0.40	0.40	0.38	0.39	0.39	0.39
Air and spacecraft and related machinery	0.72	0.72	0.68	0.72	0.71	0.67	0.65	0.70	0.65	0.63	0.61	0.54	0.44	0.42	0.40	0.40	0.38	0.39	0.39	0.39
Other transport equipment - 30.2/4/9	0.72	0.72	0.68	0.72	0.71	0.67	0.65	0.70	0.65	0.63	0.61	0.54	0.44	0.42	0.40	0.40	0.38	0.39	0.39	0.39
Furniture	0.74	0.74	0.71	0.70	0.66	0.63	0.60	0.62	0.61	0.57	0.57	0.44	0.36	0.38	0.39	0.39	0.36	0.37	0.37	0.37
Other manufactured goods	0.74	0.74	0.71	0.70	0.66	0.63	0.61	0.62	0.62	0.57	0.57	0.45	0.37	0.38	0.39	0.40	0.36	0.38	0.38	0.37
Repair and maintenance of ships and boats	0.72	0.72	0.68	0.72	0.71	0.67	0.65	0.70	0.65	0.63	0.61	0.54	0.44	0.42	0.40	0.40	0.38	0.39	0.39	0.39

Sector – EU	1997	1998	1999	2000	2001	2002	2003	2004	2005	2006	2007	2008	2009	2010	2011	2012	2013	2014	2015	2016
Repair and maintenance of aircraft and spacecraft	0.72	0.72	0.68	0.72	0.71	0.67	0.65	0.70	0.65	0.63	0.61	0.54	0.44	0.42	0.40	0.40	0.38	0.39	0.39	0.39
Rest of repair; Installation - 33.11-14/17/19/20	0.54	0.55	0.56	0.59	0.57	0.57	0.53	0.53	0.50	0.48	0.44	0.40	0.36	0.37	0.33	0.37	0.35	0.38	0.38	0.38
Electricity, transmission and distribution	6.63	7.15	7.06	7.88	7.31	7.23	6.25	6.04	5.47	5.05	4.89	3.45	2.89	2.82	2.76	2.83	2.66	2.82	2.82	2.81
Gas; distribution of gaseous fuels through mains; steam and air conditioning supply	13.16	11.22	11.23	11.13	9.65	9.51	8.91	8.76	7.68	7.07	7.04	4.92	4.39	4.47	4.18	4.26	4.12	4.24	4.33	4.41
Natural water; water treatment and supply services	1.70	1.67	1.50	1.74	1.66	1.62	1.51	1.43	1.30	1.23	1.17	0.69	0.53	0.49	0.49	0.48	0.42	0.43	0.42	0.40
Sewerage services; sewage sludge	0.84	0.86	0.86	0.77	0.87	0.78	0.70	0.68	0.54	0.50	0.49	0.40	0.36	0.35	0.34	0.35	0.33	0.34	0.34	0.34
Waste collection, treatment and disposal services; materials recovery services	0.84	0.86	0.86	0.77	0.87	0.78	0.70	0.68	0.54	0.50	0.49	0.40	0.36	0.35	0.34	0.35	0.33	0.34	0.34	0.34
Remediation services and other waste management services	0.84	0.86	0.86	0.77	0.87	0.78	0.70	0.68	0.54	0.50	0.49	0.40	0.36	0.35	0.34	0.35	0.33	0.34	0.34	0.34
Construction	0.99	1.01	0.96	0.84	0.80	0.77	0.71	0.71	0.69	0.65	0.63	0.53	0.43	0.45	0.44	0.45	0.42	0.43	0.43	0.42
Wholesale and retail trade and repair services of motor vehicles and motorcycles	0.40	0.41	0.42	0.45	0.42	0.41	0.39	0.39	0.38	0.37	0.36	0.30	0.26	0.26	0.25	0.26	0.24	0.26	0.26	0.26
Wholesale trade services, except of motor vehicles and motorcycles	0.00	0.00	0.00	0.00	0.00	0.00	0.00	0.00	0.00	0.00	0.00	0.00	0.00	0.00	0.00	0.00	0.00	0.00	0.00	0.00
Retail trade services, except of motor vehicles and motorcycles	0.00	0.00	0.00	0.00	0.00	0.00	0.00	0.00	0.00	0.00	0.00	0.00	0.00	0.00	0.00	0.00	0.00	0.00	0.00	0.00
Rail transport services	0.57	0.56	0.53	0.54	0.48	0.48	0.45	0.44	0.41	0.40	0.37	0.31	0.26	0.27	0.27	0.28	0.25	0.26	0.25	0.25
Land transport services and transport services via pipelines, excluding rail transport	0.46	0.45	0.44	0.47	0.45	0.45	0.42	0.42	0.42	0.41	0.39	0.33	0.27	0.28	0.29	0.29	0.27	0.27	0.27	0.27
Water transport services	8.31	8.44	8.55	7.71	6.40	6.74	6.06	6.12	5.61	5.65	5.20	4.46	4.93	4.66	4.53	4.45	4.14	4.34	4.21	4.08
Air transport services	2.30	2.30	2.28	2.35	2.14	2.14	1.92	1.98	1.92	1.79	1.71	1.53	1.46	1.45	1.43	1.48	1.39	1.43	1.40	1.37
Warehousing and support services for transportation	0.60	0.60	0.60	0.62	0.54	0.55	0.50	0.49	0.48	0.46	0.42	0.37	0.31	0.33	0.32	0.32	0.29	0.30	0.30	0.29
Postal and courier services	0.18	0.18	0.18	0.22	0.21	0.19	0.17	0.17	0.17	0.17	0.16	0.13	0.10	0.11	0.12	0.13	0.12	0.12	0.13	0.13
Accommodation services	0.63	0.62	0.61	0.64	0.60	0.57	0.50	0.52	0.47	0.46	0.44	0.34	0.25	0.26	0.26	0.26	0.24	0.25	0.25	0.25
Food and beverage serving services	0.63	0.62	0.61	0.64	0.60	0.57	0.50	0.52	0.47	0.46	0.44	0.34	0.25	0.26	0.26	0.26	0.24	0.25	0.25	0.25
Publishing services	0.30	0.30	0.29	0.31	0.31	0.29	0.27	0.27	0.27	0.27	0.26	0.23	0.19	0.19	0.18	0.19	0.17	0.18	0.18	0.18
Motion Picture, Video & TV Programme Production, Sound Recording & Music Publishing Activities & Programming And Broadcasting Activities	0.41	0.37	0.35	0.35	0.32	0.30	0.27	0.27	0.27	0.25	0.24	0.21	0.17	0.18	0.18	0.18	0.16	0.17	0.17	0.16
Telecommunications services	0.18	0.18	0.18	0.22	0.21	0.19	0.17	0.17	0.17	0.17	0.16	0.13	0.10	0.11	0.12	0.13	0.12	0.12	0.13	0.13
Computer programming, consultancy and related services	0.41	0.37	0.35	0.35	0.32	0.30	0.27	0.27	0.27	0.25	0.24	0.21	0.17	0.18	0.18	0.18	0.16	0.17	0.17	0.16
Information services	0.41	0.37	0.35	0.35	0.32	0.30	0.27	0.27	0.27	0.25	0.24	0.21	0.17	0.18	0.18	0.18	0.16	0.17	0.17	0.16
Financial services, except insurance and pension funding	0.25	0.25	0.24	0.26	0.26	0.24	0.22	0.21	0.21	0.23	0.20	0.18	0.14	0.13	0.13	0.13	0.12	0.13	0.13	0.12
Insurance and reinsurance, except compulsory social security & Pension funding	0.33	0.33	0.32	0.32	0.31	0.29	0.25	0.25	0.24	0.23	0.21	0.19	0.16	0.16	0.16	0.17	0.15	0.16	0.16	0.15
Services auxiliary to financial services and insurance services	0.29	0.28	0.27	0.29	0.27	0.26	0.23	0.21	0.21	0.20	0.18	0.16	0.13	0.14	0.14	0.14	0.13	0.13	0.13	0.13
Real estate services, excluding on a fee or contract basis and imputed rent	0.22	0.22	0.21	0.20	0.20	0.19	0.18	0.17	0.17	0.16	0.15	0.12	0.10	0.10	0.10	0.10	0.09	0.10	0.10	0.10
Owner-Occupiers' Housing Services	0.00	0.00	0.00	0.00	0.00	0.00	0.00	0.00	0.00	0.00	0.00	0.00	0.00	0.00	0.00	0.00	0.00	0.00	0.00	0.00
Real estate activities on a fee or contract basis	0.22	0.22	0.21	0.20	0.20	0.19	0.18	0.17	0.17	0.16	0.15	0.12	0.10	0.10	0.10	0.10	0.09	0.10	0.10	0.10
Legal services	0.30	0.30	0.29	0.31	0.31	0.29	0.27	0.27	0.27	0.27	0.26	0.23	0.19	0.19	0.18	0.19	0.17	0.18	0.18	0.18
Accounting, bookkeeping and auditing services; tax consulting services	0.30	0.30	0.29	0.31	0.31	0.29	0.27	0.27	0.27	0.27	0.26	0.23	0.19	0.19	0.18	0.19	0.17	0.18	0.18	0.18
Services of head offices; management consulting services	0.30	0.30	0.29	0.31	0.31	0.29	0.27	0.27	0.27	0.27	0.26	0.23	0.19	0.19	0.18	0.19	0.17	0.18	0.18	0.18
Architectural and engineering services; technical testing and analysis services	0.30	0.30	0.29	0.31	0.31	0.29	0.27	0.27	0.27	0.27	0.26	0.23	0.19	0.19	0.18	0.19	0.17	0.18	0.18	0.18
Scientific research and development services	0.50	0.49	0.47	0.48	0.47	0.43	0.41	0.41	0.40	0.39	0.37	0.27	0.22	0.23	0.22	0.23	0.21	0.21	0.21	0.21
Advertising and market research services	0.30	0.30	0.29	0.31	0.31	0.29	0.27	0.27	0.27	0.27	0.26	0.23	0.19	0.19	0.18	0.19	0.17	0.18	0.18	0.18

Sector – EU	1997	1998	1999	2000	2001	2002	2003	2004	2005	2006	2007	2008	2009	2010	2011	2012	2013	2014	2015	2016
Other professional, scientific and technical services	0.30	0.30	0.29	0.31	0.31	0.29	0.27	0.27	0.27	0.27	0.26	0.23	0.19	0.19	0.18	0.19	0.17	0.18	0.18	0.18
Veterinary services	0.30	0.30	0.29	0.31	0.31	0.29	0.27	0.27	0.27	0.27	0.26	0.23	0.19	0.19	0.18	0.19	0.17	0.18	0.18	0.18
Rental and leasing services	0.34	0.34	0.34	0.35	0.32	0.32	0.29	0.29	0.29	0.29	0.28	0.23	0.18	0.21	0.21	0.21	0.20	0.21	0.21	0.21
Employment services	0.30	0.30	0.29	0.31	0.31	0.29	0.27	0.27	0.27	0.27	0.26	0.23	0.19	0.19	0.18	0.19	0.17	0.18	0.18	0.18
Travel agency, tour operator and other reservation services and related services	0.30	0.30	0.29	0.31	0.31	0.29	0.27	0.27	0.27	0.27	0.26	0.23	0.19	0.19	0.18	0.19	0.17	0.18	0.18	0.18
Security and investigation services	0.30	0.30	0.29	0.31	0.31	0.29	0.27	0.27	0.27	0.27	0.26	0.23	0.19	0.19	0.18	0.19	0.17	0.18	0.18	0.18
Services to buildings and landscape	0.30	0.30	0.29	0.31	0.31	0.29	0.27	0.27	0.27	0.27	0.26	0.23	0.19	0.19	0.18	0.19	0.17	0.18	0.18	0.18
Office administrative, office support and other business support services	0.30	0.30	0.29	0.31	0.31	0.29	0.27	0.27	0.27	0.27	0.26	0.23	0.19	0.19	0.18	0.19	0.17	0.18	0.18	0.18
Public administration and defence services; compulsory social security services	0.36	0.36	0.35	0.34	0.35	0.34	0.31	0.30	0.29	0.29	0.25	0.20	0.17	0.17	0.17	0.17	0.16	0.16	0.16	0.16
Education services	0.30	0.30	0.29	0.30	0.28	0.26	0.26	0.25	0.23	0.24	0.21	0.15	0.14	0.13	0.12	0.13	0.12	0.12	0.12	0.11
Human health services	0.38	0.39	0.37	0.39	0.38	0.34	0.32	0.32	0.30	0.29	0.27	0.22	0.19	0.20	0.19	0.19	0.17	0.17	0.17	0.17
Residential Care & Social Work Activities	0.38	0.39	0.37	0.39	0.38	0.34	0.32	0.32	0.30	0.29	0.27	0.22	0.19	0.20	0.19	0.19	0.17	0.17	0.17	0.17
Creative, arts and entertainment services	0.45	0.44	0.42	0.47	0.44	0.43	0.40	0.40	0.39	0.38	0.36	0.30	0.24	0.24	0.24	0.25	0.23	0.24	0.24	0.24
Libraries, archives, museums and other cultural services	0.45	0.44	0.42	0.47	0.44	0.43	0.40	0.40	0.39	0.38	0.36	0.30	0.24	0.24	0.24	0.25	0.23	0.24	0.24	0.24
Gambling and betting services	0.45	0.44	0.42	0.47	0.44	0.43	0.40	0.40	0.39	0.38	0.36	0.30	0.24	0.24	0.24	0.25	0.23	0.24	0.24	0.24
Sports services and amusement and recreation services	0.45	0.44	0.42	0.47	0.44	0.43	0.40	0.40	0.39	0.38	0.36	0.30	0.24	0.24	0.24	0.25	0.23	0.24	0.24	0.24
Services furnished by membership organisations	0.51	0.53	0.51	0.52	0.49	0.46	0.46	0.45	0.45	0.44	0.41	0.37	0.38	0.51	0.28	0.29	0.27	0.28	0.28	0.27
Repair services of computers and personal and household goods	0.44	0.44	0.41	0.41	0.46	0.42	0.38	0.38	0.35	0.34	0.31	0.19	0.18	0.17	0.17	0.18	0.17	0.17	0.17	0.17
Other personal services	0.44	0.44	0.41	0.41	0.46	0.42	0.38	0.38	0.35	0.34	0.31	0.19	0.18	0.17	0.17	0.18	0.17	0.17	0.17	0.17
Services of households as employers of domestic personnel	0.00	0.00	0.00	0.00	0.00	0.00	0.00	0.00	0.00	0.00	0.00	0.00	0.00	0.00	0.00	0.00	0.00	0.00	0.00	0.00

Sector – China	1997	1998	1999	2000	2001	2002	2003	2004	2005	2006	2007	2008	2009	2010	2011	2012	2013	2014	2015	2016
Products of agriculture, hunting and related services	9.12	9.30	9.19	7.72	6.94	6.77	6.92	6.92	6.78	6.70	6.02	4.09	3.23	2.86	2.40	1.87	1.77	1.61	1.54	1.48
Products of forestry, logging and related services	0.76	0.48	0.31	0.39	0.43	0.52	2.27	0.86	1.04	1.07	0.95	0.76	0.93	1.14	1.16	1.10	1.06	1.03	1.08	1.12
Fish and other fishing products; aquaculture products; support services to fishing	0.08	0.08	0.07	0.12	0.19	0.31	0.49	0.40	0.40	0.42	0.26	0.25	0.25	0.24	0.25	0.21	0.18	0.16	0.15	0.14
Coal and lignite	34.64	35.29	31.92	29.48	26.95	28.63	30.03	35.10	26.34	24.28	23.29	12.41	12.84	14.95	11.72	9.70	9.27	9.57	9.44	9.31
Crude Petroleum And Natural Gas & Metal Ores	4.54	4.33	3.89	3.38	3.12	3.01	3.01	2.87	2.64	2.56	2.48	2.02	1.60	2.44	2.13	1.08	1.01	1.04	1.00	0.96
Other mining and quarrying products	3.16	3.23	2.45	2.63	2.41	2.30	3.30	2.37	2.47	2.85	3.03	2.53	2.33	3.21	2.97	2.11	1.91	1.92	1.85	1.78
Mining support services	4.92	4.76	4.37	3.78	3.58	3.53	3.64	3.37	2.97	2.80	2.74	2.18	1.92	2.67	2.28	1.24	1.19	1.26	1.23	1.20
Preserved meat and meat products	3.90	3.68	3.42	2.96	2.91	3.14	5.49	5.78	5.66	5.48	4.77	3.21	2.60	2.38	2.00	1.69	1.56	1.46	1.40	1.33
Processed and preserved fish, crustaceans, molluscs, fruit and vegetables	2.07	1.99	1.86	2.04	2.03	2.40	2.82	2.59	2.38	1.79	1.60	1.65	1.69	1.67	1.68	1.51	1.33	1.27	1.19	1.12
Vegetable and animal oils and fats	4.12	4.43	4.99	5.01	6.47	6.81	7.85	5.94	6.32	5.84	5.75	4.04	3.43	3.18	2.65	2.40	2.28	2.26	2.14	2.01
Dairy products	3.75	3.73	4.06	4.09	5.02	5.83	6.15	6.42	6.23	6.12	5.50	3.60	2.84	2.47	2.04	1.68	1.56	1.43	1.36	1.30
Grain mill products, starches and starch products	71.31	69.35	43.43	31.37	25.06	22.04	19.97	18.53	16.50	15.23	14.02	9.40	7.14	6.28	5.28	4.50	4.17	3.90	3.84	3.77
Bakery and farinaceous products	2.52	2.57	2.70	2.71	2.72	3.27	4.13	4.28	4.29	4.13	3.32	2.53	2.13	2.03	1.73	1.48	1.39	1.29	1.24	1.20
Other food products	2.52	2.57	2.70	2.71	2.72	3.27	4.12	4.27	4.29	4.13	3.32	2.53	2.13	2.03	1.73	1.48	1.39	1.29	1.24	1.20
Prepared animal feeds	2.52	2.57	2.70	2.71	2.72	3.27	4.13	4.28	4.29	4.13	3.32	2.53	2.13	2.03	1.73	1.48	1.39	1.29	1.24	1.20
Alcoholic beverages	5.73	5.73	4.73	4.08	3.69	3.80	4.19	4.24	4.27	4.17	3.55	2.64	2.15	1.97	1.76	1.58	1.45	1.36	1.31	1.25
Soft drinks	5.73	5.73	4.73	4.08	3.69	3.80	4.19	4.24	4.27	4.17	3.55	2.64	2.15	1.97	1.76	1.58	1.45	1.36	1.31	1.25
Tobacco products	10.28	9.52	6.34	4.59	4.63	5.03	5.68	5.86	5.66	5.47	4.67	3.33	2.97	3.11	2.52	2.21	2.03	1.91	1.82	1.72
Textiles	6.40	6.44	5.51	4.88	4.30	4.35	4.74	4.84	4.55	4.21	3.76	2.70	2.11	1.79	1.59	1.51	1.33	1.28	1.20	1.11
Wearing apparel	4.47	4.40	3.91	3.48	3.17	3.28	3.85	4.04	3.88	3.63	3.25	2.35	1.85	1.57	1.40	1.32	1.18	1.15	1.08	1.01

Sector – China	1997	1998	1999	2000	2001	2002	2003	2004	2005	2006	2007	2008	2009	2010	2011	2012	2013	2014	2015	2016
Leather and related products	3.84	3.83	3.42	3.01	2.75	2.93	3.39	3.71	3.55	3.26	2.88	2.17	1.73	1.54	1.40	1.41	1.27	1.25	1.19	1.13
Wood and of products of wood and cork, except furniture; articles of straw and plaiting materials	9.11	9.51	7.69	6.76	7.05	7.01	7.72	8.16	7.19	6.44	5.58	3.93	3.16	2.50	2.04	1.93	1.76	1.64	1.59	1.54
Paper and paper products	6.57	6.76	5.45	5.13	4.67	4.76	5.65	6.04	5.63	5.16	4.38	3.57	3.03	2.55	2.33	2.41	2.16	2.15	2.04	1.93
Printing and recording services	3.24	3.40	3.09	2.87	2.62	2.71	3.37	3.58	3.49	3.31	2.92	2.32	1.94	1.64	1.54	1.56	1.38	1.36	1.29	1.22
Coke and refined petroleum products	8.27	8.80	8.22	6.94	6.56	6.61	7.21	7.71	6.49	5.72	5.38	3.58	3.67	3.17	2.69	2.42	2.25	2.29	2.22	2.15
Paints, varnishes and similar coatings, printing ink and mastics	7.10	6.55	6.09	5.37	4.76	4.86	5.46	5.46	5.49	5.13	4.74	3.47	3.05	2.79	2.49	2.40	2.04	1.94	1.81	1.68
Soap and detergents, cleaning and polishing preparations, perfumes and toilet preparations	5.70	5.78	5.11	4.47	4.01	3.97	4.37	4.19	4.08	3.90	3.55	2.58	2.26	2.09	1.93	1.81	1.63	1.62	1.55	1.47
Other chemical products	6.24	6.67	5.28	4.83	4.64	4.78	5.13	4.55	4.73	4.40	4.34	3.48	3.11	3.04	2.59	2.35	2.15	2.01	1.87	1.73
Industrial gases, inorganics and fertilisers (all inorganic chemicals) - 20.11/13/15	5.61	5.95	5.96	5.56	5.14	5.33	5.37	5.84	5.54	5.52	4.62	3.41	2.99	2.70	2.54	2.34	2.04	2.12	2.01	1.90
Petrochemicals - 20.14/16/17/60	7.13	7.73	6.63	5.27	4.51	4.52	5.14	5.19	5.01	4.59	4.41	3.28	2.83	2.59	2.35	2.25	1.99	1.92	1.79	1.65
Dyestuffs, agro-chemicals - 20.12/20	5.78	5.69	4.83	4.38	3.98	4.39	5.47	5.71	5.41	5.22	5.06	3.73	3.19	2.90	2.60	2.48	2.21	2.27	2.15	2.02
Basic pharmaceutical products and pharmaceutical preparations	7.75	7.62	7.03	6.16	5.61	5.76	6.07	6.14	5.74	4.99	4.56	3.32	2.83	2.59	2.28	2.15	1.94	1.87	1.78	1.68
Rubber and plastic products	7.57	7.69	7.11	6.27	5.79	6.32	7.35	8.46	8.91	8.00	5.96	4.57	3.64	3.47	3.22	3.13	2.77	2.72	2.53	2.34
Manufacture of cement, lime, plaster and articles of concrete, cement and plaster	178.77	175.40	150.46	128.43	113.51	106.99	92.28	94.06	78.50	66.53	57.96	42.28	36.56	29.13	24.99	24.19	22.73	23.33	22.77	22.21
Glass, refractory, clay, other porcelain and ceramic, stone and abrasive products - 23.1-4/7-9	22.71	18.46	14.34	13.17	11.53	11.30	10.62	11.71	10.59	9.43	8.51	6.67	5.64	4.52	4.11	4.06	3.74	3.80	3.67	3.54
Basic iron and steel	12.11	11.62	9.82	8.72	7.82	7.51	7.36	7.81	7.80	7.47	7.69	5.81	5.17	4.69	4.39	4.35	4.00	4.09	3.94	3.79
Other basic metals and casting	8.16	7.87	6.73	5.74	5.33	5.39	5.35	5.49	5.14	4.80	4.60	3.47	2.97	3.02	2.73	2.17	1.96	1.96	1.85	1.75
Weapons and ammunition	7.85	7.70	6.73	5.96	5.46	5.51	5.65	6.06	5.95	5.75	5.64	4.37	3.79	3.40	3.24	3.20	2.88	2.88	2.71	2.54
Fabricated metal products, excl. machinery and equipment and weapons & ammunition - 25.1-3/25.5-9	7.85	7.70	6.73	5.96	5.46	5.51	5.65	6.06	5.95	5.75	5.64	4.37	3.79	3.40	3.24	3.20	2.88	2.88	2.71	2.54
Computer, electronic and optical products	4.65	4.29	3.64	3.18	2.90	2.95	3.01	3.34	3.38	3.18	2.89	2.22	1.86	1.67	1.57	1.53	1.36	1.32	1.21	1.11
Electrical equipment	5.68	5.57	4.86	4.31	3.92	4.00	4.02	4.32	4.33	4.13	3.92	3.02	2.62	2.25	2.14	2.08	1.89	1.90	1.82	1.73
Machinery and equipment n.e.c.	5.85	5.70	4.92	4.27	3.90	3.94	4.11	4.37	4.34	4.17	4.05	3.13	2.73	2.22	2.12	2.13	1.93	1.94	1.86	1.77
Motor vehicles, trailers and semi-trailers	4.18	4.42	3.99	3.49	3.21	3.16	3.02	3.41	3.55	3.41	3.20	2.62	2.32	1.78	1.72	1.77	1.60	1.61	1.55	1.48
Ships and boats	5.25	5.25	4.68	4.19	3.71	3.64	3.58	3.79	3.73	3.46	3.34	2.75	2.39	1.87	1.82	1.80	1.61	1.61	1.54	1.47
Air and spacecraft and related machinery	5.25	5.25	4.68	4.19	3.71	3.64	3.58	3.79	3.73	3.46	3.34	2.75	2.39	1.87	1.82	1.80	1.61	1.61	1.54	1.47
Other transport equipment - 30.2/4/9	5.25	5.25	4.68	4.19	3.71	3.64	3.58	3.79	3.73	3.46	3.34	2.75	2.39	1.87	1.82	1.80	1.61	1.61	1.54	1.47
Furniture	5.69	5.65	5.02	4.43	4.03	3.97	4.79	5.14	4.83	4.26	3.54	2.75	2.24	1.92	1.72	1.67	1.46	1.42	1.31	1.20
Other manufactured goods	5.52	5.47	4.86	4.32	3.93	3.85	4.71	5.06	4.75	4.19	3.49	2.72	2.22	1.92	1.72	1.67	1.46	1.42	1.31	1.19
Repair and maintenance of ships and boats	5.25	5.25	4.68	4.19	3.71	3.64	3.58	3.79	3.73	3.46	3.34	2.75	2.39	1.87	1.82	1.80	1.61	1.61	1.54	1.47
Repair and maintenance of aircraft and spacecraft	5.25	5.25	4.68	4.19	3.71	3.64	3.58	3.79	3.73	3.46	3.34	2.75	2.39	1.87	1.82	1.80	1.61	1.61	1.54	1.47
Rest of repair; Installation - 33.11-14/17/19/20	1.47	1.09	0.86	0.54	0.46	0.38	0.32	0.41	0.27	0.15	0.09	0.07	0.05	0.04	0.05	0.05	0.04	0.04	0.04	0.04
Electricity, transmission and distribution	50.88	53.48	50.01	46.57	44.69	49.52	54.71	62.42	50.49	43.53	40.89	29.97	29.45	30.59	25.20	21.17	18.78	18.06	16.48	14.90
Gas; distribution of gaseous fuels through mains; steam and air conditioning supply	146.24	157.49	150.45	153.17	146.97	138.15	150.50	155.75	122.32	96.23	75.94	53.84	52.61	46.71	35.93	33.79	30.69	28.98	27.53	26.09
Natural water; water treatment and supply services	2.10	2.70	2.91	3.33	4.95	5.70	7.24	7.93	8.36	8.86	7.33	6.24	6.39	9.93	8.40	5.04	4.62	4.63	4.37	4.10
Sewerage services; sewage sludge	0.00	0.00	0.00	0.00	0.00	0.00	1.85	1.99	1.11	0.71	0.51	1.98	1.92	2.31	2.15	2.07	1.89	1.82	1.79	1.75
Waste collection, treatment and disposal services; materials recovery services	0.00	0.00	0.00	0.00	0.00	0.00	1.85	1.99	1.11	0.71	0.51	1.98	1.92	2.31	2.15	2.07	1.89	1.82	1.79	1.75
Remediation services and other waste management services	0.00	0.00	0.00	0.00	0.00	0.00	1.85	1.99	1.11	0.71	0.51	1.98	1.92	2.31	2.15	2.07	1.89	1.82	1.79	1.75
Construction	7.75	8.45	8.03	7.68	7.06	7.12	7.51	8.24	7.72	7.17	6.71	5.01	4.07	3.36	3.03	3.03	2.77	2.77	2.69	2.60
Wholesale and retail trade and repair services of motor vehicles and motorcycles	0.28	0.13	0.11	0.13	0.12	0.11	0.23	0.29	0.24	0.16	0.13	0.07	0.04	0.03	0.04	0.03	0.03	0.03	0.03	0.02

Sector – China	1997	1998	1999	2000	2001	2002	2003	2004	2005	2006	2007	2008	2009	2010	2011	2012	2013	2014	2015	2016
Wholesale trade services, except of motor vehicles and motorcycles	0.00	0.00	0.00	0.00	0.00	0.00	0.00	0.00	0.00	0.00	0.00	0.00	0.00	0.00	0.00	0.00	0.00	0.00	0.00	0.00
Retail trade services, except of motor vehicles and motorcycles	0.00	0.00	0.00	0.00	0.00	0.00	0.00	0.00	0.00	0.00	0.00	0.00	0.00	0.00	0.00	0.00	0.00	0.00	0.00	0.00
Rail transport services	7.29	7.30	6.26	5.74	5.08	5.13	5.73	6.08	5.75	5.12	4.67	3.51	2.88	2.34	2.09	2.08	1.79	1.79	1.72	1.65
Land transport services and transport services via pipelines, excluding rail transport	2.32	2.33	2.34	2.53	2.38	2.52	2.96	3.45	3.41	3.35	3.30	2.44	2.19	1.80	1.68	1.67	1.54	1.56	1.52	1.48
Water transport services	15.83	13.96	15.00	13.50	11.55	11.42	11.59	11.34	10.23	9.44	8.53	5.94	5.23	4.57	4.07	3.84	3.44	3.31	3.13	2.95
Air transport services	6.40	5.86	5.79	5.03	4.57	4.83	5.00	5.62	5.34	5.00	4.80	3.79	3.06	2.60	2.39	2.49	2.20	2.13	2.01	1.88
Warehousing and support services for transportation	1.15	0.96	0.96	0.98	0.88	0.88	1.10	1.25	1.24	1.23	1.18	0.91	0.73	0.68	0.55	0.63	0.56	0.53	0.50	0.47
Postal and courier services	5.47	5.00	4.35	3.78	3.17	3.00	4.01	3.96	3.47	3.07	2.61	2.22	1.92	1.12	1.02	1.07	0.90	0.82	0.78	0.74
Accommodation services	2.61	2.46	2.40	2.01	1.99	2.07	2.15	2.46	2.34	2.10	1.82	1.33	1.08	0.91	0.77	0.67	0.61	0.55	0.51	0.48
Food and beverage serving services	2.61	2.46	2.40	2.01	1.99	2.07	2.15	2.46	2.34	2.10	1.82	1.33	1.08	0.91	0.77	0.67	0.61	0.55	0.51	0.48
Publishing services	2.92	2.76	2.42	2.11	1.84	1.86	2.03	2.20	2.18	2.07	1.81	1.40	1.16	0.94	0.88	0.82	0.70	0.66	0.62	0.59
Motion Picture, Video & TV Programme Production, Sound Recording & Music Publishing Activities & Programming And Broadcasting Activities	3.41	3.22	2.76	2.39	1.99	1.93	2.06	2.00	1.79	1.47	0.99	0.89	0.73	0.67	0.64	0.54	0.38	0.30	0.30	0.30
Telecommunications services	5.47	5.00	4.35	3.78	3.17	3.00	4.01	3.96	3.47	3.07	2.61	2.22	1.92	1.12	1.02	1.07	0.90	0.82	0.78	0.74
Computer programming, consultancy and related services	3.41	3.22	2.76	2.39	1.99	1.93	2.06	2.00	1.79	1.47	0.99	0.89	0.73	0.67	0.64	0.54	0.38	0.30	0.30	0.30
Information services	3.41	3.22	2.76	2.39	1.99	1.93	2.06	2.00	1.79	1.47	0.99	0.89	0.73	0.67	0.64	0.54	0.38	0.30	0.30	0.30
Financial services, except insurance and pension funding	1.56	1.45	1.32	1.16	0.94	0.88	0.74	0.73	0.70	0.63	0.48	0.43	0.33	0.34	0.34	0.30	0.23	0.21	0.20	0.19
Insurance and reinsurance, except compulsory social security & Pension funding	1.83	1.70	1.62	1.50	1.21	1.17	0.98	0.98	0.97	0.87	0.64	0.56	0.41	0.38	0.37	0.34	0.28	0.26	0.25	0.25
Services auxiliary to financial services and insurance services	1.77	1.63	1.50	1.33	1.12	1.07	1.04	1.11	1.07	0.96	0.80	0.66	0.54	0.48	0.45	0.41	0.35	0.33	0.32	0.31
Real estate services, excluding on a fee or contract basis and imputed rent	2.53	2.20	1.86	1.62	1.21	1.08	1.01	0.89	0.74	0.53	0.30	0.37	0.36	0.31	0.32	0.29	0.22	0.20	0.20	0.21
Owner-Occupiers' Housing Services	0.00	0.00	0.00	0.00	0.00	0.00	0.00	0.00	0.00	0.00	0.00	0.00	0.00	0.00	0.00	0.00	0.00	0.00	0.00	0.00
Real estate activities on a fee or contract basis	2.53	2.20	1.86	1.62	1.21	1.08	1.01	0.89	0.74	0.53	0.30	0.37	0.36	0.31	0.32	0.29	0.22	0.20	0.20	0.21
Legal services	2.92	2.76	2.42	2.11	1.84	1.86	2.03	2.20	2.18	2.07	1.81	1.40	1.16	0.94	0.88	0.82	0.70	0.66	0.62	0.59
Accounting, bookkeeping and auditing services; tax consulting services	2.92	2.76	2.42	2.11	1.84	1.86	2.03	2.20	2.18	2.07	1.81	1.40	1.16	0.94	0.88	0.82	0.70	0.66	0.62	0.59
Services of head offices; management consulting services	2.92	2.76	2.42	2.11	1.84	1.86	2.03	2.20	2.18	2.07	1.81	1.40	1.16	0.94	0.88	0.82	0.70	0.66	0.62	0.59
Architectural and engineering services; technical testing and analysis services	2.92	2.76	2.42	2.11	1.84	1.86	2.03	2.20	2.18	2.07	1.81	1.40	1.16	0.94	0.88	0.82	0.70	0.66	0.62	0.59
Scientific research and development services	5.92	5.73	5.10	4.57	4.02	4.03	1.93	1.80	1.84	1.66	1.18	1.68	1.63	2.15	1.96	1.74	1.54	1.50	1.42	1.33
Advertising and market research services	2.92	2.76	2.42	2.11	1.84	1.86	2.03	2.20	2.18	2.07	1.81	1.40	1.16	0.94	0.88	0.82	0.70	0.66	0.62	0.59
Other professional, scientific and technical services	2.92	2.76	2.42	2.11	1.84	1.86	2.03	2.20	2.18	2.07	1.81	1.40	1.16	0.94	0.88	0.82	0.70	0.66	0.62	0.59
Veterinary services	2.92	2.76	2.42	2.11	1.84	1.86	2.03	2.20	2.18	2.07	1.81	1.40	1.16	0.94	0.88	0.82	0.70	0.66	0.62	0.59
Rental and leasing services	2.72	2.54	2.27	2.03	1.76	1.89	2.14	2.46	2.34	2.20	2.00	1.75	1.44	1.21	1.18	1.12	1.01	0.99	0.96	0.92
Employment services	2.92	2.76	2.42	2.11	1.84	1.86	2.03	2.20	2.18	2.07	1.81	1.40	1.16	0.94	0.88	0.82	0.70	0.66	0.62	0.59
Travel agency, tour operator and other reservation services and related services	2.92	2.76	2.42	2.11	1.84	1.86	2.03	2.20	2.18	2.07	1.81	1.40	1.16	0.94	0.88	0.82	0.70	0.66	0.62	0.59
Security and investigation services	2.92	2.76	2.42	2.11	1.84	1.86	2.03	2.20	2.18	2.07	1.81	1.40	1.16	0.94	0.88	0.82	0.70	0.66	0.62	0.59
Services to buildings and landscape	2.92	2.76	2.42	2.11	1.84	1.86	2.03	2.20	2.18	2.07	1.81	1.40	1.16	0.94	0.88	0.82	0.70	0.66	0.62	0.59
Office administrative, office support and other business support services	2.92	2.76	2.42	2.11	1.84	1.86	2.03	2.20	2.18	2.07	1.81	1.40	1.16	0.94	0.88	0.82	0.70	0.66	0.62	0.59
Public administration and defence services; compulsory social security services	3.45	3.41	3.16	2.85	2.44	2.40	2.79	2.90	2.65	2.37	2.02	1.45	1.15	1.06	0.96	0.85	0.70	0.63	0.59	0.56
Education services	6.12	5.96	5.35	4.84	4.03	3.86	4.03	3.95	3.45	2.91	2.26	1.52	1.05	1.03	0.86	0.71	0.56	0.48	0.42	0.37
Human health services	3.19	3.38	3.25	3.03	2.73	2.83	2.97	3.14	3.20	3.14	2.99	2.21	1.83	1.65	1.57	1.50	1.36	1.36	1.32	1.29

Sector – China	1997	1998	1999	2000	2001	2002	2003	2004	2005	2006	2007	2008	2009	2010	2011	2012	2013	2014	2015	2016
Residential Care & Social Work Activities	3.19	3.38	3.25	3.03	2.73	2.83	2.97	3.14	3.20	3.14	2.99	2.21	1.83	1.65	1.57	1.50	1.36	1.36	1.32	1.29
Creative, arts and entertainment services	3.49	3.35	3.07	2.73	2.32	2.31	2.55	2.56	2.45	2.23	1.86	1.35	1.01	0.95	0.88	0.79	0.65	0.58	0.55	0.51
Libraries, archives, museums and other cultural services	3.49	3.35	3.07	2.73	2.32	2.31	2.55	2.56	2.45	2.23	1.86	1.35	1.01	0.95	0.88	0.79	0.65	0.58	0.55	0.51
Gambling and betting services	3.49	3.35	3.07	2.73	2.32	2.31	2.55	2.56	2.45	2.23	1.86	1.35	1.01	0.95	0.88	0.79	0.65	0.58	0.55	0.51
Sports services and amusement and recreation services	3.49	3.35	3.07	2.73	2.32	2.31	2.55	2.56	2.45	2.23	1.86	1.35	1.01	0.95	0.88	0.79	0.65	0.58	0.55	0.51
Services furnished by membership organisations	2.94	2.87	2.64	2.45	2.14	2.15	2.40	2.67	2.56	2.46	2.32	1.67	1.38	1.17	1.08	1.00	0.88	0.85	0.83	0.81
Repair services of computers and personal and household goods	5.06	4.80	4.28	3.59	2.92	2.66	2.80	2.65	2.32	1.93	1.45	1.09	0.82	0.81	0.72	0.60	0.48	0.43	0.41	0.40
Other personal services	5.06	4.80	4.28	3.59	2.92	2.66	2.80	2.65	2.32	1.93	1.45	1.09	0.82	0.81	0.72	0.60	0.48	0.43	0.41	0.40
Services of households as employers of domestic personnel	0.00	0.00	0.00	0.00	0.00	0.00	0.00	0.00	0.00	0.00	0.00	0.00	0.00	0.00	0.00	0.00	0.00	0.00	0.00	0.00

Sector – Rest-of-World	1997	1998	1999	2000	2001	2002	2003	2004	2005	2006	2007	2008	2009	2010	2011	2012	2013	2014	2015	2016
Products of agriculture, hunting and related services	6.96	7.48	7.53	6.94	6.82	6.98	6.77	6.76	6.36	6.01	5.67	4.53	4.04	3.41	3.09	2.78	2.63	2.69	2.60	2.50
Products of forestry, logging and related services	0.76	0.75	0.74	0.79	0.90	0.88	0.79	0.85	0.72	0.55	0.51	0.50	0.45	0.45	0.45	0.46	0.39	0.43	0.41	0.39
Fish and other fishing products; aquaculture products; support services to fishing	0.93	0.88	0.77	0.73	0.77	0.84	0.92	0.95	0.87	0.79	0.77	0.72	0.62	0.63	0.63	0.64	0.63	0.67	0.65	0.62
Coal and lignite	42.12	51.86	55.93	45.87	49.71	81.54	72.52	65.93	52.21	47.64	44.56	28.51	28.61	24.48	20.80	19.56	20.07	21.75	21.43	21.10
Crude Petroleum And Natural Gas & Metal Ores	5.76	7.10	6.25	4.12	4.30	4.38	4.32	3.80	3.05	2.56	2.60	1.88	2.14	1.68	1.42	1.39	1.32	1.39	1.35	1.32
Other mining and quarrying products	1.18	1.29	1.25	1.18	1.22	1.12	1.15	1.19	1.19	0.98	1.17	0.88	0.88	0.76	0.85	0.96	0.88	1.01	1.01	1.02
Mining support services	6.31	7.93	6.74	4.36	4.61	4.69	4.64	4.04	3.18	2.66	2.69	1.94	2.28	1.75	1.46	1.43	1.36	1.44	1.41	1.37
Preserved meat and meat products	3.89	4.24	4.16	3.84	3.97	4.05	3.87	3.94	3.65	3.39	3.13	2.49	2.29	1.87	1.79	1.61	1.55	1.58	1.52	1.47
Processed and preserved fish, crustaceans, molluscs, fruit and vegetables	1.74	1.87	1.69	1.48	1.50	1.60	1.63	1.68	1.55	1.43	1.35	1.21	1.17	1.06	0.99	0.96	0.93	0.97	0.94	0.91
Vegetable and animal oils and fats	3.73	4.28	4.15	3.59	3.72	3.89	3.87	3.76	3.33	3.07	3.07	2.52	2.36	2.00	1.80	1.65	1.56	1.59	1.55	1.50
Dairy products	3.88	4.20	4.13	3.88	3.86	3.90	3.88	4.03	3.66	3.52	3.41	2.87	2.45	2.10	1.99	1.80	1.75	1.76	1.70	1.65
Grain mill products, starches and starch products	4.87	5.04	5.32	4.93	4.73	4.99	4.80	4.84	4.46	4.13	3.91	3.20	2.95	2.54	2.35	2.23	2.18	2.26	2.22	2.18
Bakery and farinaceous products	2.54	2.65	2.53	2.47	2.49	2.59	2.68	2.81	2.51	2.47	2.43	2.13	1.81	1.57	1.51	1.37	1.35	1.34	1.30	1.26
Other food products	2.55	2.66	2.53	2.48	2.50	2.60	2.69	2.82	2.52	2.47	2.44	2.14	1.82	1.58	1.51	1.38	1.35	1.34	1.31	1.27
Prepared animal feeds	2.54	2.65	2.53	2.47	2.49	2.59	2.68	2.81	2.51	2.47	2.43	2.13	1.81	1.57	1.51	1.37	1.35	1.34	1.30	1.26
Alcoholic beverages	1.85	2.04	1.83	1.65	1.64	1.77	1.85	1.90	1.76	1.75	1.78	1.59	1.37	1.20	1.13	1.06	1.07	1.08	1.06	1.03
Soft drinks	1.85	2.04	1.83	1.65	1.64	1.77	1.85	1.90	1.76	1.75	1.78	1.59	1.37	1.20	1.13	1.06	1.07	1.08	1.06	1.03
Tobacco products	0.84	0.95	0.87	0.77	0.72	0.83	0.85	0.94	0.87	0.86	0.91	0.82	0.70	0.62	0.62	0.58	0.59	0.60	0.58	0.57
Textiles	1.87	1.97	1.84	1.69	1.72	1.75	1.83	1.78	1.63	1.52	1.49	1.25	1.09	0.98	0.93	0.89	0.84	0.86	0.84	0.81
Wearing apparel	1.24	1.31	1.23	1.17	1.15	1.18	1.23	1.27	1.16	1.09	1.03	0.86	0.75	0.68	0.64	0.62	0.59	0.59	0.57	0.54
Leather and related products	1.27	1.39	1.33	1.16	1.18	1.29	1.33	1.40	1.27	1.20	1.17	0.99	0.91	0.80	0.75	0.74	0.70	0.72	0.70	0.68
Wood and of products of wood and cork, except furniture; articles of straw and plaiting materials	1.11	1.15	1.11	1.10	1.08	1.10	1.09	1.12	0.97	1.00	0.99	0.93	0.87	0.80	0.79	0.78	0.79	0.80	0.80	0.80
Paper and paper products	1.60	1.69	1.59	1.63	1.73	1.82	1.89	1.94	1.71	1.69	1.66	1.56	1.29	1.19	1.14	1.10	1.11	1.08	1.06	1.04
Printing and recording services	0.64	0.69	0.64	0.62	0.64	0.65	0.63	0.63	0.52	0.57	0.54	0.49	0.42	0.40	0.38	0.35	0.36	0.33	0.32	0.31
Coke and refined petroleum products	5.00	6.46	5.82	4.30	4.27	4.34	4.12	3.97	3.30	2.93	2.90	2.22	2.14	1.79	1.66	1.51	1.43	1.49	1.45	1.41
Paints, varnishes and similar coatings, printing ink and mastics	1.72	1.67	1.58	1.54	1.46	1.47	1.52	1.56	1.50	1.52	1.52	1.36	1.16	1.04	1.02	0.97	0.91	0.85	0.82	0.79
Soap and detergents, cleaning and polishing preparations, perfumes and toilet preparations	1.38	1.48	1.32	1.28	1.23	1.20	1.22	1.20	1.11	1.15	1.14	1.01	0.86	0.78	0.79	0.73	0.73	0.71	0.70	0.69
Other chemical products	1.51	1.70	1.37	1.38	1.43	1.44	1.43	1.30	1.29	1.30	1.39	1.36	1.18	1.14	1.06	0.95	0.96	0.88	0.85	0.81
Industrial gases, inorganics and fertilisers (all inorganic chemicals) - 20.11/13/15	1.36	1.52	1.55	1.59	1.58	1.61	1.50	1.66	1.51	1.63	1.48	1.33	1.13	1.01	1.04	0.95	0.91	0.93	0.91	0.89
Petrochemicals - 20.14/16/17/60	1.73	1.98	1.72	1.51	1.39	1.37	1.44	1.48	1.37	1.36	1.42	1.28	1.07	0.97	0.96	0.91	0.89	0.85	0.81	0.77
Dyestuffs, agro-chemicals - 20.12/20	1.40	1.45	1.25	1.26	1.22	1.33	1.53	1.63	1.48	1.55	1.62	1.45	1.21	1.09	1.06	1.00	0.99	1.00	0.97	0.95

**Sector – Rest-of-World**	**1997**	**1998**	**1999**	**2000**	**2001**	**2002**	**2003**	**2004**	**2005**	**2006**	**2007**	**2008**	**2009**	**2010**	**2011**	**2012**	**2013**	**2014**	**2015**	**2016**
Basic pharmaceutical products and pharmaceutical preparations	1.88	1.95	1.82	1.76	1.72	1.74	1.70	1.75	1.57	1.48	1.46	1.30	1.08	0.97	0.93	0.87	0.87	0.82	0.80	0.78
Rubber and plastic products	2.40	2.58	2.38	2.03	2.09	2.19	2.26	2.30	2.14	2.22	2.27	2.07	1.83	1.64	1.52	1.55	1.52	1.53	1.52	1.52
Manufacture of cement, lime, plaster and articles of concrete, cement and plaster	9.08	9.36	8.86	9.18	9.07	9.20	9.83	10.16	9.33	9.38	9.67	8.36	8.67	7.94	8.54	8.73	8.57	9.10	9.03	8.97
Glass, refractory, clay, other porcelain and ceramic, stone and abrasive products - 23.1-4/7-9	2.97	3.32	3.10	2.69	2.74	2.91	2.94	3.08	2.71	2.69	2.71	2.32	2.22	1.98	1.89	1.91	1.87	1.95	1.93	1.91
Basic iron and steel	4.41	5.06	4.70	3.90	4.07	4.46	4.36	4.32	3.73	3.36	3.51	2.90	2.90	2.55	2.39	2.40	2.35	2.45	2.43	2.41
Other basic metals and casting	3.59	4.19	3.99	3.13	3.26	3.37	3.36	3.11	2.60	2.30	2.32	1.89	1.97	1.66	1.47	1.41	1.38	1.45	1.43	1.42
Weapons and ammunition	1.73	1.86	1.70	1.50	1.51	1.64	1.63	1.72	1.50	1.47	1.53	1.31	1.18	1.08	1.02	0.99	0.98	1.00	0.98	0.96
Fabricated metal products, excl. machinery and equipment and weapons & ammunition - 25.1-3/25.5-9	1.73	1.86	1.70	1.50	1.51	1.64	1.63	1.72	1.50	1.47	1.53	1.31	1.18	1.08	1.02	0.99	0.98	1.00	0.98	0.96
Computer, electronic and optical products	1.14	1.22	1.14	1.05	1.10	1.13	1.16	1.21	1.13	1.12	1.12	1.01	0.91	0.85	0.83	0.84	0.82	0.82	0.80	0.78
Electrical equipment	1.35	1.54	1.44	1.27	1.28	1.32	1.34	1.43	1.30	1.25	1.30	1.09	0.99	0.89	0.86	0.86	0.83	0.84	0.82	0.80
Machinery and equipment n.e.c.	1.33	1.39	1.29	1.18	1.18	1.25	1.26	1.33	1.21	1.17	1.20	1.04	0.92	0.85	0.82	0.82	0.80	0.82	0.80	0.79
Motor vehicles, trailers and semi-trailers	1.33	1.38	1.29	1.19	1.22	1.25	1.26	1.34	1.24	1.18	1.21	1.06	0.96	0.88	0.84	0.82	0.80	0.81	0.79	0.77
Ships and boats	1.02	1.08	0.99	0.94	0.94	0.97	0.99	1.06	0.92	0.92	0.94	0.92	0.74	0.68	0.67	0.65	0.64	0.64	0.62	0.61
Air and spacecraft and related machinery	1.02	1.08	0.99	0.94	0.94	0.97	0.99	1.06	0.92	0.92	0.94	0.92	0.74	0.68	0.67	0.65	0.64	0.64	0.62	0.61
Other transport equipment - 30.2/4/9	1.02	1.08	0.99	0.94	0.94	0.97	0.99	1.06	0.92	0.92	0.94	0.92	0.74	0.68	0.67	0.65	0.64	0.64	0.62	0.61
Furniture	2.17	2.40	2.24	1.95	2.03	2.07	2.12	2.22	2.04	2.02	2.09	1.88	1.67	1.60	1.45	1.48	1.49	1.56	1.56	1.56
Other manufactured goods	2.18	2.41	2.24	1.96	2.03	2.08	2.13	2.22	2.04	2.01	2.08	1.87	1.67	1.59	1.44	1.47	1.48	1.55	1.54	1.54
Repair and maintenance of ships and boats	1.02	1.08	0.99	0.94	0.94	0.97	0.99	1.06	0.92	0.92	0.94	0.92	0.74	0.68	0.67	0.65	0.64	0.64	0.62	0.61
Repair and maintenance of aircraft and spacecraft	1.02	1.08	0.99	0.94	0.94	0.97	0.99	1.06	0.92	0.92	0.94	0.92	0.74	0.68	0.67	0.65	0.64	0.64	0.62	0.61
Rest of repair; Installation - 33.11-14/17/19/20	0.83	0.95	0.89	0.80	0.79	0.89	0.86	0.87	0.79	0.73	0.70	0.56	0.53	0.47	0.45	0.45	0.43	0.45	0.44	0.43
Electricity, transmission and distribution	22.90	25.36	23.17	17.28	14.85	15.85	16.49	17.11	17.42	14.07	17.00	11.73	10.71	9.67	9.65	11.49	9.93	12.26	12.31	12.36
Gas; distribution of gaseous fuels through mains; steam and air conditioning supply	28.07	32.02	28.42	21.10	17.87	20.32	21.73	22.01	21.91	18.42	18.30	13.74	11.85	10.44	10.79	12.47	9.54	11.71	11.42	11.14
Natural water; water treatment and supply services	1.95	2.18	1.75	0.78	0.78	1.03	0.86	0.99	1.52	1.26	1.88	1.37	1.08	1.28	1.11	1.48	1.12	1.59	1.58	1.56
Sewerage services; sewage sludge	1.77	1.76	1.44	1.30	1.29	1.32	1.33	1.25	1.18	1.07	1.08	0.91	0.85	0.69	0.70	0.71	0.65	0.69	0.66	0.64
Waste collection, treatment and disposal services; materials recovery services	1.77	1.76	1.44	1.30	1.29	1.32	1.33	1.25	1.18	1.07	1.08	0.91	0.85	0.69	0.70	0.71	0.65	0.69	0.66	0.64
Remediation services and other waste management services	1.77	1.76	1.44	1.30	1.29	1.32	1.33	1.25	1.18	1.07	1.08	0.91	0.85	0.69	0.70	0.71	0.65	0.69	0.66	0.64
Construction	1.47	1.53	1.42	1.32	1.29	1.37	1.41	1.47	1.37	1.36	1.37	1.20	1.08	1.03	0.98	0.96	0.95	0.97	0.95	0.93
Wholesale and retail trade and repair services of motor vehicles and motorcycles	0.36	0.39	0.35	0.34	0.34	0.36	0.36	0.39	0.36	0.36	0.36	0.34	0.30	0.28	0.26	0.26	0.25	0.25	0.24	0.23
Wholesale trade services, except of motor vehicles and motorcycles	0.00	0.00	0.00	0.00	0.00	0.00	0.00	0.00	0.00	0.00	0.00	0.00	0.00	0.00	0.00	0.00	0.00	0.00	0.00	0.00
Retail trade services, except of motor vehicles and motorcycles	0.00	0.00	0.00	0.00	0.00	0.00	0.00	0.00	0.00	0.00	0.00	0.00	0.00	0.00	0.00	0.00	0.00	0.00	0.00	0.00
Rail transport services	1.29	1.35	1.21	1.06	1.02	1.07	1.06	1.11	1.07	1.00	0.99	0.81	0.67	0.62	0.63	0.63	0.62	0.62	0.60	0.58
Land transport services and transport services via pipelines, excluding rail transport	1.01	1.12	1.02	0.88	0.83	0.88	0.89	0.92	0.85	0.80	0.79	0.74	0.61	0.59	0.60	0.58	0.56	0.55	0.53	0.51
Water transport services	6.68	7.14	7.25	6.57	5.97	6.19	5.76	5.70	5.03	4.68	4.34	3.65	3.34	2.92	2.71	2.71	2.53	2.54	2.46	2.37
Air transport services	3.90	4.13	3.86	3.50	3.42	3.58	3.64	3.81	3.53	3.17	2.98	2.65	2.42	2.17	2.13	2.08	2.00	2.01	1.93	1.86
Warehousing and support services for transportation	0.64	0.67	0.63	0.56	0.52	0.56	0.57	0.60	0.54	0.51	0.49	0.47	0.41	0.39	0.39	0.37	0.39	0.35	0.34	0.33
Postal and courier services	0.16	0.17	0.15	0.16	0.16	0.17	0.16	0.17	0.17	0.21	0.20	0.19	0.16	0.15	0.16	0.14	0.16	0.14	0.13	0.13
Accommodation services	0.54	0.55	0.49	0.46	0.39	0.44	0.46	0.47	0.48	0.48	0.50	0.43	0.35	0.35	0.32	0.30	0.30	0.29	0.29	0.28
Food and beverage serving services	0.54	0.55	0.49	0.46	0.39	0.44	0.46	0.47	0.48	0.48	0.50	0.43	0.35	0.35	0.32	0.30	0.30	0.29	0.29	0.28
Publishing services	0.36	0.36	0.33	0.34	0.34	0.32	0.33	0.35	0.31	0.33	0.34	0.30	0.26	0.25	0.24	0.23	0.24	0.22	0.22	0.21

Sector – Rest-of-World	1997	1998	1999	2000	2001	2002	2003	2004	2005	2006	2007	2008	2009	2010	2011	2012	2013	2014	2015	2016
Motion Picture, Video & TV Programme Production, Sound Recording & Music Publishing Activities & Programming And Broadcasting Activities	0.29	0.30	0.29	0.31	0.33	0.33	0.31	0.33	0.31	0.34	0.33	0.30	0.25	0.25	0.25	0.25	0.25	0.24	0.24	0.23
Telecommunications services	0.16	0.17	0.15	0.16	0.16	0.17	0.16	0.17	0.17	0.21	0.20	0.19	0.16	0.15	0.16	0.14	0.16	0.14	0.13	0.13
Computer programming, consultancy and related services	0.29	0.30	0.29	0.31	0.33	0.33	0.31	0.33	0.31	0.34	0.33	0.30	0.25	0.25	0.25	0.25	0.25	0.24	0.24	0.23
Information services	0.29	0.30	0.29	0.31	0.33	0.33	0.31	0.33	0.31	0.34	0.33	0.30	0.25	0.25	0.25	0.25	0.25	0.24	0.24	0.23
Financial services, except insurance and pension funding	0.30	0.31	0.27	0.27	0.24	0.25	0.25	0.27	0.24	0.24	0.25	0.23	0.19	0.17	0.17	0.16	0.17	0.16	0.16	0.15
Insurance and reinsurance, except compulsory social security & Pension funding	0.40	0.39	0.34	0.30	0.30	0.33	0.32	0.30	0.28	0.27	0.27	0.25	0.21	0.20	0.20	0.20	0.19	0.20	0.20	0.19
Services auxiliary to financial services and insurance services	0.38	0.34	0.29	0.26	0.26	0.28	0.27	0.24	0.23	0.23	0.23	0.22	0.18	0.17	0.17	0.17	0.17	0.17	0.16	0.16
Real estate services, excluding on a fee or contract basis and imputed rent	0.29	0.27	0.24	0.27	0.26	0.27	0.28	0.32	0.31	0.35	0.29	0.26	0.21	0.22	0.21	0.18	0.21	0.19	0.19	0.19
Owner-Occupiers' Housing Services	0.00	0.00	0.00	0.00	0.00	0.00	0.00	0.00	0.00	0.00	0.00	0.00	0.00	0.00	0.00	0.00	0.00	0.00	0.00	0.00
Real estate activities on a fee or contract basis	0.29	0.27	0.24	0.27	0.26	0.27	0.28	0.32	0.31	0.35	0.29	0.26	0.21	0.22	0.21	0.18	0.21	0.19	0.19	0.19
Legal services	0.36	0.36	0.33	0.34	0.34	0.32	0.33	0.35	0.31	0.33	0.34	0.30	0.26	0.25	0.24	0.23	0.24	0.22	0.22	0.21
Accounting, bookkeeping and auditing services; tax consulting services	0.36	0.36	0.33	0.34	0.34	0.32	0.33	0.35	0.31	0.33	0.34	0.30	0.26	0.25	0.24	0.23	0.24	0.22	0.22	0.21
Services of head offices; management consulting services	0.36	0.36	0.33	0.34	0.34	0.32	0.33	0.35	0.31	0.33	0.34	0.30	0.26	0.25	0.24	0.23	0.24	0.22	0.22	0.21
Architectural and engineering services; technical testing and analysis services	0.36	0.36	0.33	0.34	0.34	0.32	0.33	0.35	0.31	0.33	0.34	0.30	0.26	0.25	0.24	0.23	0.24	0.22	0.22	0.21
Scientific research and development services	0.90	0.94	0.96	0.95	0.88	0.89	0.91	1.05	0.96	0.90	0.95	0.81	0.68	0.60	0.58	0.57	0.55	0.56	0.55	0.53
Advertising and market research services	0.36	0.36	0.33	0.34	0.34	0.32	0.33	0.35	0.31	0.33	0.34	0.30	0.26	0.25	0.24	0.23	0.24	0.22	0.22	0.21
Other professional, scientific and technical services	0.36	0.36	0.33	0.34	0.34	0.32	0.33	0.35	0.31	0.33	0.34	0.30	0.26	0.25	0.24	0.23	0.24	0.22	0.22	0.21
Veterinary services	0.36	0.36	0.33	0.34	0.34	0.32	0.33	0.35	0.31	0.33	0.34	0.30	0.26	0.25	0.24	0.23	0.24	0.22	0.22	0.21
Rental and leasing services	0.34	0.34	0.33	0.33	0.33	0.34	0.37	0.40	0.35	0.36	0.36	0.34	0.28	0.27	0.28	0.26	0.27	0.26	0.25	0.25
Employment services	0.36	0.36	0.33	0.34	0.34	0.32	0.33	0.35	0.31	0.33	0.34	0.30	0.26	0.25	0.24	0.23	0.24	0.22	0.22	0.21
Travel agency, tour operator and other reservation services and related services	0.36	0.36	0.33	0.34	0.34	0.32	0.33	0.35	0.31	0.33	0.34	0.30	0.26	0.25	0.24	0.23	0.24	0.22	0.22	0.21
Security and investigation services	0.36	0.36	0.33	0.34	0.34	0.32	0.33	0.35	0.31	0.33	0.34	0.30	0.26	0.25	0.24	0.23	0.24	0.22	0.22	0.21
Services to buildings and landscape	0.36	0.36	0.33	0.34	0.34	0.32	0.33	0.35	0.31	0.33	0.34	0.30	0.26	0.25	0.24	0.23	0.24	0.22	0.22	0.21
Office administrative, office support and other business support services	0.36	0.36	0.33	0.34	0.34	0.32	0.33	0.35	0.31	0.33	0.34	0.30	0.26	0.25	0.24	0.23	0.24	0.22	0.22	0.21
Public administration and defence services; compulsory social security services	0.82	0.87	0.81	0.76	0.75	0.77	0.80	0.83	0.76	0.71	0.71	0.63	0.54	0.49	0.47	0.46	0.46	0.46	0.46	0.45
Education services	0.70	0.73	0.67	0.57	0.57	0.63	0.64	0.65	0.57	0.58	0.58	0.50	0.42	0.40	0.38	0.37	0.37	0.37	0.36	0.35
Human health services	0.62	0.62	0.59	0.58	0.56	0.55	0.55	0.57	0.54	0.52	0.53	0.46	0.39	0.36	0.35	0.34	0.34	0.33	0.32	0.31
Residential Care & Social Work Activities	0.62	0.62	0.59	0.58	0.56	0.55	0.55	0.57	0.54	0.52	0.53	0.46	0.39	0.36	0.35	0.34	0.34	0.33	0.32	0.31
Creative, arts and entertainment services	0.55	0.56	0.54	0.58	0.61	0.62	0.61	0.63	0.59	0.60	0.60	0.51	0.46	0.43	0.43	0.42	0.41	0.42	0.41	0.40
Libraries, archives, museums and other cultural services	0.55	0.56	0.54	0.58	0.61	0.62	0.61	0.63	0.59	0.60	0.60	0.51	0.46	0.43	0.43	0.42	0.41	0.42	0.41	0.40
Gambling and betting services	0.55	0.56	0.54	0.58	0.61	0.62	0.61	0.63	0.59	0.60	0.60	0.51	0.46	0.43	0.43	0.42	0.41	0.42	0.41	0.40
Sports services and amusement and recreation services	0.55	0.56	0.54	0.58	0.61	0.62	0.61	0.63	0.59	0.60	0.60	0.51	0.46	0.43	0.43	0.42	0.41	0.42	0.41	0.40
Services furnished by membership organisations	0.71	0.62 travel	0.50	0.46	0.48	0.51	0.59	0.49	0.45	0.49	0.48	0.48	0.45	0.40	0.39	0.34	0.39	0.35	0.35	0.35
Repair services of computers and personal and household goods	0.51	0.54	0.51	0.49	0.55	0.54	0.56	0.59	0.53	0.57	0.60	0.55	0.46	0.42	0.46	0.44	0.48	0.46	0.45	0.45
Other personal services	0.51	0.54	0.51	0.49	0.55	0.54	0.56	0.59	0.53	0.57	0.60	0.55	0.46	0.42	0.46	0.44	0.48	0.46	0.45	0.45
Services of households as employers of domestic personnel	0.00	0.00	0.00	0.00	0.00	0.00	0.00	0.00	0.00	0.00	0.00	0.00	0.00	0.00	0.00	0.00	0.00	0.00	0.00	0.00

Table S7. UK-MRIO concordance to NHS in England supply chain emissions categories

UK MRIO Sector	Patient travel	Visitor travel	Staff travel	Fleet/ Busi-ness Travel	Elec-tricity	Gas	Oil	Coal	Rene-wables	Pharma-ceuticals	Medical Instruments /equipment	Freight trans-	Business services	Paper prod.s	Other mfg products	Mfg fuels, chemicals & gases	Food & catering	Construction-	ICT	Water	Waste	Other	Commissioned health and social care services
Products of agriculture, hunting and related services	0	0	0	0	0	0	0	0	0	0	0	0	0	0	0	0	1	0	0	0	0	0	0
Products of forestry, logging and related services	0	0	0	0	0	0	0	0	0	0	0	0	0	0	1	0	0	0	0	0	0	0	0
Fish and other fishing products; aquaculture products; support services to fishing	0	0	0	0	0	0	0	0	0	0	0	0	0	0	0	0	1	0	0	0	0	0	0
Coal and lignite	0	0	0	0	0	0	0	1	0	0	0	0	0	0	0	0	0	0	0	0	0	0	0
Crude Petroleum And Natural Gas & Metal Ores	0	0	0	0	0	1	0	0	0	0	0	0	0	0	0	0	0	0	0	0	0	0	0
Other mining and quarrying products	0	0	0	0	0	0	0	0	0	0	0	0	0	0	0	1	0	0	0	0	0	0	0
Mining support services	0	0	0	0	0	0	0	0	0	0	0	0	0	0	0	1	0	0	0	0	0	0	0
Preserved meat and meat products	0	0	0	0	0	0	0	0	0	0	0	0	0	0	0	0	1	0	0	0	0	0	0
Processed and preserved fish, crustaceans, molluscs, fruit and vegetables	0	0	0	0	0	0	0	0	0	0	0	0	0	0	0	0	1	0	0	0	0	0	0
Vegetable and animal oils and fats	0	0	0	0	0	0	0	0	0	0	0	0	0	0	0	0	1	0	0	0	0	0	0
Dairy products	0	0	0	0	0	0	0	0	0	0	0	0	0	0	0	0	1	0	0	0	0	0	0
Grain mill products, starches and starch products	0	0	0	0	0	0	0	0	0	0	0	0	0	0	0	0	1	0	0	0	0	0	0
Bakery and farinaceous products	0	0	0	0	0	0	0	0	0	0	0	0	0	0	0	0	1	0	0	0	0	0	0
Other food products	0	0	0	0	0	0	0	0	0	0	0	0	0	0	0	0	1	0	0	0	0	0	0
Prepared animal feeds	0	0	0	0	0	0	0	0	0	0	0	0	0	0	0	0	1	0	0	0	0	0	0
Alcoholic beverages	0	0	0	0	0	0	0	0	0	0	0	0	0	0	0	0	1	0	0	0	0	0	0
Soft drinks	0	0	0	0	0	0	0	0	0	0	0	0	0	0	0	0	1	0	0	0	0	0	0
Tobacco products	0	0	0	0	0	0	0	0	0	0	0	0	0	0	0	0	1	0	0	0	0	0	0
Textiles	0	0	0	0	0	0	0	0	0	0	0	0	0	0	1	0	0	0	0	0	0	0	0
Wearing apparel	0	0	0	0	0	0	0	0	0	0	0	0	0	0	1	0	0	0	0	0	0	0	0
Leather and related products	0	0	0	0	0	0	0	0	0	0	0	0	0	0	1	0	0	0	0	0	0	0	0
Wood and of products of wood and cork, except furniture; articles of straw and plaiting materials	0	0	0	0	0	0	0	0	0	0	0	0	0	0	1	0	0	0	0	0	0	0	0
Paper and paper products	0	0	0	0	0	0	0	0	0	0	0	0	0	1	0	0	0	0	0	0	0	0	0
Printing and recording services	0	0	0	0	0	0	0	0	0	0	0	0	0	1	0	0	0	0	0	0	0	0	0
Coke and refined petroleum products	0	0	0	0.95	0	0	0.025	0.025	0	0	0	0	0	0	0	0	0	0	0	0	0	0	0
Paints, varnishes and similar coatings, printing ink and mastics	0	0	0	0	0	0	0	0	0	0	0	0	0	0	0	1	0	0	0	0	0	0	0
Soap and detergents, cleaning and polishing preparations, perfumes and toilet preparations	0	0	0	0	0	0	0	0	0	0	0	0	0	0	0	1	0	0	0	0	0	0	0
Other chemical products	0	0	0	0	0	0	0	0	0	0	0	0	0	0	0	1	0	0	0	0	0	0	0
Industrial gases, inorganics and fertilisers (all inorganic chemicals) - 20.11/13/15	0	0	0	0	0	0	0	0	0	0	0	0	0	0	0	1	0	0	0	0	0	0	0
Petrochemicals - 20.14/16/17/60	0	0	0	0	0	0	0	0	0	0	0	0	0	0	0	1	0	0	0	0	0	0	0
Dyestuffs, agro-chemicals - 20.12/20	0	0	0	0	0	0	0	0	0	0	0	0	0	0	0	1	0	0	0	0	0	0	0
Basic pharmaceutical products and pharmaceutical preparations	0	0	0	0	0	0	0	0	0	1	0	0	0	0	0	0	0	0	0	0	0	0	0
Rubber and plastic products	0	0	0	0	0	0	0	0	0	0	0	0	0	0	1	0	0	0	0	0	0	0	0
Manufacture of cement, lime, plaster and articles of concrete, cement and plaster	0	0	0	0	0	0	0	0	0	0	0	0	0	0	0	0	0	1	0	0	0	0	0
Glass, refractory, clay, other porcelain and ceramic, stone and abrasive products - 23.1-4/7-9	0	0	0	0	0	0	0	0	0	0	0	0	0	0	0	0	0	1	0	0	0	0	0
Basic iron and steel	0	0	0	0	0	0	0	0	0	0	0	0	0	0	0	0	0	1	0	0	0	0	0
Other basic metals and casting	0	0	0	0	0	0	0	0	0	0	0	0	0	0	0	0	0	1	0	0	0	0	0
Weapons and ammunition	0	0	0	0	0	0	0	0	0	0	0	0	0	0	0	0	0	1	0	0	0	0	0
Fabricated metal products, excl. machinery and equipment and weapons & ammunition - 25.1-3/25.5-9	0	0	0	0	0	0	0	0	0	0	0	0	0	0	0	0	0	1	0	0	0	0	0

UK MRIO Sector	Patient travel	Visitor travel	Staff travel	Fleet/ Busi-ness Travel	Elec-tricity	Gas	Oil	Coal	Rene-wables	Pharma-ceuticals	Medical Instruments /equipment	Freight trans-	Business services	Paper prod.s	Other mfg products	Mfg fuels, chemicals & gases	Food & catering	Construction-	ICT	Water	Waste	Other	Commissioned health and social care services
Computer, electronic and optical products	0	0	0	0	0	0	0	0	0	0	0.95	0	0	0	0	0	0	0	0.05	0	0	0	0
Electrical equipment	0	0	0	0	0	0	0	0	0	0	1	0	0	0	0	0	0	0	0	0	0	0	0
Machinery and equipment n.e.c.	0	0	0	0	0	0	0	0	0	0	1	0	0	0	0	0	0	0	0	0	0	0	0
Motor vehicles, trailers and semi-trailers	0	0	0	1	0	0	0	0	0	0	0	0	0	0	0	0	0	0	0	0	0	0	0
Ships and boats	0	0	0	1	0	0	0	0	0	0	0	0	0	0	0	0	0	0	0	0	0	0	0
Air and spacecraft and related machinery	0	0	0	1	0	0	0	0	0	0	0	0	0	0	0	0	0	0	0	0	0	0	0
Other transport equipment - 30.2/4/9	0	0	0	1	0	0	0	0	0	0	0	0	0	0	0	0	0	0	0	0	0	0	0
Furniture	0	0	0	0	0	0	0	0	0	0	0	0	0	0	0	0	0	1	0	0	0	0	0
Other manufactured goods	0	0	0	0	0	0	0	0	0	0	0	0	0	0	1	0	0	0	0	0	0	0	0
Repair and maintenance of ships and boats	0	0	0	1	0	0	0	0	0	0	0	0	0	0	0	0	0	0	0	0	0	0	0
Repair and maintenance of aircraft and spacecraft	0	0	0	1	0	0	0	0	0	0	0	0	0	0	0	0	0	0	0	0	0	0	0
Rest of repair; Installation - 33.11-14/17/19/20	0	0	0	0	0	0	0	0	0	0	0	0	0	0	0	0	0	1	0	0	0	0	0
Electricity, transmission and distribution	0	0	0	0	1	0	0	0	0	0	0	0	0	0	0	0	0	0	0	0	0	0	0
Gas; distribution of gaseous fuels through mains; steam and air conditioning supply	0	0	0	0	0	1	0	0	0	0	0	0	0	0	0	0	0	0	0	0	0	0	0
Natural water; water treatment and supply services	0	0	0	0	0	0	0	0	0	0	0	0	0	0	0	0	0	0	0	1	0	0	0
Sewerage services; sewage sludge	0	0	0	0	0	0	0	0	0	0	0	0	0	0	0	0	0	0	0	1	0	0	0
Waste collection, treatment and disposal services; materials recovery services	0	0	0	0	0	0	0	0	0	0	0	0	0	0	0	0	0	0	0	0	1	0	0
Remediation services and other waste management services	0	0	0	0	0	0	0	0	0	0	0	0	0	0	0	0	0	0	0	0	1	0	0
Construction	0	0	0	0	0	0	0	0	0	0	0	0	0	0	0	0	0	1	0	0	0	0	0
Wholesale and retail trade and repair services of motor vehicles and motorcycles	0	0	0	1	0	0	0	0	0	0	0	0	0	0	0	0	0	0	0	0	0	0	0
Wholesale trade services, except of motor vehicles and motorcycles	0	0	0	0	0	0	0	0	0	0	0	0	0	0	0	0	0	0	0	0	0	1	0
Retail trade services, except of motor vehicles and motorcycles	0	0	0	0	0	0	0	0	0	0	0	0	0	0	0	0	0	0	0	0	0	1	0
Rail transport services	0	0	0	1	0	0	0	0	0	0	0	0	0	0	0	0	0	0	0	0	0	0	0
Land transport services and transport services via pipelines, excluding rail transport	0	0	0	0	0	0	0	0	0	0	0	1	0	0	0	0	0	0	0	0	0	0	0
Water transport services	0	0	0	1	0	0	0	0	0	0	0	0	0	0	0	0	0	0	0	0	0	0	0
Air transport services	0	0	0	1	0	0	0	0	0	0	0	0	0	0	0	0	0	0	0	0	0	0	0
Warehousing and support services for transportation	0	0	0	0	0	0	0	0	0	0	0	1	0	0	0	0	0	0	0	0	0	0	0
Postal and courier services	0	0	0	0	0	0	0	0	0	0	0	0	1	0	0	0	0	0	0	0	0	0	0
Accommodation services	0	0	0	0	0	0	0	0	0	0	0	0	1	0	0	0	0	0	0	0	0	0	0
Food and beverage serving services	0	0	0	0	0	0	0	0	0	0	0	0	0	0	0	0	1	0	0	0	0	0	0
Publishing services	0	0	0	0	0	0	0	0	0	0	0	0	0	0	0	0	0	0	1	0	0	0	0
Motion Picture, Video & TV Programme Production, Sound Recording & Music Publishing Activities & Programming And Broadcasting Activities	0	0	0	0	0	0	0	0	0	0	0	0	0	0	0	0	0	0	1	0	0	0	0
Telecommunications services	0	0	0	0	0	0	0	0	0	0	0	0	0	0	0	0	0	0	1	0	0	0	0
Computer programming, consultancy and related services	0	0	0	0	0	0	0	0	0	0	0	0	0	0	0	0	0	0	1	0	0	0	0
Information services	0	0	0	0	0	0	0	0	0	0	0	0	0	0	0	0	0	0	1	0	0	0	0
Financial services, except insurance and pension funding	0	0	0	0	0	0	0	0	0	0	0	0	1	0	0	0	0	0	0	0	0	0	0
Insurance and reinsurance, except compulsory social security & Pension funding	0	0	0	0	0	0	0	0	0	0	0	0	1	0	0	0	0	0	0	0	0	0	0
Services auxiliary to financial services and insurance services	0	0	0	0	0	0	0	0	0	0	0	0	1	0	0	0	0	0	0	0	0	0	0
Real estate services, excluding on a fee or contract basis and imputed rent	0	0	0	0	0	0	0	0	0	0	0	0	1	0	0	0	0	0	0	0	0	0	0
Owner-Occupiers' Housing Services	0	0	0	0	0	0	0	0	0	0	0	0	1	0	0	0	0	0	0	0	0	0	0
Real estate activities on a fee or contract basis	0	0	0	0	0	0	0	0	0	0	0	0	1	0	0	0	0	0	0	0	0	0	0
Legal services	0	0	0	0	0	0	0	0	0	0	0	0	1	0	0	0	0	0	0	0	0	0	0

UK MRIO Sector	Patient travel	Visitor travel	Staff travel	Fleet/ Busi-ness Travel	Elec-tricity	Gas	Oil	Coal	Rene-wables	Pharma-ceuticals	Medical Instruments /equipment	Freight trans-	Business services	Paper prod.s	Other mfg products	Mfg fuels, chemicals & gases	Food & catering	Construction-	ICT	Water	Waste	Other	Commissioned health and social care services
Accounting, bookkeeping and auditing services; tax consulting services	0	0	0	0	0	0	0	0	0	0	0	0	1	0	0	0	0	0	0	0	0	0	0
Services of head offices; management consulting services	0	0	0	0	0	0	0	0	0	0	0	0	1	0	0	0	0	0	0	0	0	0	0
Architectural and engineering services; technical testing and analysis services	0	0	0	0	0	0	0	0	0	0	0	0	0	0	0	0	0	1	0	0	0	0	0
Scientific research and development services	0	0	0	0	0	0	0	0	0	0	0	0	1	0	0	0	0	0	0	0	0	0	0
Advertising and market research services	0	0	0	0	0	0	0	0	0	0	0	0	1	0	0	0	0	0	0	0	0	0	0
Other professional, scientific and technical services	0	0	0	0	0	0	0	0	0	0	0	0	1	0	0	0	0	0	0	0	0	0	0
Veterinary services	0	0	0	0	0	0	0	0	0	0	0	0	1	0	0	0	0	0	0	0	0	0	0
Rental and leasing services	0	0	0	0	0	0	0	0	0	0	0	0	1	0	0	0	0	0	0	0	0	0	0
Employment services	0	0	0	0	0	0	0	0	0	0	0	0	1	0	0	0	0	0	0	0	0	0	0
Travel agency, tour operator and other reservation services and related services	0	0	0	1	0	0	0	0	0	0	0	0	0	0	0	0	0	0	0	0	0	0	0
Security and investigation services	0	0	0	0	0	0	0	0	0	0	0	0	1	0	0	0	0	0	0	0	0	0	0
Services to buildings and landscape	0	0	0	0	0	0	0	0	0	0	0	0	1	0	0	0	0	0	0	0	0	0	0
Office administrative, office support and other business support services	0	0	0	0	0	0	0	0	0	0	0	0	1	0	0	0	0	0	0	0	0	0	0
Public administration and defence services; compulsory social security services	0	0	0	0	0	0	0	0	0	0	0	0	1	0	0	0	0	0	0	0	0	0	0
Education services	0	0	0	0	0	0	0	0	0	0	0	0	1	0	0	0	0	0	0	0	0	0	0
Human health services	0	0	0	0	0	0	0	0	0	0	0	0	0	0	0	0	0	0	0	0	0	0	1
Residential Care & Social Work Activities	0	0	0	0	0	0	0	0	0	0	0	0	0	0	0	0	0	0	0	0	0	0	1
Creative, arts and entertainment services	0	0	0	0	0	0	0	0	0	0	0	0	1	0	0	0	0	0	0	0	0	0	0
Libraries, archives, museums and other cultural services	0	0	0	0	0	0	0	0	0	0	0	0	1	0	0	0	0	0	0	0	0	0	0
Gambling and betting services	0	0	0	0	0	0	0	0	0	0	0	0	1	0	0	0	0	0	0	0	0	0	0
Sports services and amusement and recreation services	0	0	0	0	0	0	0	0	0	0	0	0	1	0	0	0	0	0	0	0	0	0	0
Services furnished by membership organisations	0	0	0	0	0	0	0	0	0	0	0	0	1	0	0	0	0	0	0	0	0	0	0
Repair services of computers and personal and household goods	0	0	0	0	0	0	0	0	0	0	0	0	1	0	0	0	0	0	0	0	0	0	0
Other personal services	0	0	0	0	0	0	0	0	0	0	0	0	1	0	0	0	0	0	0	0	0	0	0
Services of households as employers of domestic personnel	0	0	0	0	0	0	0	0	0	0	0	0	1	0	0	0	0	0	0	0	0	0	0

Table S8. Summary of accounting for transport-related emissions

**Description**	**Scope**	**Carbon footprint category**	**Source**
Fleet vehicles owned or leased by NHS including ambulance services	Scope 1	Business travel	Supply Chain EEIO
Leased vehicles used by essential users for business miles and personal use (for business miles only)	Scope 1	Business travel	Supply Chain EEIO
Dedicated car club vehicles (i.e. where the Trust has secured preferential access to the vehicle) or vehicles hired for >5 days	Scope 1	Business travel	Supply Chain EEIO
Personal vehicles used by staff for business miles (known as grey fleet)	Scope 3	Business travel	Supply Chain EEIO
Public transport, taxis, flights, short term hire vehicles etc. used by staff	Scope 3	Business travel	Supply Chain EEIO
Non-Emergency Patient Transport services provided by a private provider (where paid for by the NHS)	Scope 3	Business services	Supply Chain EEIO
Public transport, taxis, personal vehicles used by patients (where paid for by the NHS)	Scope 3	Business travel	Supply Chain EEIO
Courier, logistics, post etc.	Scope 3	Business services	Supply Chain EEIO
Freight transport	Scope 3	Freight transport	Supply Chain EEIO
Staff commute to and from NHS sites	Scope 3	Personal travel - staff commute	National Travel Survey
Patient travel to and from NHS sites including primary care such as pharmacy and GP and patient transport services not paid for by the NHS	Non-Protocol	Personal travel - Patient travel	National Travel Survey
Visitor travel accompanying patients, visiting patients in hospital, escort etc.	Non-Protocol	Personal travel - Visitor travel	National Travel Survey

Table S9. Average commuter trip distance of health professionals by employment status and region of workplace, 3-year averages, 2011-2017, calculated by the Department for Transport from Table NTS0411

**Outside London**	**Average miles per trip**				
	**2011-2013**	**2012-2014**	**2013-2015**	**2014-2016**	**2015-2017**
**Employment status:**					
**Full-time employment**	8.3	8.2	8.0	8.3	8.8
**Part-time employment**	5.5	5.7	5.9	6.0	6.2
**All people in employment**	7.6	7.7	7.6	7.9	8.3
**Unweighted sample size (individuals):**					
**Full-time employees**	1,126	1,171	1,153	1,181	1,193
**Part-time employees**	565	535	505	488	495
**All types of employment**	1,691	1,706	1,658	1,669	1,688

**London**	**Average miles per trip**				
	**2011-2013**	**2012-2014**	**2013-2015**	**2014-2016**	**2015-2017**
**Employment status:**					
**Full-time employment**	8.4	8.0	8.8	8.9	9.2
**Part-time employment**	6.0	6.4	5.6	5.7	4.7
**All people in employment**	7.9	7.7	8.3	8.5	8.6
**Unweighted sample size (individuals):**					
**Full-time employees**	217	239	245	254	266
**Part-time employees**	77	74	72	69	69
**All types of employment**	294	313	317	323	335

Table S10. Average distance travelled by main mode for selected (health) trip purposes, England, 2002-2016, calculated by Department for Transport from Table NTS0305

	Miles per person per year														
**Purpose / main mode**	**2002**	**2003**	**2004**	**2005**	**2006**	**2007**	**2008**	**2009**	**2010**	**2011**	**2012**	**2013**	**2014**	**2015**	**2016**
**Personal business medical:**															
Walk	3.3	3.0	3.6	2.8	2.9	2.7	3.2	2.6	2.4	2.8	2.5	2.7	2.5	2.6	2.5
Bicycle	-	-	-	-	-	-	-	-	-	-	-	-	-	-	-
Car / van driver	28.6	31.4	30.6	36.1	38.0	38.2	39.7	34.8	39.0	36.9	37.7	39.0	41.9	41.4	41.5
Car / van passenger	19.1	23.3	22.4	27.7	28.6	32.2	28.6	25.4	29.0	27.2	28.8	31.2	31.6	30.2	36.1
Motorcycle	-	-	-	-	-	-	-	-	-	-	-	-	-	-	-
Other private transport	3.3	2.9	2.0	1.1	2.2	2.5	2.7	2.3	3.1	1.5	2.0	2.0	2.9	2.0	2.8
Bus in London	1.2	1.7	1.6	1.4	1.9	1.5	2.0	1.5	1.6	1.5	1.7	2.2	1.9	2.1	1.6
Other local bus	3.7	5.4	3.9	4.8	5.3	5.8	5.3	6.1	5.1	5.6	6.0	6.3	6.2	6.6	5.2
London Underground	0.7	0.6	1.1	-	0.8	-	0.6	0.6	0.7	-	0.9	-	0.9	0.5	1.5
Surface Rail	2.8	2.9	2.1	1.8	5.4	2.7	3.9	1.6	3.1	3.7	4.4	2.8	1.6	2.3	4.8
Other public transport	1.8	3.2	1.1	2.3	1.5	1.8	2.3	4.6	2.3	2.0	1.7	2.4	2.7	2.4	2.5
**All modes**	65	75	69	79	87	88	89	80	87	82	86	89	93	90	99
**Other social:**															
Walk	3.4	3.2	3.5	3.3	4.7	4.4	4.2	4.0	3.3	4.1	2.7	2.4	3.8	3.5	3.9
Bicycle	-	-	-	-	-	-	0.5	0.6	-	-	-	-	0.8	-	-
Car / van driver	38.7	49.7	45.5	43.1	49.8	54.2	43.5	40.9	43.6	38.4	36.4	36.4	38.2	44.0	37.7
Car / van passenger	37.1	44.9	39.5	36.9	55.1	52.8	51.3	38.5	37.3	41.7	36.4	39.7	34.5	48.7	42.1
Motorcycle	-	0.7	-	-	-	-	0.9	0.6	-	-	-	-	0.9	-	0.9
Other private transport	1.6	3.6	5.4	1.4	4.9	3.4	2.6	2.8	0.8	3.0	2.2	1.1	1.0	0.9	1.1
Bus in London	1.4	2.2	1.9	2.3	2.5	2.6	2.0	2.9	2.5	1.6	1.2	1.6	2.4	2.1	2.1
Other local bus	4.8	3.8	4.0	4.0	6.2	6.3	5.5	5.8	4.3	5.2	4.7	4.1	5.2	6.0	4.0
London Underground	2.4	1.8	1.8	2.4	2.7	3.5	2.7	3.4	1.8	2.3	2.2	1.3	3.2	2.0	2.2
Surface Rail	10.4	6.6	13.8	9.2	13.0	11.0	8.7	9.8	10.9	12.8	10.1	9.2	12.5	7.6	13.2
Other public transport	6.7	7.6	8.7	9.7	6.5	9.0	6.9	6.8	7.3	6.1	6.3	6.5	7.0	6.5	6.3
**All modes**	107	124	125	113	146	148	129	116	112	116	103	103	109	122	114
**Escort shopping/personal business:**															
Walk	5.9	5.0	4.7	5.0	6.0	4.8	5.5	5.9	5.4	5.6	4.9	4.9	4.4	5.8	5.2
Bicycle	-	-	-	-	-	-	-	-	-	-	-	-	-	-	-
Car / van driver	57.2	48.8	50.9	57.4	49.2	52.5	58.0	50.4	48.4	43.5	46.2	47.5	53.0	46.6	49.4
Car / van passenger	81.8	84.3	74.1	73.5	77.1	68.3	96.1	87.5	86.6	86.3	70.7	75.7	75.4	80.8	70.4
Motorcycle	-	0.0	-	0.0	-	0.0	0.0	0.0	0.0	-	-	0.0	0.0	-	-
Other private transport	-	-	-	0.6	-	1.1	0.8	1.8	3.6	0.6	1.4	2.2	-	-	1.6
Bus in London	1.9	1.1	1.1	1.2	1.1	1.2	1.4	1.3	1.3	1.4	1.5	2.3	1.3	2.3	1.2
Other local bus	4.5	3.8	3.6	3.7	3.4	3.5	3.6	4.6	3.7	4.6	4.0	3.2	4.8	4.4	3.1
London Underground	-	-	-	-	0.6	-	-	0.6	0.6	-	0.7	-	0.6	-	0.9
Surface Rail	2.3	0.6	2.3	1.1	1.9	3.4	2.7	3.4	3.7	3.1	2.3	3.1	2.7	3.3	2.6
Other public transport	1.7	0.8	0.5	1.0	1.1	0.9	2.4	1.5	1.3	1.4	1.4	2.4	1.5	1.3	1.4
**All modes**	157	145	138	144	141	136	171	157	155	147	133	142	144	146	136
**Unweighted sample size: individuals**	14,369	16,685	16,487	16,956	16,648	16,858	16,360	17,299	16,553	15,730	16,670	16,192	16,491	15,525	15,840

Table S11. Travel emission intensities, kg CO_2e/km

	2002	2003	2004	2005	2006	2007	2008	2009	2010	2011	2012	2013	2014	2015	2016	2017	2018	2019
**Scope 1**																		
Walk	-	-	-	-	-	-	-	-	-	-	-	-	-	-	-	-	-	-
Bicycle	-	-	-	-	-	-	-	-	-	-	-	-	-	-	-	-	-	-
Car / van driver	0.22	0.22	0.22	0.22	0.22	0.22	0.21	0.20	0.20	0.19	0.19	0.19	0.19	0.19	0.18	0.18	0.17	0.17
Car / van passenger	0.11	0.11	0.11	0.11	0.11	0.11	0.11	0.10	0.10	0.10	0.09	0.09	0.09	0.09	0.09	0.09	0.09	0.08
Motorcycle	0.12	0.12	0.12	0.12	0.12	0.12	0.12	0.12	0.12	0.12	0.12	0.12	0.12	0.12	0.12	0.12	0.12	0.12
Other private transport	0.22	0.22	0.22	0.22	0.22	0.22	0.21	0.20	0.20	0.19	0.14	0.14	0.18	0.17	0.17	0.16	0.16	0.15
Bus in London	0.08	0.08	0.08	0.08	0.08	0.08	0.08	0.08	0.08	0.08	0.08	0.08	0.08	0.08	0.09	0.10	0.11	0.12
Other local bus	0.11	0.11	0.11	0.11	0.11	0.11	0.11	0.11	0.11	0.11	0.12	0.12	0.11	0.11	0.10	0.10	0.09	0.08
London Underground	0.06	0.06	0.06	0.06	0.06	0.06	0.06	0.06	0.06	0.06	0.06	0.06	0.06	0.06	0.05	0.04	0.04	0.03
Surface Rail	0.05	0.05	0.05	0.05	0.05	0.05	0.05	0.05	0.05	0.05	0.05	0.05	0.05	0.05	0.04	0.04	0.04	0.04
Other public transport	0.12	0.12	0.12	0.12	0.12	0.12	0.12	0.12	0.12	0.12	0.06	0.06	0.06	0.05	0.05	0.04	0.04	0.04
**Scope 3**																		
Walk	-	-	-	-	-	-	-	-	-	-	-	-	-	-	-	-	-	-
Bicycle	-	-	-	-	-	-	-	-	-	-	-	-	-	-	-	-	-	-
Car / van driver	0.04	0.04	0.04	0.04	0.04	0.04	0.04	0.04	0.04	0.04	0.04	0.04	0.04	0.04	0.04	0.04	0.04	0.05
Car / van passenger	0.02	0.02	0.02	0.02	0.02	0.02	0.02	0.02	0.02	0.02	0.02	0.02	0.02	0.02	0.02	0.02	0.02	0.02
Motorcycle	0.02	0.02	0.02	0.02	0.02	0.02	0.02	0.02	0.02	0.02	0.02	0.02	0.02	0.02	0.02	0.03	0.03	0.03
Other private transport	0.04	0.04	0.04	0.04	0.04	0.04	0.04	0.04	0.04	0.04	0.03	0.03	0.04	0.04	0.04	0.04	0.04	0.04
Bus in London	0.02	0.02	0.02	0.02	0.02	0.02	0.02	0.02	0.02	0.02	0.02	0.02	0.02	0.02	0.02	0.02	0.02	0.02
Other local bus	0.02	0.02	0.02	0.02	0.02	0.02	0.02	0.02	0.02	0.02	0.03	0.03	0.02	0.02	0.02	0.03	0.03	0.03
London Underground	0.01	0.01	0.01	0.01	0.01	0.01	0.01	0.01	0.01	0.01	0.01	0.01	0.01	0.01	0.01	0.01	0.01	0.00
Surface Rail	0.01	0.01	0.01	0.01	0.01	0.01	0.01	0.01	0.01	0.01	0.01	0.01	0.01	0.01	0.01	0.01	0.01	0.01
Other public transport	0.02	0.02	0.02	0.02	0.02	0.02	0.02	0.02	0.02	0.02	0.01	0.01	0.01	0.01	0.01	0.01	0.01	0.00

Table S12. NHS in England Carbon Footprint by Source Contribution, 1990-2019, in Mt CO_2e

	1990	1991	1992	1993	1994	1995	1996	1997	1998	1999	2000	2001	2002	2003	2004	2005	2006	2007	2008	2009	2010	2011	2012	2013	2014	2015	2016	2017	2018	2019
**Delivery of Care**	**16.2**	**15.6**	**15.3**	**14.1**	**14.0**	**13.1**	**12.9**	**12.2**	**12.5**	**12.3**	**11.4**	**11.0**	**10.6**	**10.8**	**10.1**	**10.7**	**10.3**	**9.8**	**9.5**	**9.4**	**8.7**	**8.3**	**8.8**	**8.2**	**7.8**	**7.4**	**7.0**	**6.7**	**6.4**	**6.1**
Electricity - scope 2 & 3	2.55	2.47	2.36	2.23	2.01	1.88	1.75	1.51	1.64	1.61	1.71	2.00	2.14	2.25	2.33	2.46	2.49	2.32	2.34	2.20	2.31	2.22	2.45	2.42	2.18	1.62	1.18	1.00	0.94	0.70
Natural gas - scope 1 & 3	1.79	1.76	1.73	1.70	1.90	1.68	1.86	1.66	1.90	1.80	1.70	1.98	2.06	1.95	1.91	2.08	1.99	2.02	2.16	2.19	2.20	2.04	2.20	2.14	1.95	2.10	2.11	2.08	1.82	1.78
Oil - scope 1 & 3	1.74	1.57	1.41	1.24	1.08	0.74	0.61	0.43	0.44	0.41	0.41	0.31	0.28	0.26	0.21	0.20	0.13	0.12	0.12	0.10	0.08	0.05	0.05	0.04	0.04	0.03	0.02	0.02	0.03	0.02
Coal - scope 1 & 3	0.90	0.75	0.60	0.45	0.52	0.46	0.42	0.33	0.31	0.27	0.27	0.26	0.25	0.21	0.18	0.19	0.13	0.12	0.09	0.08	0.06	0.04	0.04	0.03	0.03	0.02	0.02	0.01	0.01	0.01
Anaesthetic gases	0.47	0.46	0.45	0.45	0.46	0.45	0.45	0.44	0.44	0.43	0.43	0.43	0.42	0.45	0.45	0.46	0.46	0.46	0.48	0.49	0.50	0.50	0.51	0.50	0.51	0.52	0.52	0.51	0.51	0.48
Metered dose inhalers	4.64	4.40	4.15	3.90	3.65	3.40	3.15	2.90	2.65	2.40	2.15	1.90	1.65	1.40	1.15	0.90	0.65	0.63	0.63	0.67	0.70	0.70	0.72	0.72	0.74	0.75	0.75	0.74	0.79	0.80
Waste products and recycling	2.30	2.38	2.66	2.36	2.50	2.57	2.74	2.91	3.08	3.18	2.78	2.32	2.08	2.52	2.11	2.67	2.70	2.26	1.81	1.75	1.26	1.24	1.21	0.64	0.60	0.62	0.68	0.66	0.66	0.66
Water and sanitation	0.95	0.97	1.07	0.93	0.98	1.02	1.08	1.11	1.10	1.26	1.07	0.93	0.82	0.86	0.74	0.81	0.84	0.84	0.82	0.83	0.63	0.62	0.69	0.68	0.63	0.66	0.67	0.65	0.65	0.65
Business travel and fleet transport	0.85	0.85	0.86	0.86	0.87	0.87	0.88	0.86	0.95	0.95	0.84	0.85	0.88	0.92	1.02	0.91	0.90	1.06	1.02	1.04	0.96	0.93	0.93	1.00	1.09	1.08	1.03	0.99	1.00	1.00
**Supply Chain**	**15.6**	**15.2**	**15.9**	**14.4**	**14.1**	**14.9**	**13.9**	**14.8**	**12.9**	**13.3**	**13.5**	**13.2**	**13.0**	**13.4**	**14.3**	**14.0**	**13.7**	**15.4**	**16.1**	**16.1**	**16.3**	**15.6**	**15.7**	**16.4**	**16.5**	**16.5**	**16.0**	**15.7**	**15.6**	**15.6**
Pharmaceuticals and chemicals	6.84	6.60	6.86	6.23	6.06	6.87	5.68	6.47	4.96	5.08	5.11	5.20	4.85	5.17	5.53	5.42	5.26	5.40	5.36	5.29	5.64	5.49	5.32	5.16	4.88	4.94	5.11	5.09	5.07	5.06
Medical instruments	2.87	2.93	3.24	3.02	2.85	3.06	3.24	3.37	3.18	3.31	3.56	3.34	3.38	3.32	3.33	3.19	3.08	3.46	3.36	3.26	3.27	3.25	3.36	3.21	3.31	3.27	3.10	3.00	2.99	2.98
Non-medical equipment	0.82	0.79	0.81	0.70	0.69	0.66	0.64	0.63	0.77	0.83	0.92	0.84	0.93	1.01	1.08	1.08	1.03	1.32	1.42	1.35	1.31	1.21	1.21	1.45	1.60	1.64	1.56	1.51	1.51	1.50
Business services	1.38	1.36	1.46	1.35	1.29	1.32	1.31	1.43	1.27	1.30	1.26	1.25	1.26	1.31	1.47	1.73	1.78	2.04	2.30	2.64	2.55	2.52	2.86	3.14	3.11	3.09	2.82	2.73	2.72	2.71
Food and catering	2.18	2.07	2.06	1.76	1.83	1.75	1.67	1.55	1.26	1.33	1.27	1.21	1.13	1.17	1.38	1.27	1.22	1.61	1.91	1.74	1.71	1.42	1.31	1.58	1.66	1.64	1.60	1.55	1.55	1.54
Other procurement	1.46	1.43	1.46	1.36	1.36	1.28	1.39	1.30	1.42	1.45	1.41	1.38	1.49	1.42	1.50	1.27	1.28	1.52	1.78	1.80	1.81	1.67	1.65	1.87	1.97	1.90	1.85	1.79	1.78	1.78
**Commissioned Healthcare**	**0.19**	**0.2**	**0.2**	**0.2**	**0.2**	**0.3**	**0.3**	**0.3**	**0.3**	**0.3**	**0.3**	**0.3**	**0.3**	**0.4**	**0.4**	**0.4**	**0.4**	**0.5**	**0.5**	**0.6**	**0.7**	**1.1**	**1.1**	**1.3**	**1.2**	**1.1**	**1.0**	**1.0**	**1.0**	**1.0**
**Travel**	**1.9**	**2.0**	**2.0**	**2.0**	**2.1**	**2.1**	**2.1**	**2.2**	**2.2**	**2.2**	**2.3**	**2.3**	**2.2**	**2.4**	**2.3**	**2.5**	**2.6**	**2.7**	**2.6**	**2.3**	**2.5**	**2.3**	**2.3**	**2.4**	**2.4**	**2.4**	**2.4**	**2.4**	**2.4**	**2.4**
Staff commute	0.86	0.94	0.95	0.96	0.97	0.98	0.99	0.99	1.00	1.01	1.02	1.03	0.98	1.02	1.05	1.13	1.05	1.10	1.02	0.95	1.03	0.99	0.96	0.95	0.93	0.90	0.91	0.92	0.90	0.88
Patient travel	0.63	0.65	0.67	0.69	0.71	0.74	0.76	0.78	0.81	0.83	0.86	0.88	0.84	0.96	0.89	1.05	1.14	1.18	1.16	1.02	1.12	1.01	1.06	1.11	1.18	1.12	1.19	1.21	1.23	1.23
Visitor travel	0.39	0.39	0.39	0.38	0.38	0.38	0.38	0.38	0.38	0.38	0.38	0.38	0.35	0.37	0.36	0.36	0.39	0.40	0.40	0.35	0.35	0.32	0.30	0.31	0.33	0.33	0.31	0.31	0.30	0.29
**NHS in England TOTAL**	**33.8**	**33.0**	**33.4**	**30.8**	**30.3**	**30.4**	**29.3**	**29.3**	**27.8**	**28.2**	**27.5**	**26.8**	**26.1**	**27.0**	**27.1**	**27.6**	**27.0**	**28.3**	**28.7**	**28.4**	**28.1**	**27.3**	**27.9**	**28.3**	**28.0**	**27.3**	**26.4**	**25.7**	**25.4**	**25.0**
Social Care and Public Health	6.24	5.92	5.94	5.68	5.73	6.02	5.88	6.43	6.15	5.88	5.76	5.80	5.57	5.89	6.47	6.68	6.45	6.54	6.67	5.97	6.01	6.00	6.24	5.66	6.26	6.29	5.91	6.31	6.26	5.40
**England TOTAL**	**40.1**	**38.9**	**39.3**	**36.5**	**36.1**	**36.4**	**35.1**	**35.8**	**34.0**	**34.0**	**33.2**	**32.6**	**31.7**	**32.8**	**33.6**	**34.3**	**33.4**	**34.9**	**35.4**	**34.3**	**34.2**	**33.3**	**34.2**	**33.9**	**34.2**	**33.6**	**32.3**	**32.1**	**31.7**	**30.4**

Table S13. NHS in England carbon footprint per inhabitant, real unit of spend, and inpatient Finished Admission Episode (FAE)

Year	Total GHG emissions (Mt CO2e)	GHG emissions per inhabitant (kg CO2e /inhabitant)	GHG emissions per real unit of spend (Mt CO2e / billion 2018/19 GBP)	Emissions per inpatient Finished Admission Episode (kg CO2e/ FAE)
**1990**	33.8	709	0.7	40208
**1991**	33.0	689	0.6	38443
**1992**	33.4	696	0.6	36475
**1993**	30.8	640	0.5	32117
**1994**	30.3	629	0.5	31077
**1995**	30.4	628	0.5	29628
**1996**	29.3	603	0.5	28536
**1997**	29.3	603	0.4	27764
**1998**	27.8	570	0.4	25270
**1999**	28.2	574	0.4	25258
**2000**	27.5	558	0.4	24715
**2001**	26.8	542	0.3	24204
**2002**	26.1	526	0.3	22988
**2003**	27.0	540	0.3	22827
**2004**	27.1	540	0.2	22394
**2005**	27.6	546	0.2	21778
**2006**	27.0	529	0.2	20772
**2007**	28.3	552	0.2	21024
**2008**	28.7	554	0.2	20278
**2009**	28.4	544	0.2	19515
**2010**	28.1	535	0.2	18899
**2011**	27.3	514	0.2	18188
**2012**	27.9	522	0.2	18429
**2013**	28.3	525	0.2	18289
**2014**	28.0	515	0.2	17610
**2015**	27.3	498	0.2	16790
**2016**	26.4	478	0.2	15973

**2017**	25.7	463	0.2	15487
**2018**	25.4	454	0.2	14837
**2019**	25.0	445	0.2	14556

Section S1. GHG emissions accounting methods description

For each NHS emissions category i, emissions E_i are calculated by multiplying the quantity of consumption M_i by the corresponding emissions factor $(\mathrm{EF})_i$:

$$E_i = M_i \times (\mathrm{EF})_i$$

Data sources for the quantity of consumption and corresponding emissions factors are provided in the main text and associated Supplementary Appendix tables, summarized again here.

- **Buildings**. Emissions factors for fuels and electricity are taken from the UK Government Energy (BEIS) and Environment (DEFRA) ministry publications for 2002-2019, and from company reporting guidance for older calculations (Table S4).[1,2]
- **Anaesthetic gases**. Emissions factors for the volatiles (sevoflurane, isoflurane, desflurane) are taken from Sulbaek-Anderson (2011) and for N_2O from the IPCC AR5 report.[3,4]
- **Metered dose inhalers**. Emissions factors for all propellants are from the IPCC AR5 report.[4]
- **Transport**. Emissions factors are taken from BEIS including both scope 1 (direct emissions) and scope 3 (well-to-tank emissions) (Table S11).[1]
- **Supply chain**. Emissions factors are calculated from UK MRIO model as follows, using standard input-output techniques. **A** is the direct requirements matrix for the global economy, relating the sector output from each sector of the UK economy and three regional economies (EU, China, and Rest-of-World), 106 sectors in each region for a total of 424 sectors. Let Y_j be the vector of unit final demand for sector j (final demand of unity in sector j and zeros for all other sectors). The vector of required output from each sector X_j is then:

  $$X_j = [\mathbf{I} - \mathbf{A}]^{-1} Y_j$$

  where ***I*** is the identity matrix.

  Let R be a vector of direct GHG emissions in kg CO_2e per unit of monetary output (£) in each sector of the UK MRIO model, as compiled from national emissions inventories. Then the supply chain emissions factor $(\mathrm{EF})_j$ of kg CO_2e per unit of healthcare expenditure in sector j is given by:

  $$(\mathrm{EF})_j = RX_j = R[\mathbf{I} - \mathbf{A}]^{-1} Y_j$$

  These supply chain emissions factors are provided in Table S6.

Section S2. Forecasting / Backcasting methods description

Forecasting and backcasting for bottom-up estimation categories was done using linear extrapolation or by scaling based on changes in measurable quantities, including hospital energy use, expenditures, or clinical activity, expressed as Q. Emissions E_i^m for sector i in year m are backcast or forecast to year n to determine emissions in that year E_i^n, according to:

$$E_i^n = E_i^m \times \left(\frac{Q^n}{Q^m}\right)$$

Forecasting for top-down estimation categories was performed through the following steps.

Health expenditure forecasts are expressed as a percentage of GDP for each future year t (from 2019):

$$\mathrm{GDP}(t) = \mathrm{GDP}(t-1) \times \frac{\mathrm{GDPdeflator}(t)}{\mathrm{GDPdeflator}(t-1)} \times \frac{100}{100 + \mathrm{CPI}(t)}$$

Based on this the health expenditure can be calculated and then NHS expenditure as a static proportion of health expenditure (other health expenditure has been assumed to remain a static percentage).

Health Expenditure future time series (from 2016 for consistent time series):

$$\text{Health Expenditure } (t) = \mathrm{GDP}(t) \times \text{Health \% of GDP } (t)$$

NHS Expenditure:

$$\text{NHS Expenditure } (t > 2016) = \frac{\text{Health Expenditure } (t) \times \text{NHS Expenditure } (t-1)}{\text{Health Expenditure } (t-1)}$$

The NHS expenditure forecast is then split into the emissions categories used in the carbon footprint:

$$\text{Expenditure } (t, \text{category } x) = \text{NHS Expenditure } (t) \times \sum_{\mathrm{SIC}=1}^{\mathrm{SIC}=109} \text{concordance } (\mathrm{SIC}, x) \times \text{proportion of spend } (\mathrm{SIC}, t)$$

Some expenditure categories are excluded where bottom-up information replaces the MRIO estimates to remove duplication. Expenditure categories included in the model are totalled up to

give NHS procurement spend. Multipliers were created to estimate time series GDP growth and inflation of NHS procurement spend.

$$\text{Multiplier } (t < 2015) = \prod_{t+1}^{t=2015} \text{average}\left(\frac{\text{GDP}(t) * \text{GDP multipler}}{\text{GDP}(t-1)}, \frac{\text{NHS Expenditure}(t)}{\text{NHS Expenditure}(t-1)}\right)$$

GDP multiplier is set so that expenditure in the period 1990-2015 is flattened:

$$\text{Multiplier } (t > 2015) = \frac{1}{\prod_{t=2015}^{t} \text{average}\left(\frac{\text{GDP}(t) * \text{GDP multipler}}{\text{GDP}(t-1)}, \frac{\text{NHS Expenditure}(t)}{\text{NHS Expenditure}(t-1)}\right)}$$

Expenditure is then flattened based on GDP and NHS expenditure growth to reduce the number of variables in the model. This was used to create a time series from 1990 to latest year capturing only year-to-year fluctuations in spend by the NHS. An accelerator was introduced to represent the decoupling of growth and carbon intensity. The value of this accelerator was set so that expenditure was flat:

$$\text{Accelerator} = \lambda \times \frac{\sum_{t=1990}^{t=2016} \text{GDP multiplier } (t) - \sum_{t=1990}^{t=2016} \text{NHS multiplier } (t)}{t}$$

The value of λ was set so that the following chart was flat, the value was 1 for the final model.

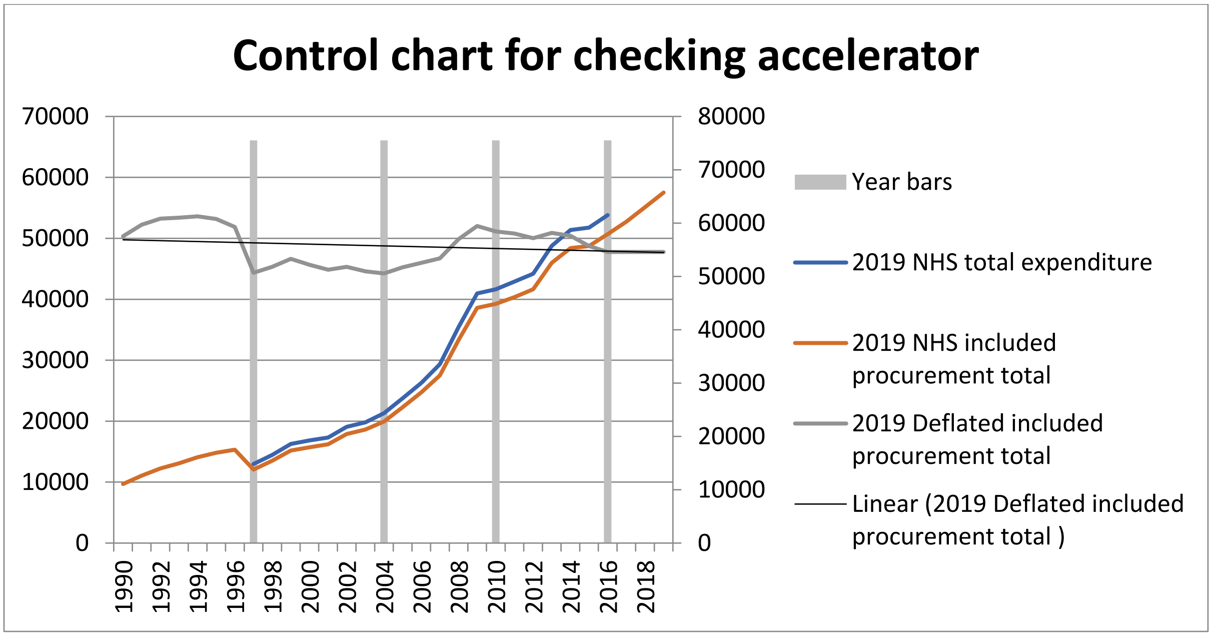

Deflated procurement was calculated by flattening expenditure as follows:

$$\text{NHS procurement spend flat } (t \le 2016) = \text{ NHS procurement spend } (t) \times \text{Multiplier } (t) \times \text{Accelerator}$$

$$\text{NHS procurement spend flat } (t > 2016) = \text{ NHS procurement spend flat } (t-1)$$

From this, flattened carbon intensities were calculated:

$$\text{Intensity flat } (t \le 2016, \text{category } x) = \frac{\text{Emissions } (t, \text{category } x)}{\text{NHS procurement spend flat}(t)}$$

$$\text{Intensity flat } (t > 2016, \text{category } x) = \text{growth (Intensity flat } (2008 \rightarrow 2016), t)$$

(*n.b.*, this is using the whole procurement expenditure and therefore is not the category carbon intensity and is only used for total procurement emissions. Lower levels of carbon intensity were considered too volatile to forecast individually)

Using flattened carbon intensities and flattened expenditure, the carbon emissions forecast was then created:

$$\text{Carbon emissions } (t > 2016, \text{category } x) = \text{Intensity flat } (t, \text{category } x) \times \text{NHS procurement spend flat } (t)$$

References

1 BEIS. Greenhouse gas reporting: conversion factors 2019 - GOV.UK. GOV.UK. 2019.

2 Department for Environment Food and Rural Affairs. 2011 Guidelines to Defra / DECC's GHG Conversion Factors for Company Reporting: Methodology Paper for Emission Factors. 2011.

3 Sulbaek Andersen MP, Nielsen OJ, Karpichev B, Wallington TJ, Sander SP. Atmospheric chemistry of isoflurane, desflurane, and sevoflurane: kinetics and mechanisms of reactions with chlorine atoms and OH radicals and global warming potentials. *J Phys Chem A* 2011; **116**: 5806–20.

4 IPCC. IPCC Fifth Assessment Synthesis Report-Climate Change 2014 Synthesis Report. *IPCC Fifth Assess Synth Report-Climate Chang 2014 Synth Rep* 2014.
